# *Euphorbia* Diterpenes: An Update of Isolation, Structure, Pharmacological Activities and Structure–Activity Relationship

**DOI:** 10.3390/molecules26165055

**Published:** 2021-08-20

**Authors:** Douglas Kemboi, Xavier Siwe-Noundou, Rui W. M. Krause, Moses K. Langat, Vuyelwa Jacqueline Tembu

**Affiliations:** 1 Department of Chemistry, Faculty of Science, Tshwane University of Technology, Pretoria 0001, South Africa; 2 Department of Chemistry, Rhodes University, Makhanda 6140, South Africa; r.krause@ru.ac.za; 3 Jodrell Laboratory, Department of Unlocking Properties, Royal Botanic Gardens Kew, Richmond TW9 3DS, UK; m.langat@kew.org

**Keywords:** *Euphorbia*, diterpenes, pharmacological activity, structure–activity relationship

## Abstract

*Euphorbia* species have a rich history of ethnomedicinal use and ethnopharmacological applications in drug discovery. This is due to the presence of a wide range of diterpenes exhibiting great structural diversity and pharmacological activities. As a result, *Euphorbia* diterpenes have remained the focus of drug discovery investigations from natural products. The current review documents over 350 diterpenes, isolated from *Euphorbia* species, their structures, classification, biosynthetic pathways, and their structure–activity relationships for the period covering 2013–2020. Among the isolated diterpenes, over 20 skeletal structures were identified. Lathyrane, jatrophane, ingenane, ingenol, and ingol were identified as the major diterpenes in most *Euphorbia* species. Most of the isolated diterpenes were evaluated for their cytotoxicity activities, multidrug resistance abilities, and inhibitory activities in vitro, and reported good activities with significant half-inhibitory concentration (IC_50_) values ranging from 10–50 µM. The lathyranes, isopimaranes, and jatrophanes diterpenes were further found to show potent inhibition of P-glycoprotein, which is known to confer drug resistance abilities in cells leading to decreased cytotoxic effects. Structure–activity relationship (SAR) studies revealed the significance of a free hydroxyl group at position C-3 in enhancing the anticancer and anti-inflammatory activities and the negative effect it has in position C-2. Esterification of this functionality, in selected diterpenes, was found to enhance these activities. Thus, *Euphorbia* diterpenes offer a valuable source of lead compounds that could be investigated further as potential candidates for drug discovery.

## 1. Introduction

*Euphorbia* species have a rich history of ethnomedicinal use, as well as ethnopharmacological applications in drug discovery from ancient times to the present [1,2,3]. Plants of the *Euphorbia* genus are popular medicinal herbs applied in the prevention and treatment of diseases, like respiratory diseases, body/skin pain and irritations, indigestion disorders, inflammation, cancer, microbial infestations such as HIV, and gonorrhea, and eye disorders [2,4,5]. As early as the era before Christ (BC), *Euphorbia* species were utilized in the treatment of liver diseases, snake bites, sprains, convulsions, asthma, tumors, and rheumatisms in the Ayurvedic and Chinese medicine systems [1,2,4,6].

Reported evidence shows that medicinal usages of *Euphorbia* species are recorded worldwide and utilize the stems, stem barks, whole plant, latex, aerial part, seeds, leaves, and roots, with *E. lathyris*, *E. maculata*, and *E. hirta* as the most frequently used species [1,2,7]. Their classification, chemistry, and medicinal applications are ascribed to the presence of many phytochemical constituents, such as terpenes [6,8,9,10]. Therefore, *Euphorbia* species remain a rich source of diverse natural products, with a wide range of pharmacological applications that can provide promising lead compounds for drugs and therapeutic agents’ discoveries. The genus *Euphorbia* is amongst the largest genera in the Euphorbiaceae family of flowering plants, consisting of several sections and subgenera with over 2000 species [9,11,12]. Thus it is complex, and of immense research potential.

The genus *Euphorbia* is also known for the structural diversity of its isoprenoids, with most of them being macrocyclic diterpenes [9,13,14]. These diterpenes are the major chemical constituents in the genus and are known to occur in different types of core skeletal structures/frameworks, such as abietanes, tiglianes, ingenanes, daphnanes, lathyranes, jatrophane, myrsinols, and cembranes [10,14,15] amongst others. Jatrophanes, tiglianes, and lathyranes type diterpenoids are the main chemical constituents reported in the genus [9,13]. As a consequence, significant efforts have been made in the isolation and identification of these chemical constituents from the roots, aerial, stems, seeds, stem barks and whole plants of *Euphorbia* species. 

In addition, most of the reported *Euphorbia* diterpenes exhibited a wide range of pharmacological activities such as cytotoxic, anti-inflammatory properties, anti-HIV, tumor-promoting abilities, and antibacterial properties [9,10,13,14]. As a successful example of drug development from naturally derived diterpenes, taxol and taxol derivatives are presently in wide use for cancer treatment [10]. Furthermore, the latest release of ingenol metabutate (picato) a diterpene isolated from *E. peplus* for treatment of actinic keratosis [5,16] has revitalized interest in the phytochemistry of *Euphorbia* species. 

Diterpenes occurring in *Euphorbia* species are classified as either higher or lower diterpenes, and have diverse skeletal frameworks such as tigliane, ingenane, and daphnane [9,13]. Lower diterpenes are limited to the Euphorbiaceae and Thymeleaceae families. *Euphorbia* diterpenes can therefore offer better alternatives for the development of more selective and high potency prodrug derivatives based on their structure–activity relationships. 

The different skeletal frameworks of *Euphorbia* diterpenes are derived from geranylgeranyl pyrophosphate (GGPP) and are subsequently categorized according to their biosynthetic pathways and cyclization patterns as acyclic; like phytanes, bicyclic; like labdanes, clerodanes and halimanes, tricyclic; including abietanes, rosanes, pimaranes, and cassanes, tetracyclic; like kauranes, atisanes and gibberellins and macrocyclic diterpenes including taxanes, daphnanes, cembranes, ingenanes and tiglianes [14,17,18,19]. The detailed information about the biosynthetic pathway and classification of *Euphorbia* diterpenes is not dealt with within this review article and the reader can consult the references for detailed information.

That said, several review publications have summarized scientific reports about the phytochemical constituents of *Euphorbia* species. Most of the published reviews exclusively focused on partial studies about the chemical constituents, biological activities, and synthesis. For instance, Goel et al. [20] reviewed esters of phorbol highlighting the structural diversity, mode of action, toxic effects in animals, and how the compounds can be detoxified from the *Jatropha* species, of the Euphorbiaceae family. Shi et al. [13], reviewed the chemical and pharmacological activities of *Euphorbia* species chemical constituents covering the period 1998 to 2008. Previously undescribed diterpenes and common diterpenes isolated within the review period (1998–2008) were discussed in the review. Vasas and Hohmann [9] reviewed the *Euphorbia* diterpenes isolated from *Euphorbia* species between 2008 and 2012, highlighting their structural diversity and pharmacological activities [9]. Different classes of *Euphorbia* diterpenes and biological activities reported within this period were reviewed. Wang et al. [21] reviewed the tigliane-type diterpenoids from the Euphorbiaceae and Thymelaeaceae families and their biological activities. Wongrakpanich et al. [22] reviewed the induction of apoptosis in cancer cells by chemical constituents isolated from *Euphorbia* species. Jin et al. [14] reported daphnane-type diterpenes of the Euphorbiaceae and Thymelaeaceae families and their pharmacological activities, while Salehi et al. [23] reported the essential oil constituents of *Euphorbia* species. In addition, in our previous study we reviewed the ethnomedicinal uses, triterpenoids of *Euphorbia* species, and their pharmacological activities [24]. 

In most of the reviewed publications, emphasis was given to a specific subclass of diterpenes isolated in *Euphorbia* species, their chemical structures, and reported biological activities within the review period (1998–2014) with limited reference to the structure–activity relationship of these constituents, and that there is no comprehensive review of previously undescribed *Euphorbia* diterpenes covering the period from 2013–2020. Hence, to gain a more comprehensive insight on the latest information about the structural diversity of *Euphorbia* diterpenes, the current review reports the structures, occurrence, classification, and pharmacological activities of newly isolated *Euphorbia* diterpenes, their mechanisms of action, and the structure–activity relationships reported between June 2013 and October 2020. It is hoped that the review on the *Euphorbia* diterpenes will enrich the existing databases with the latest information about the structural diversity of *Euphorbia* diterpenes and their structure–activity relationships, which will help in identifying potential hit or lead compounds for drug discovery. 

## 2. Literature Sources and Search Strategy

To gather all the relevant information about *Euphorbia* diterpenes, their pharmacological activities, and structure–activity relationships, the following online databases were used; Scifinder, Scopus, Springer Link, Science Direct, Wiley online, PubMed, Google Scholar, and Web of Science. The databases were systematically searched for articles published from 2013 until 2020. The syntax TITLE-ABSTR-KEY (title-abstract-keyword) was used in combination with keywords like ‘*Euphorbia’*, OR ‘genus’ OR ‘Euphorbiaceae’, OR ‘diterpene compounds’ OR ‘*Euphorbia* diterpenes’ OR ‘macrocyclic diterpenes’, OR ‘tigliane’ OR ‘jatrophane’ OR ‘lathyrane’, OR ‘abietane’ OR ‘kaurane’ OR ‘atisane’ OR ‘biological studies’ OR ‘structure–activity relationship’ OR ‘anticancer activity’ OR ‘antibacterial’, OR ‘anti-inflammatory’. The search terms were run separately or as limited combinations depending on the database used. In addition, a plant-list database was used to ascertain the correct names of the species. The search strategy was limited to English-language publications and excluded research articles still under consideration for publication or not yet available in the databases. The search was restricted to between 2013 and 2020. The retrieved information was checked, critically read, and searched for descriptions of previously undescribed diterpenes, occurrence, structures, the biological activities, biosynthetic pathway, and the structure–activity relationships. Additional information was obtained by reviewing the listed references in the selected articles. 

## 3. Occurrence of *Euphorbia* Diterpenes

The isoprenoid constituents of *Euphorbia* species are very diverse. Over the last decade, phytochemical investigations of the roots, stems, stem barks, aerial, seeds, and whole plant extracts of *Euphorbia* species led to the isolation and structural identification of a wide range of diterpenoids. Within this time frame, over 350 (**1**–**382**) newly isolated diterpenoids, possessing different skeletal frameworks, were reported. At the same time, the compounds showed significant pharmacological activities. Over thirty *Euphorbia* species presented in this review were reported to contain these diterpenes. Furthermore, diterpenoids bearing new skeletal structures were described, and their biosynthesis was proposed. The newly reported diterpenoids structures led to new information about their biological activities and their biogenesis in plants. Plants newly investigated within this time frame were *E. kopetdaghi* [25], *E. sanctae-catharinae* [26], *E. gaditana* [27], *E. saudiarabica* [28], and *E. glomerulans* [29]. 

*Euphorbia* diterpenoids described for the first time possessed the parent skeletal structures, only differing by the type of substituent attached to the parent framework. Other diterpenoids, previously not described and classified in the genus, were isolated, such as meroterpenoids [30]. The structural diversity of the isolated diterpenoids and their analogs further enabled the studies of the structure–activity relationship to be conducted. From the findings, it was established that the existence of the hydroxyl group in some diterpenoids was essential for the activity, as it had both positive and negative effects. Esterification of the hydroxyl functionality in some of the diterpenes analogs was found to enhance their efficacy activities. These studies are important as they give vital information on pharmacophoric elements of diterpenes as promising lead compounds for drug discovery. It is also noteworthy that most of the diterpenoids were isolated from methanol and ethanol extracts of the roots, stems, aerial, stem barks, seeds, and in some cases the whole plant materials, of less than fifty species of the over 2000 species of *Euphorbia* species.

Most of the studied species contained both polycyclic and macrocyclic diterpenes and included *E. lathyris* [31], *E. stracheyi* [32], *E. royleana* [33], *E. antiquorum* [34], *E. kansuensis* [35,36], *E. prolifera* [37], *E. peplus* [38], *E. aellenii* [39], *E. pilosa* [40], *E. saudiarabica* [28], *E. marginata* [41], *E. neriifolia* [42], *E. resinifera* [43], *E. pekinensis* [44], *E. hylonoma* [45], *E. milii* [46], *E. wallichii* [47], and *E. ebracteolata* [48]. While others contained only macrocyclic diterpenes like *E. esula* [49], *E. helioscopia* [50], *E. yinshanica* [51], *E. grandicornis* [52], and *E. kansui* [53]. Of this, *E. esula* [49] had the highest number of isolated diterpenes (*n* = 44), of which the majority were jatrophane and lathyrane diterpenes, followed by *E. royleana* [33] (*n* = 36). Others were *E. neriifolia* [42], (*n* = 32), *E. lathyris* (*n* = 21) [31] (*n* = 28), *E. kansui* [53] (*n* = 23), *E. stracheyi* [32] (*n* = 22), *E. peplus* [38] (*n* = 22), *E. antiquorum* [34], (*n* = 21) and *E. marginata* [41] (*n* = 20). While other species reported only one or two diterpenes such as *E. gaditana* [27] (*n* = 1), *E. kopetdaghi* [25] (*n* = 2), *E. aellenii* [39] (*n* = 2), *E. pilosa* (*n* = 2) and *E. grandicornis* (*n* = 2) (Table 1). Anticancer, chemoreversal abilities, and anti-inflammatory activities were the most-studied biological studies.

## 4. Higher Diterpenes

**Figure 1:**
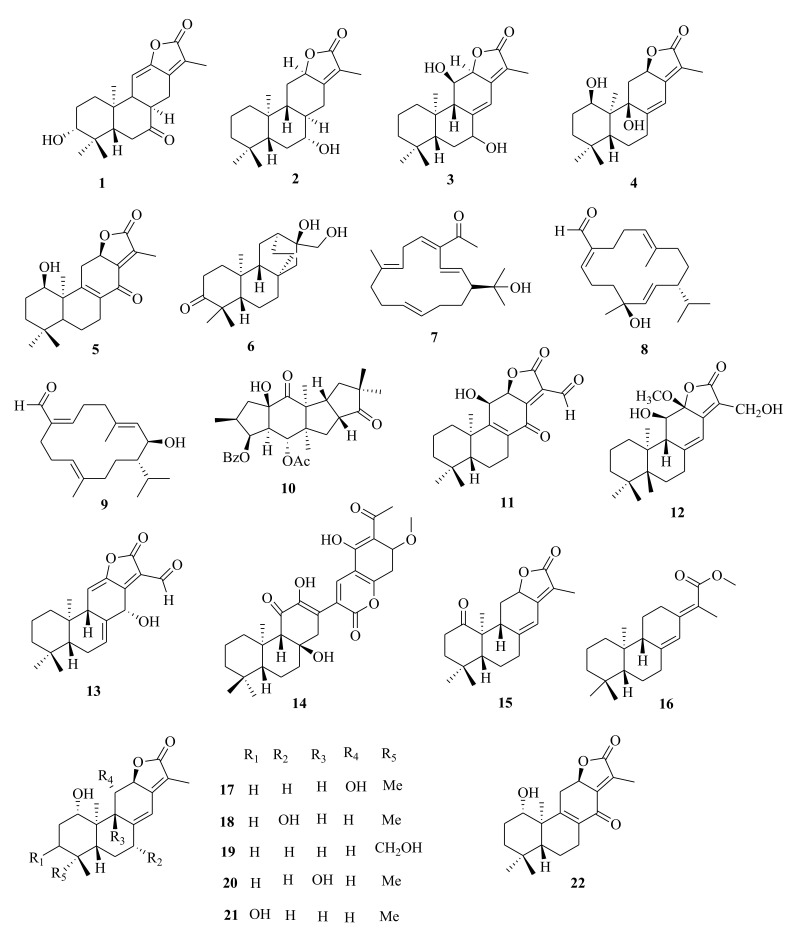
Chemical structures of abietane and atisane diterpenoids.

**Figure 2:**
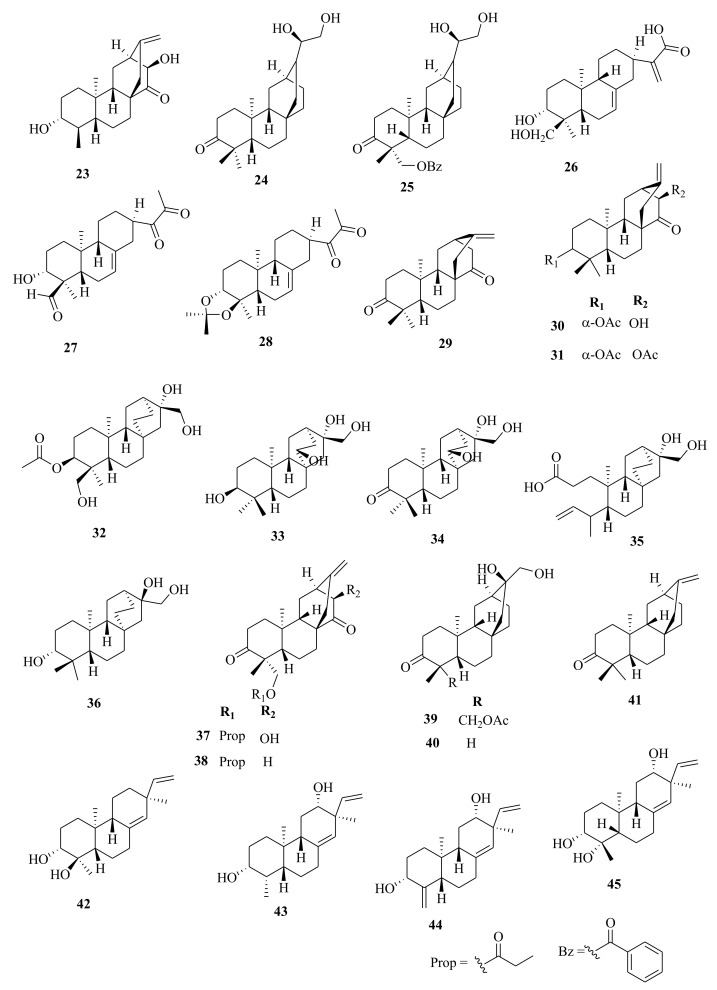
Chemical structures of cembrane and abietane diterpenoids.

**Figure 3:**
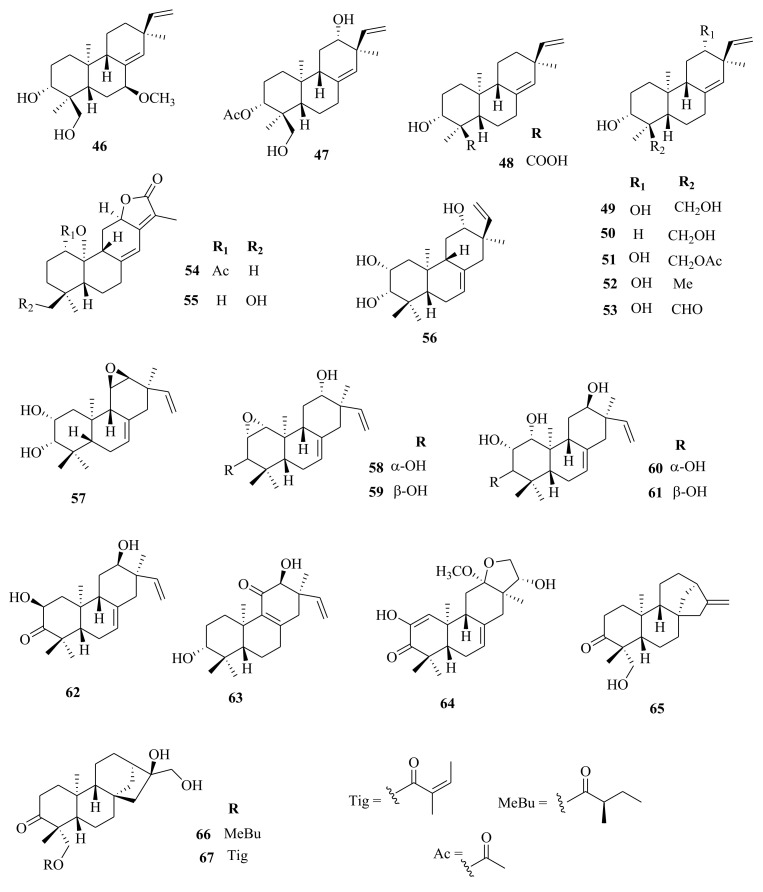
Chemical structures of *ent*-isopimarane and *ent*-kaurane diterpenoids.

**Figure 4:**
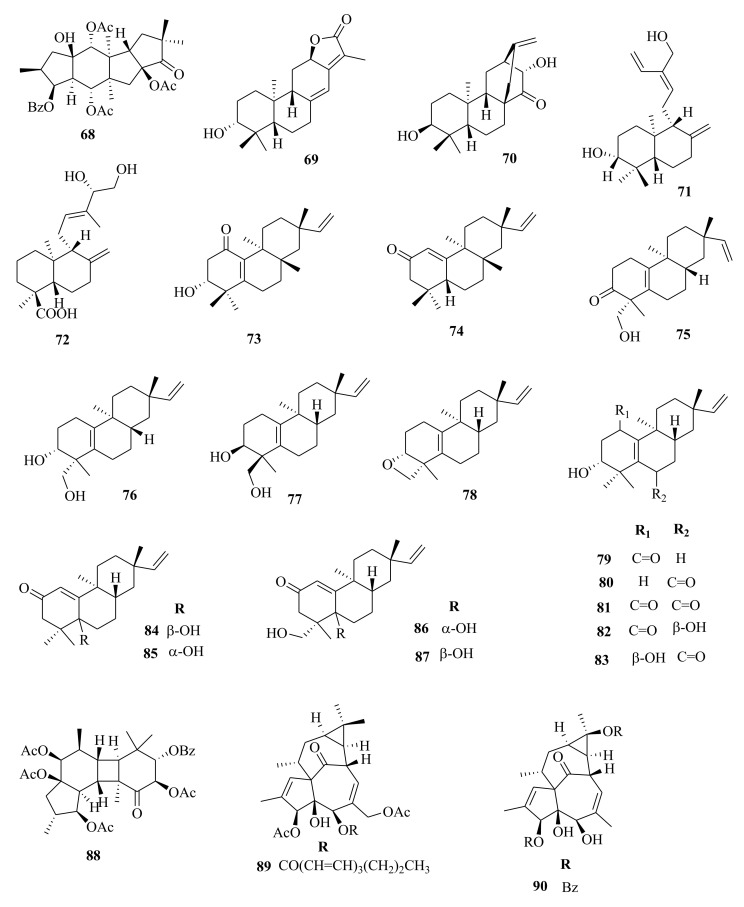
Chemical structures of ent-labdane, ent-rosane and gaditanone diterpenoids.

**Figure 5:**
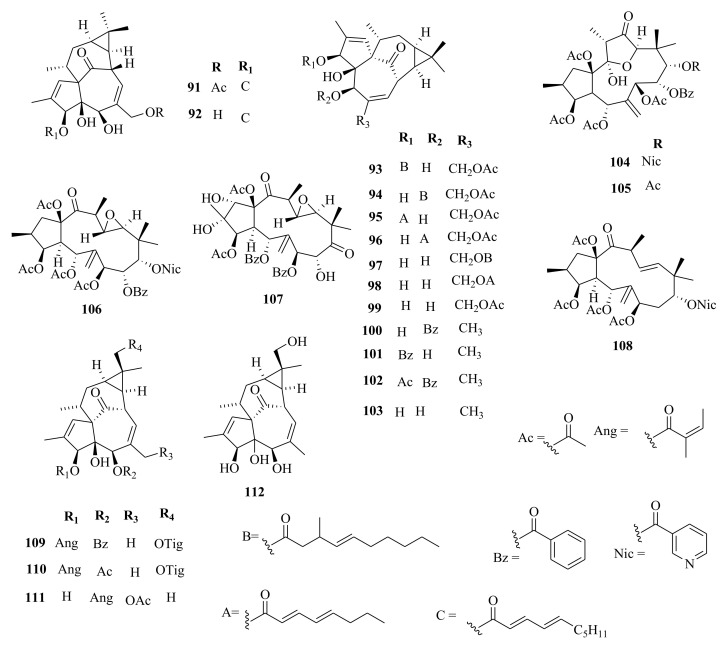
Structures of *Euphorbia* ingenane diterpenoids.

**Figure 6:**
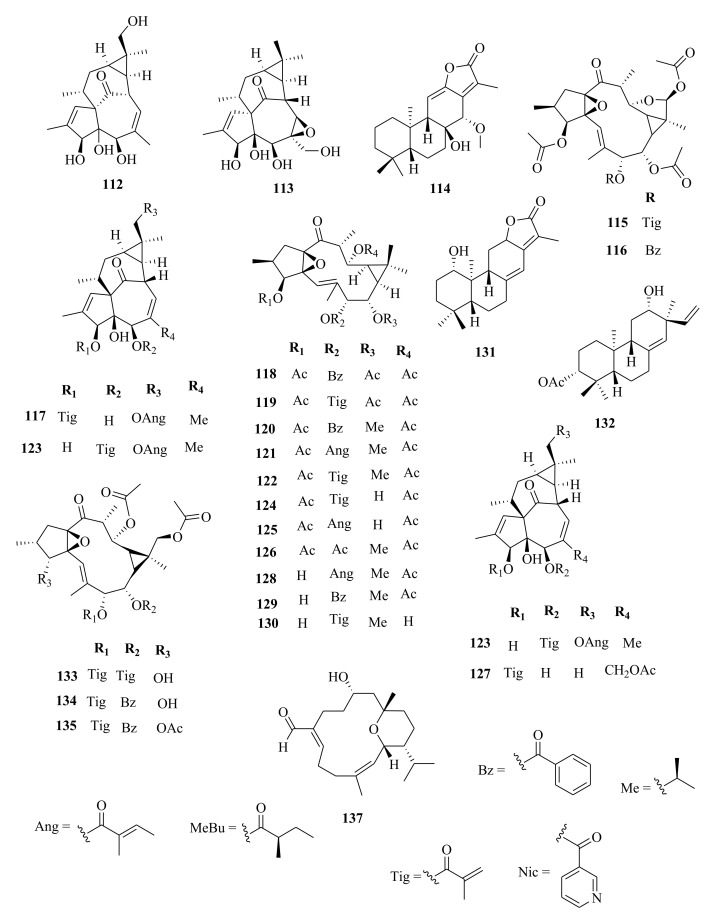
Chemical structures of *Euphorbia* ingol and ingenol diterpenoids.

**Figure 7:**
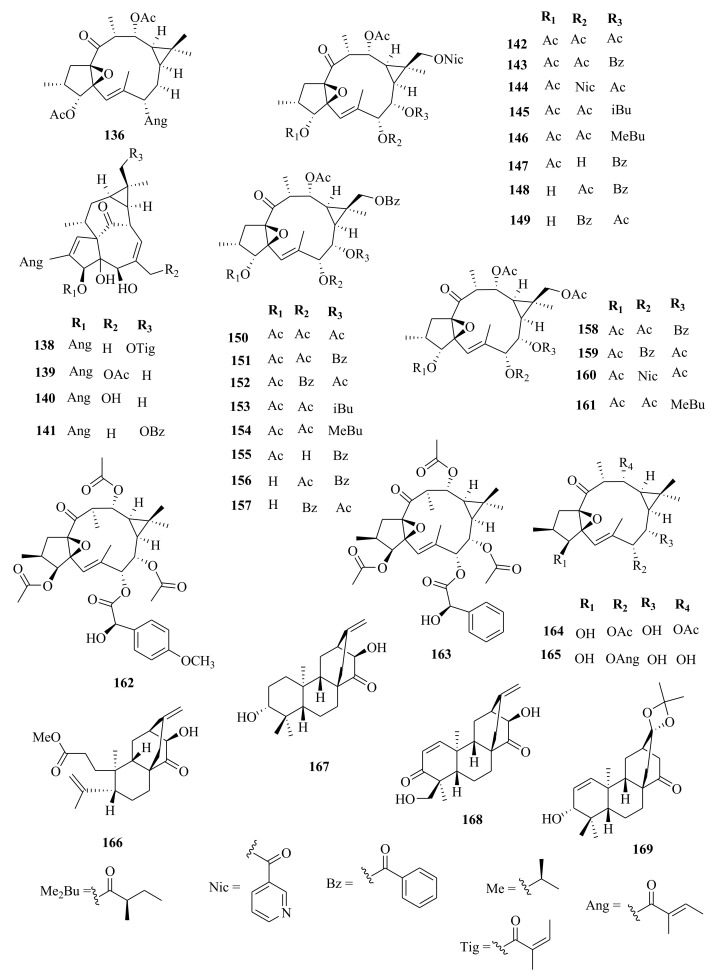
Continued: Chemical structures of ingol and ingenol diterpenoids.

**Figure 8:**
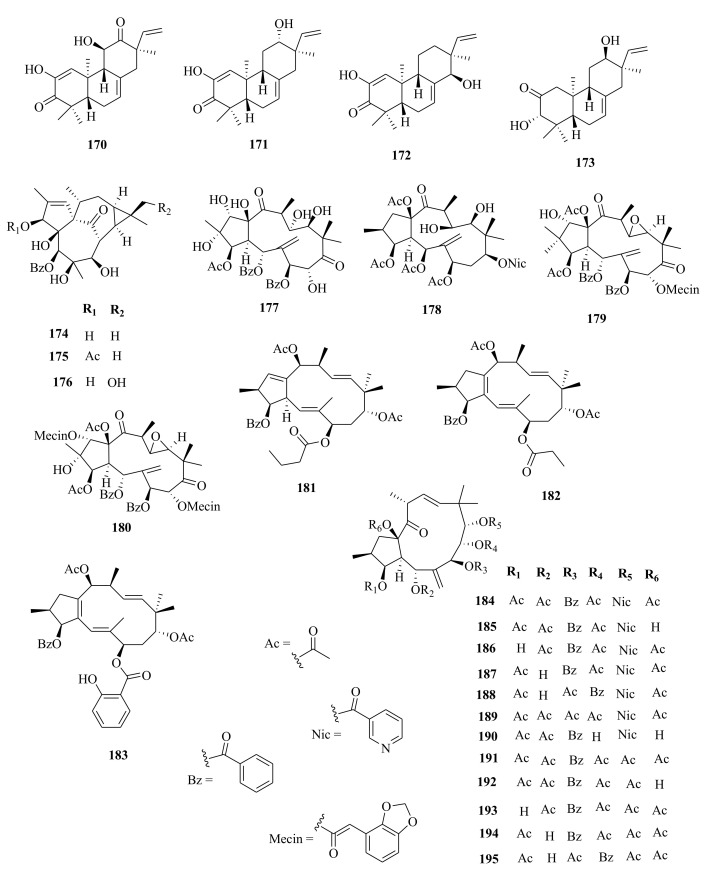
Chemical structures of isopimarane and some jatrophane diterpenoids.

**Figure 9:**
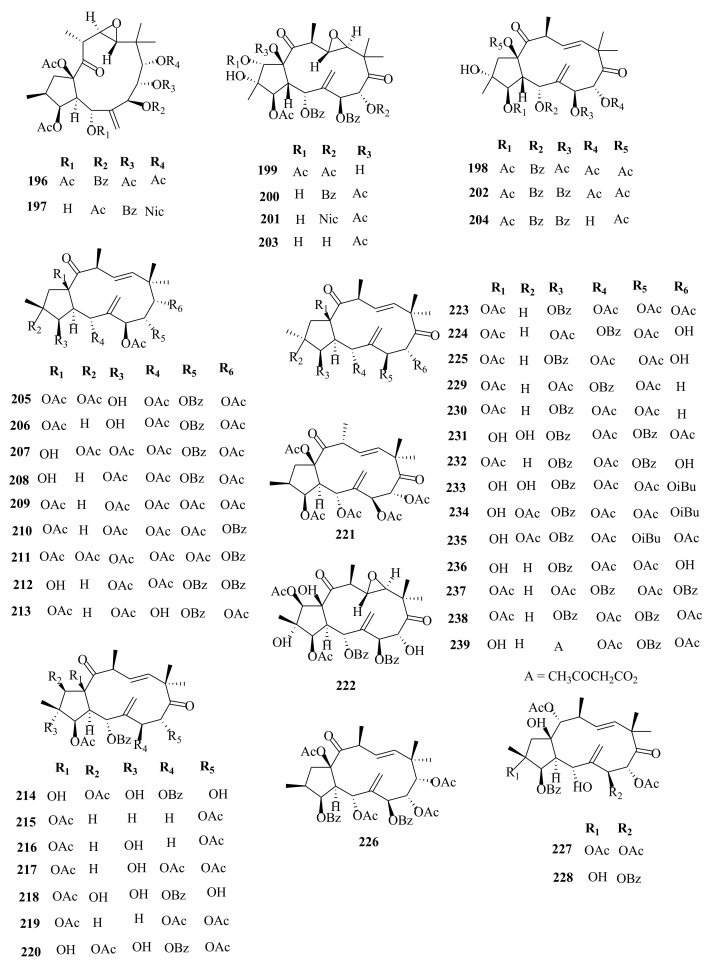
Chemical structures of *Euphorbia* jatrophane diterpenoids.

**Figure 10:**
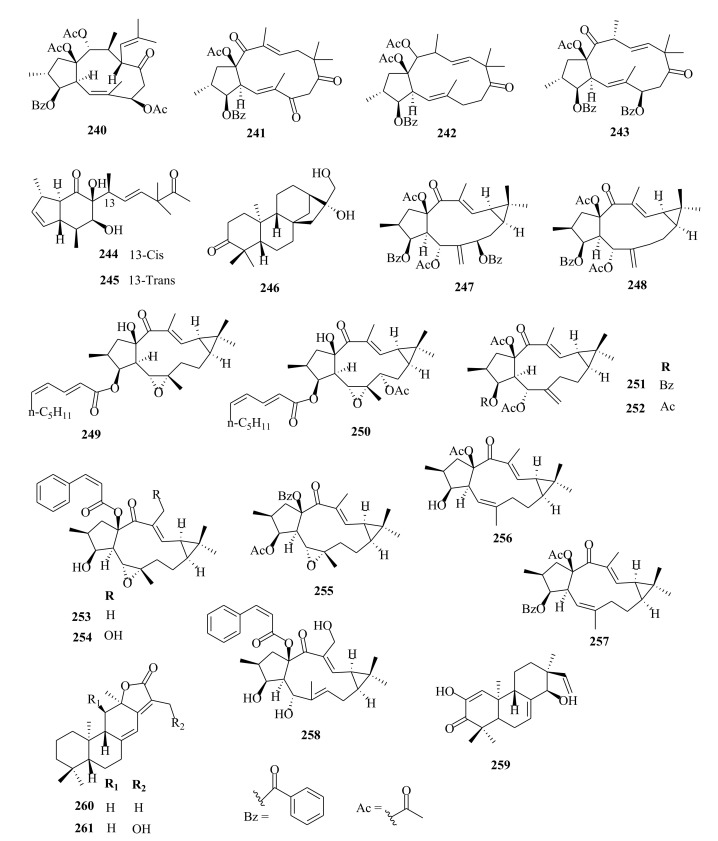
Continued: Chemical structures of *Euphorbia* jatrophane diterpenoids.

**Figure 11:**
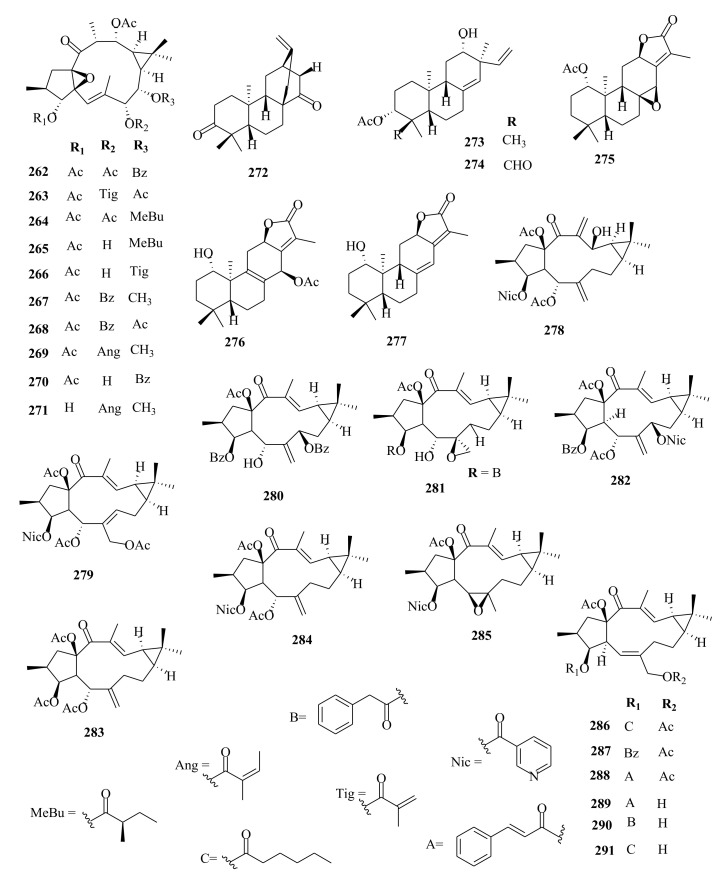
Chemical structures of *Euphorbia* kaurane and lathyrane diterpenoids.

**Figure 12:**
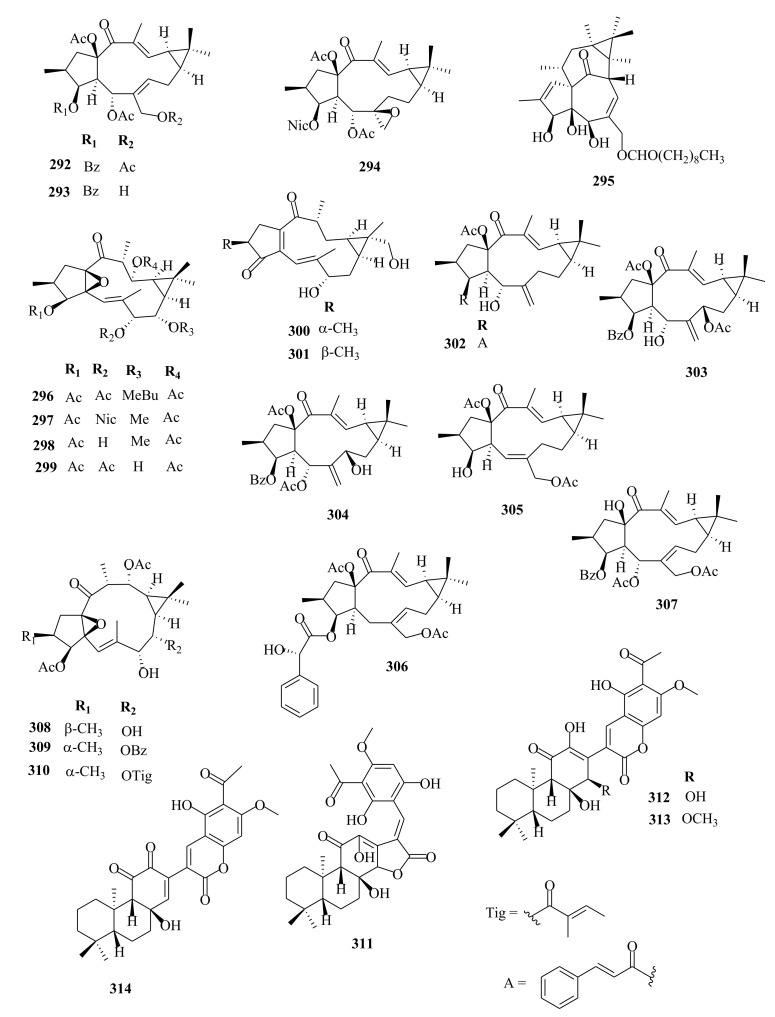
Chemical structures of *Euphorbia* lathyrane and meroterpenoid diterpenoids.

**Figure 13:**
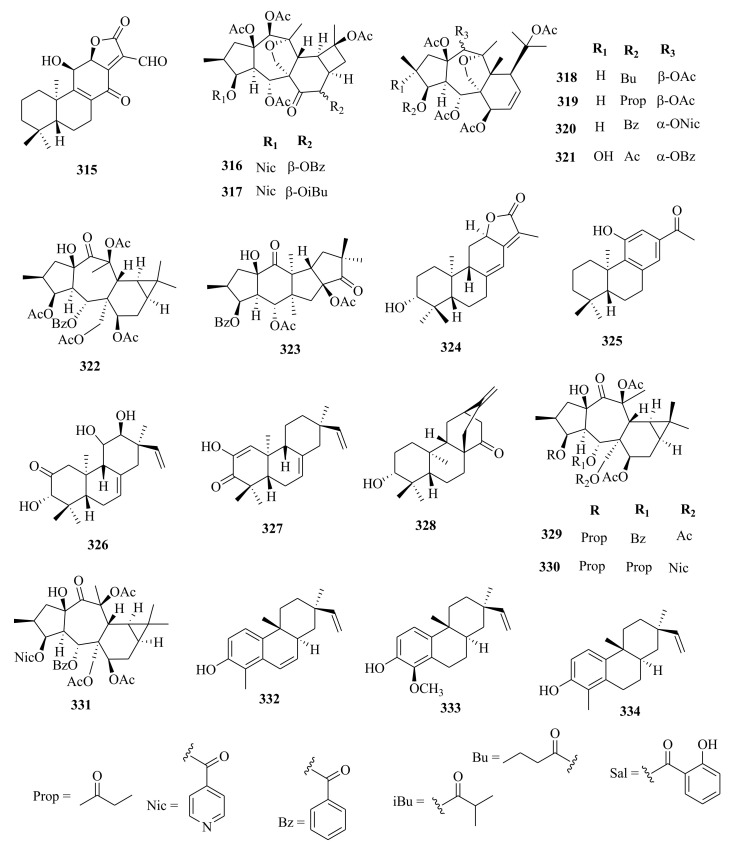
Mysrinane, premyrsinane, rosane and paralianone, pimarane, and tigliane diterpenoids.

**Figure 14:**
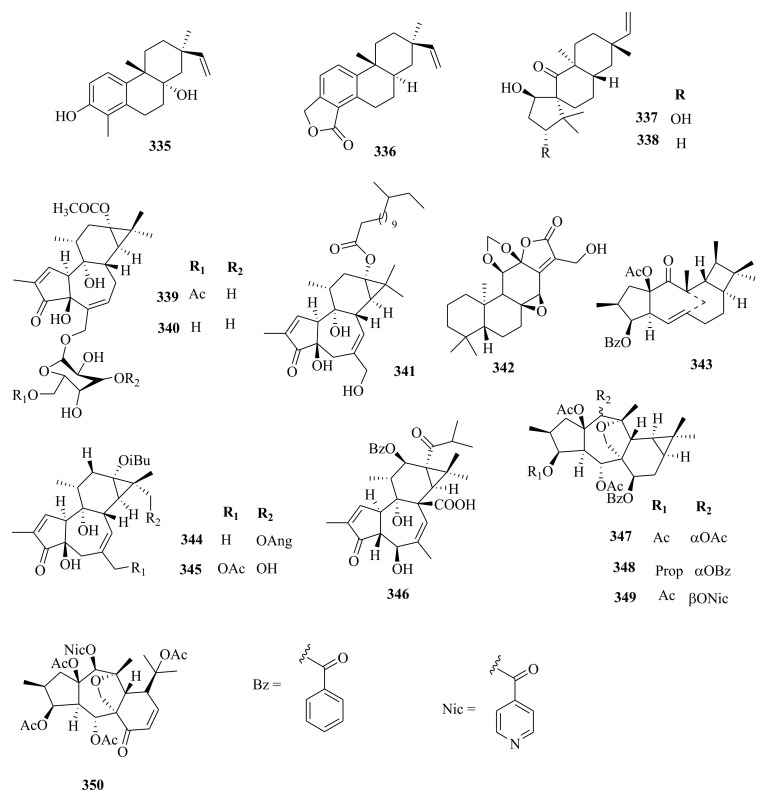
Continued: Mysrinane, premyrsinane, rosane and paralianone, pimarane, and tigliane diterpenoids.

**Figure 15:**
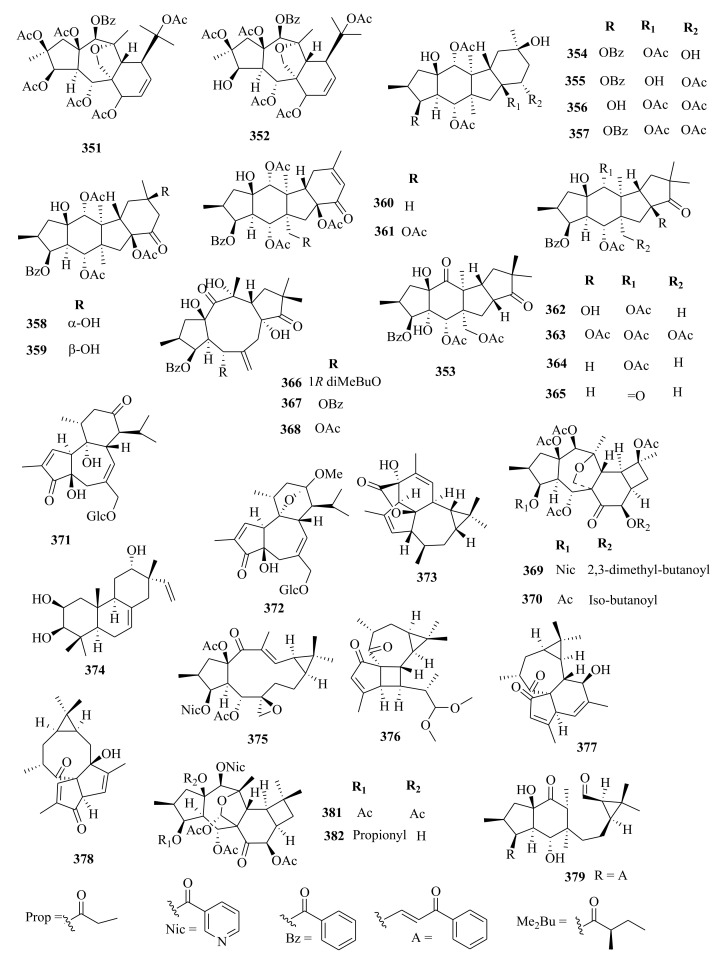
Structures of myrsinol, tigliane, paraliane, pepluane and presegetane diterpenoids.

*Euphorbia* diterpenes are classified as higher diterpenes and lower diterpenes [9,13]. Higher diterpenes are not specific to the Euphorbiacaeae family, as they occur in many other plant families as well [76]. The skeletal structures of these diterpenes involve the cyclization of the precursor to yield different cyclized diterpenes including bicyclic labdanes, clerodanes, tricyclic abietanes and tetracyclic kauranes, atisanes, and bayeranes [14,17,18,19]. In this review we have collected information about the occurrence, isolation, structure and biological activities of *Euphorbia* diterpenes between the years 2013 and 2020, as summarized in Table 2 alongside Figure 1, Figure 2, Figure 3, Figure 4, Figure 5, Figure 6, Figure 7, Figure 8, Figure 9, Figure 10, Figure 11, Figure 12, Figure 13, Figure 14 and Figure 15.

### 4.1. Abietanes, Atisane, Cembranes, Ent-Abietanes, Ent-Labdanes and Ent-Isopimaranes

Within the review period, over 200 different polycyclic diterpenes were described, including abietanes (**1**–**5**) [38,68], atisane (**6**) [35], cembranes (**7**–**9**) [33,44]; *ent*-abietanes (**10**–**28**) [34,38,47,59], *ent*-atisanes (**29**–**41**) [32,33], *ent*-labdanes (**68**–**72**) [38,51], *ent*-isopimaranes (**42**–**64**) [43,45,69,70], *ent*-kauranes (**65**–**67**) [33], *ent*-rosanes (**73**–**87**) [45,46,71], gaditanone (**88**) [27], and kaurane (**247**) [31,66]. Atisanes and cembranes were the least dominant subclasses, as only one new atisane, atisane-3-oxo-16*α*,17-diol (**6**) [35], from *E. kansuensis* and three cembranes diterpenes, euphopane C (**7**) from *E. pekinensis* [44], euphoroylean A (**8**), and B (**9**) from *E. royleana* [33], were isolated. Previous reviews between 1998 and 2008 [9] and between 2008 and 2012 [9] reported that atisanes and cembranes were the least-dominant class of diterpenes in *Euphorbia* species. Notably, these diterpenes are dominant within other genera of the Euphorbiacaeae family. For instance, *ent*-kauranes are the most dominant diterpenes in the *Isodon* genus [77].

Diterpenoids possessing rare or unusual skeletal structures were reported in *Euphorbia* species. For instance, chemical analysis of ethanol extracts of *E*. *ebracteolata* resulted in the isolation of ebraphenol A–D (**332**–**335**) [48] alongside ebralactone A (**336**), which were found to have a rosane skeletal structure with an uncommon aromatic C ring [48]. The previous unreported diterpenoids, considered as 18 (or 19)-norditerpenoids of the *ent*-isopimaranes skeleton, were isolated for the first time from *E. neriifolia* [73]. Furthermore, an unusual tetracyclic diterpenoid named eupholathone (**343**) [66] was isolated from *E. lathyris* and described for the first time in the genus. Besides, previously undescribed euphnerin A (**337**) and euphnerin B (**338**) [71] isolated from the stems of *E. neriifolia* were found to possess a spirocyclic carbon skeleton rarely found among rosane diterpenoids. This was proposed to be occasioned by rearrangement reactions. Interestingly, euphominoid E (**73**) [71], isolated from the same species, was found to co-occur with euphnerin A (**337**) [71] and euphnerin B (**338**) [71] as they had the same B/C ring systems. Based on the observations, euphominoid E (**73**) was postulated to be the precursor for the biosynthesis of euphnerin A (**337**) and euphnerin B (**338**) [71].

### 4.2. Abietane and Ent-Abietanes

Euphorbiaceae abietane and *ent*-abietanes diterpenoids usually contain an α, β-unsaturated γ-lactone ring that is connected at C-12 and C-13. Some carbons of abietane diterpenoids, like C-8, C-14, C-11, and C-12, form a double bond or can be substituted with hydroxyl or keto groups [78]. The occurrence of *ent-*abietanes diterpenoids in higher plants of the genus *Euphorbia* is rare. Previously, Satti et al. [79] reported the isolation of *ent*-abietanes diterpenoid from *E. fidjiana* and Lal et al. [80] reported the isolation of 17-hydroxyjolkinolide A, ent-11α-hydroxyabieta-8(14),13(15)-dien-16,12α-olide, ent-16-Hydroxy-13[*R*]-pimar-8(14)-ene-3,15-dione and ent-l2α,16-dihydroxy-13[*R*]-pimar-8(14)-ene-3,15-dione from *E. fidjiana*. Few other *Euphorbia* species investigated within the review period were reported to contain *ent*-abietane diterpenoids. Three previously undescribed abietane-type diterpenes; 11,12-didehydro-8*α*,14-dihydro-7-oxo-helioscopinolide A (**1**) and 8*β*-acetyl-paralianone D (**2**) isolated from the acetone extracts of *E. peplus* [38] differed from retusolides A-F previously isolated from *E. retusa* [81] and ent-abietane diterpenoids from the roots of *E. guyoniana* [82], which had a cyclopropane at C-4. The compound: 11,12-didehydro-8*α*,14-dihydro-7-oxo-helioscopinolide A (**1**) differed from helioscopinolides and secohelioscopinolides previously isolated from *E. formosana* only by the hydroxyl group at C-7 [83]. Li et al. [47] isolated ent-abietane type diterpenes (**11**–**13**) from roots extracts of *E. wallichii*. Others included euphoractone (**14**) from *E. fischeriana* [60], euphonoid F (**15**) from *E. antiquorum* [34], euphopane B (**16**) from *E. pekinensis* [44], eupneria A–I (**17**–**25**) from *E. neriifolia* [61] and fischerianoids A–C (**26**–**28**) from *E.* *fischeriana* [61]. 

### 4.3. Meroterpenoids

Meroterpenoids are frequently isolated from marine organisms and fungi. Plants are also known to produce meroterpenoids, like tetrahydrocannabinol and bakuchiol that co-occur with other types of diterpenes. Meroterpenoids occur with huge structural diversity [84]. For instance, meroterpenoids including fischernolide A–D (**311**–**314**) [30] and fischeriana A (**315**) [30] were isolated from *E.* *fischeriana* for the first time in this species. Two abietane-type diterpenoids 1*α*,9*β*-dihydroxy-ent-abieta-8(14),13(15)-dien-16,12-olide (**4**) and 1*α*-hydroxy-14-oxo-ent-abieta-8,13(15)-dien-16,12-olide (**5**) together with *ent*-abietane-type (**17**–**26**), ingol-type (**165**–**169**) and rosane-type (**337**–**338**) diterpenenoids were isolated from *E.* *neriifolia* [68]. A previous study by Baloch et al. [85] reported the isolation of ten ingenane-type diterpenes from the latex of *E. cauducifolia* (Syn. *E.* *neriifolia*). The diterpenes were substituted with either an angeloyl, acetyl, palmitoyl, benzoyl, or tetradecatrienoyl groups. Atisane derivatives and 3,4-seco-atisane-type diterpenes were reported from the stems of *E.* *neriifolia* [86]. 

### 4.4. Ent-Atisanes, Ent-Isopimaranes, Cembranes and Labdanes

*Ent*-atisanes are common diterpenoids in *Euphorbia* and other genera of higher plants. *Ent*-atisanes have a carbocyclic skeleton with a tetracyclic skeleton, comprising a perhydrophenanthrene substituent (rings A, B, and C) that is bonded to a cyclohexane unit (ring D), and have methyl groups at either C4, C10 or C16. The *ent*-atisane diterpenoids exhibit various oxidation and functionalization patterns, making a diverse class of natural products. Satti et al. [87] reported the isolation of *ent*-atisane-3β,16α,17-triol from the rhizomes of the tuber *E. acaulis*, and later Mbwambo et al. [88] isolated it from the evergreen succulent of *E. quinquecostata*. Since then, other *ent*-atisanes diterpenoids were isolated from other species within the *Euphorbia* genus from different species [32,56]. Drummond et al. [89] reviewed the isolation, structure, and bioactivity of various ent-atisanes diterpenoids from different genera, including *Euphorbia* discovered between 1965 and 2020. Ye et al. [32] isolated new diterpenenoids; *ent*-11*β*-hydroxyabieta-8(14),13(15)-dien-16,12-olide (**3**) (abietane), *ent*-atis-16-ene-3,14-dione (**29**) (*ent*-atisane) in addition to ingenane type diterpenes; *3β*, 20-diacetoxy-5*β*-deca-2′′*E*, 4′′*E*, 6′′*E*-trien-4*β*-hydroxyl-1-one (**89**), ingenane (**90**), 20-*O*-acetyl-[3-*O*-(2′*E*, 4′*Z*)-decadienoyl]-ingenol (**90**) and lathyranes (**249**–**261**) from the roots extracts of *E. stracheyi.* [32]. Yan et al. [35] described the isolation of atisane-type diterpenoid; atisane-3-oxo-16*α*,17-diol (**6**) from ethanol root extracts of *E. kansuensis*. Lathyranes-type diterpenoids were isolated from the ethanol root extracts of *E. kansuensis* by Wang et al. [90]. Shaker et al. [33] described the isolation of new *ent*-atisane diterpenoids, euphoroylean F (**30**) and euphoroylean G (**31**), from the whole-plant extract of *E. royleana.* Tran et al. [54] investigated stem extracts of *E. antiquorum* and reported the isolation of *ent*-atisanes diterpenoids (**32-35**). An et al. [56] reported the isolation of euphorin A (**37**), euphorin B (**38**) and *ent*-(3*α*,5*β*,8*α*,9*β*,10*α*,12*α*)-3-hydroxyatis-16-en-14-one (**41**) from stem extracts of *E.* *antiquorum.* In addition, Wang et al. [75] isolated (4*R*,5*S*,8*S*,9*R*,10*S*,12*S*,16*S*)-ent-19-acetoyloxy-16*α*,17-dihydroxyatisan-3-one (**39**) from stem extracts of *E. royleana.* Other diterpenes isolated from *E. royleana* include *ent*-isopimaranes (**54**–**55**) [75], *ent*-kauranes (**65**–**67**) [33], ingenanes (**109**–**111**), ingols (**136**–**141**) [33] and lathyranes (**262**–**295**) [33,75]. The findings suggest that *E. royleana* is rich in both polycyclic and macrocyclic diterpenes. Previously, Li et al. [91] investigated the aerial extracts of *E. royleana* and isolated ten ingols of 4,15-epoxylathyrane-type diterpenes.

Yan et al. [44] investigated the root extracts of *E. pekinensis* and isolated cembranes-type diterpenoids; euphopane C (**7**) and *ent*-abietane-type diterpenoids; euphopane B (**16**) and isopimarane-type (**170**–**173**) diterpenoids. Liang et al. [92] reported the isolation of casbane-type diterpenoid, pekinenal. Cembrane-type diterpenoids euphoroylean A (**8**) and euphoroylean B (**9**), isolated from the whole-plant extract of *E. royleana* [33], differed with euphopane C (**7**) only by the position of the hydroxyl group on the parent structure.

Li et al. [73] reported the isolation of *ent*-isopimaranes-type diterpenoids; eupneria J- (**42**), eupneria K (**43**), eupneria L (**44**), eupneria K (**45**), eupneria M–P (**46**–**48**), eurifoloid I (**49**), oryzalexin F (**50**), eurifoloid H (**51**), *ent*-isopimara-8(**14**),15-dien-3*β*,12*β*-diol (**52**) and 3*α*,12*α*-dihydroxy-*ent*-8(14),15-isopimaradien-18-al (**53**) from the stems barks of *E. neriifolia.* Earlier studies by Liu et al. [86] described the isolation of atisanes derivatives and 3,4-seco-atisane-type diterpenes from this species. Ent-isopimaranes-type diterpenoids were also isolated from *E. quinquecostata* [93], *E. fischeriana*, *E. characias* [94]. Wang et al. [75] reported the isolation of *ent*-isopimaranes (**54**–**55**) from stem extracts of *E. royleana*, while Wei et al. [45] isolated *ent*-isopimaranes (**56**–**64**) from roots extracts of *E. hylonoma.* Studies on *E. royleana* led to the isolation of ingenanes diterpenes euphoroylean C, D, and E (**109**–**11**), ingol-type diterpenes (**136**–**141**) [33], and lathyranes diterpenoids (**262**–**295**) [75]. Only chemoreversal, anticancer, and anti-inflammatory studies have been conducted on the isolated compounds. The species *E. royleana* is therefore a rich source of bioactive compounds that need to be exploited further. 

Labdanes and *ent*-labdanes diterpenoids helioscopinolide A (**68**) and B (**69**) were isolated from whole plants extracts of *E. peplus* [38] and *E. kansuensis* [35], respectively, while *ent-3α*,16-dihydroxylabda-8(17),12*(E)*,14-triene (**70**) and *ent*-14(S),15-dihydroxylabda-8(17)-12(*E*)-dien-18-oic acid (**71**) were isolated from *E. yinshanica* [51]. Labdanes are rare in *Euphorbia* species but are common in other higher plants and marine organisms [95]. Various reports have demonstrated the potential of naturally occurring labdanes diterpenoids as NF-κB, nitric oxide (NO), or arachidonic acid (AA) modulators as summarized in a review article published by Tran et al. [95]. Chemical investigation of *E. neriifolia* led to the isolation of *ent*-rosane diterpenoids, euphominoid E (**73**) [71], while studies on *E. hylonoma* afforded ent-rosa-1(10), 15-dien-2-one (**74**) [45]. Others included euphominoid A–L (**75**–**87**) isolated from *E. milii* [46]. Rosane diterpenoids, such as 18-hydroxyhugorosenone, were previously isolated from *E. ebracteolata.* Li et al. [48] reported the isolation of rosane diterpenoids; ebraphenol A–D (**332-335**) and ebralactone A (**336**) for the first time from the root extracts of *E. ebracteolate,* while Du et al. [71] isolated euphnerin A (**337**) and B (**338**) from stem extracts of *E. neriifolia.*

Within the wider Euphorbiacaeae family, other genera such as *Excoecaria*, *Sapium*, *Isodon*, *Xylopia*, and *Spiracea* are important sources of these diterpenes [89]. It is noted that the oxidation patterns observed of the isolated *ent-*atisanes varied distinctively with producing genus. This suggests the non-uniform expression of enzymes responsible for their biogenetic pathway across the genera. For instance, all the *ent*-atisane diterpenes derived from *Elaeoselium* and *Isodon* genera have oxidation patterns at C-16 and C-17. In contrast, *Xyopia ent*-atisanes diterpenes possess a C-16 tert hydroxyl groups while *Euphorbia* and *Sapium ent*-atisanes have unsaturated C-12. Besides, all reported 3,4-seco atisanes were isolated from *Euphorbia*, *Excoecaria*, *Croton*, and *Sapium* genera of the Euphorbiacaeae family [89]. Furthermore, daphane diterpenes were reported in Euphorbiacaeae and Thymelaeceae families [14]. Ent-kauranes and labdane diterpenes were reported in *Rabdosia* (Lamiaceae) [96], while abietanes were isolated from *Toxodium* (Toxodiaceae) species [97].

## 5. Lower Diterpenes

Lower diterpenes are macrocyclic diterpenes and their cyclized products. They are derived from a geranylgeranyl pyrophosphate precursor in a ‘head-to-tail cyclization [19,98]. The different functionalization of these diterpenes proceeds via cyclization. Macrocyclic diterpenes are characteristic compounds of the Euphorbiaceae and Thymelaeaceae families and are used as chemotaxonomic markers. In this study both macrocyclic and polycyclic diterpenes (**1**–**382**, Table 2) were reported within the review period. 

### 5.1. Ingenanes

Ingenane-type diterpenoids are characterized by a tetracyclic ring system of 5/7/7/3-and having a ketone bridge between C-8 and C-10 in addition to β-hydroxylated at C-4. The rings A and B are usually trans-fused and have a double bond between C-1 and C-2 in ring A, and between C-6 and C-7 in ring B. The carbons; C-3, C-5, C-13, C-17, and C-20 positions, in most cases, are oxygenated or esterified [9].

Chemical investigation of roots extracts of *E. kansui* yielded uncommon kansuingenol A–C (**174**–**176**) [65] possessing 6, 7-vicinal diol moiety, in addition to jatrophane type diterpenoids, kansuijatrophanol A (**177**) and B (**178**) [65] have the 11,12-vicinal diol moiety. The presence of 3,4-(methylenedioxy) cinnamyl moiety was reported for the first time in the species as seen in kansuijatrophanol C (**179**) and D (**180**) [65] jatrophane-type diterpenoids. Equally, from the whole plant ethanol extracts of *E. helioscopia*, euphorhelipanes A (**244**) and B (**245**) [99] diterpenoids with a 4-(5,5-dimethylheptan-2-yl)-2,7-dimethylbicyclo [4.3.0] nonane skeleton structure were isolated. These compounds are examples of unique diterpenes possessing a 5/6 fused carbon system isolated from *Euphorbia* species for the first time and are postulated to have originated from jatrophane. From the roots extracts of *E. kansuensis*, an ingenane type diterpenoid named, euphorkanlide A (**114**) [36], which had a C_24_ appendage resulting in an additional hexahydroisobenzofuran-fused 19-membered ring system, was isolated and described for the first time.

### 5.2. Jatrophanes and Modified Jatrophanes

Jatrophane and modified diterpenes occur mainly as polyesters in the Euphorbiaceae family. These macrocyclic diterpenes are based on a bicycle [10.3.0] pentadecane skeleton and without a cyclopropane ring. The structural diversity of jatrophane diterpenes is based on the number and positions of the double bonds within the ring, nature, and the number of oxygen functionalities, and the structural configuration of the core skeletal framework. The oxygen functionalities vary from hydroxyl, epoxy, keto, ether, to ester groups [9,19]. As a result, they occur as modified jatrophanes and have shown interesting pharmacological activities.

Within the review period, jatrophanes (**174**–**245**) [34,50,59,60,65] and modified jatrophanes; paraliane (**353**–**361**) [49,72,73], pepluane (**362–365**) [72], presegetane (**366**–**368**) [49] and lathyranes (**247**–**310**) [30,31,66,100], were predominantly isolated in *Euphorbia* species and represented over 50% of the total diterpenoids isolated in the genus. Ingenanes (**89**–**114**) [32,65], ingenol (**115**–**132**) [28,100], ingol (**133**–**169**) [28,33] and isopimaranes (**170**–**173**) [44] were the least dominant macrocyclic diterpenes. Jatrophane diterpenes are common in the *Jatropha* species of the Euphorbiaceae family [101]. Other subtypes of diterpenoids, such as pimaranes (**326**–**328**) [32] mysrinanes (**316**–**322**) [37], rosanes (**332**–**338**) [48,71], mysrinanes (**316**–**322**) [37], paralianones (**323**–**325**) [38], premyrsinane (**329**–**331**) [26], and tiglianes (**339**–**342**) [71,102] were isolated and identified for the first time. 

Examples of modified jatrophane diterpenes isolated from *Euphorbia* species were mysrinanes from *E. prolifera* (**316**–**322**) [37], paralianones from *E. peplus* (**323**–**325**) [38], pimaranes from *E. stracheyi* (**326**–**328**) [32], and premyrsinanes from *E. sanctae-catharinae* (**329**–**331**) [26]. Others were myrsinol from root extracts of *E. prolifera* (**347**–**350**) [74] and from the aerial extracts of *E. dracunculoides* (**351**–**352**) [58], paralianes from *E. esula* (**353**) [49] and *E. peplus* (**354**–**361**) [72] and pepluane (**362**–**365**) [72]. Jatrophane diterpenes can be polyacrylate derivatives, with the number of ester groups varying from three, as in guyonianin E, to eight, as in esulatin H. The acyl groups common to jatrophane diterpenes include benzoyl, acetyl, isobutanoyl, or nicotinoyl 2-methylbutanoyl, and propionyl, butanoyl, tigloyl, angeloyl, or cinnamoyl. Phytochemical analysis of *E. esula* extracts afforded rare euphoesulatins A–R (**184**–**201**) [100], belonging to the jatrophane class but differing only in their functional groups bearing oxygen such as hydroxyl, epoxy, carbonyl, and polyester. Hence, are categorized into exotypes or endotype conformers, depending on how the Δ^6 (17)^ double bond is oriented within the macrocycles. The compounds with the double bond outside the ring system were classified as exotypes, like euphoesulatin A-N (**184**–**197**) [100] while euphoesulatin O-R (**198**–**201**) [100] were classified as esulone B (2**02**), kansuinine B (**203**), and esulone A (**204**) [100], whose double bonds outside the ring were classified as endotypes. Euphoesulatin A-N (**184**–**197**) [100] and euphoesulatin R (**201**) [100] were found to uniquely possess nicotinoyloxy groups, further expanding the skeletal diversity of *Euphorbia* jatrophane diterpenoids isolated from *E. esula*. 

### 5.3. Lathyranes Diterpenes

Lathyranes form one of the largest tricyclic diterpenes of *Euphorbia* species with a 5/11/3- ring system. Biogenetically, the casbene carbon nucleus, and casbane were proposed to be the biogenetic precursors of lathyranes diterpenes. Lathyranes diterpenes contain an epoxy functionality at C-4 and C-15 or C-5 and C-6, in addition to a double bond between C-5 and C-6 and/or between C-12 and C-13. Rings A and B are usually trans-configured, while rings B and C are cis-configured. Lathyrane diterpenes were isolated from the seed extracts of *E. lathyris* (**247**–**248**) [31,66], root extracts of *E. stracheyi* (**249**–**261**) [32], whole-plant extracts of *E. royleana* (**262**–**295**) [33], and aerial extracts of *E. antiquorum* (**296**–**299**) [34]. Other records described the isolation of lathyrane diterpenes from *E. kansuensis* (**300**–**301**) [35], *E. lathyris* (**302**–**307**) [103], and *E.* *antiquorum* (**308**–**310**) [56]. 

### 5.4. Meroterpenoids

Meroterpenoid is related to *ent*-clerodane diterpenes but are lacking a cyclobutane ring and a 1-octen-3-ol substituent. Their proposed biosynthesis is via an intermolecular [2 + 2] cycloaddition between the cyclic side chains. From the roots extracts of *E. fischeriana*, an unreported meroterpenoid named euphoractone (**14**) [60] possessing a rare *ent*-abietane-phloroglucinol skeleton was isolated and identified. Meroterpenoids diterpenoids possessing moieties with 6/6/6 ring systems are common in fungi and marine organisms and are extremely rare in higher plants; they are known for exhibiting potent anticancer activities. Previous studies reported the isolation of meroterpenoid diterpenes from the *Isodon* genus of the Euphorbiacaeae family [77]. Fischeriana A (**315**) [62], possessing an unusual heptacyclic ring system (6/6/5/5/5/6/6), was isolated for the first time from root extracts of *E. fischeriana*. Meroterpenoids named fischernolides A–D (**311**–**314**) [30] possessing unique 28-carbon skeleton were previously isolated from the same species. The compounds represent unique meroterpenoids diterpenoids possessing an abietane skeleton with a conjugated acylphloroglucinol having *α*-furanone or *α*-pyrone ring. Fischernolide A (**311**) [30] was reported for the first time as an abietane-acylphloroglucinol product, having a rearranged 6/6/6/5 polycyclic skeleton structure. Furthermore, fischernolides B–D (**312**–**314**) [30] contained an unusual 28-carbon skeleton structure that was thought to be formed from an acylphloroglucinol and abietane moiety via the *α*-pyrone ring. Biogenetically, the rare meroterpenoids (**312**–**314**) were proposed to be biosynthesized through an unusual aldol condensation reaction, unlike the commonly known Diels–Alder cycloaddition [30].

### 5.5. Tiglianes

Contrary to other Euphorbiaceae and Thymelaeaceae diterpenes, tigliane diterpenoids have a 5/7/6/3- tetracyclic ring system containing a five-membered ring A, a seven-membered ring B, a six-membered ring C, and a cyclopropane system D. The core skeleton structure has 20 carbons, including five methylene, five methyl, and nine methine groups, and one quaternary carbon. In general, tigliane-type diterpenes contain a double bond between the C-1 and C-2 in ring A, and another double bond in their B-ring [58]. Tigliane (**339**–**346**) [52,57,66,71,102], myrsinol (**347**–**352**) **[58,74]**, paraliane (**353**–**361**) [49,72], pepluane (**362**–**365**) [72], presegetane (**366**–**368**) [49], cyclomyrsinol (**369**–**370**) **[39]** and daphnane (**371**–**373**) [40,53] were found dominant in *Euphorbia* species. 

Tiglianes (phorbol esters) are common to Euphorbiacaeae and Thymelaeceae families. Within the Euphorbiacaeae family, several genera such as *Excoecaria*, *Croton*, *Jatropha*, *Ostedes*, and *Sapium* were also reported to contain these diterpenes [89]. In the Thymelaeceae family, phorbol esters were reported in *Pseudomyrmex* and *Danae* genera [58]. 

### 5.6. Other Euphorbia Diterpenes

Phytochemical studies of aerial extracts of *E. saudiarabica* afforded five previously undescribed 19-acetoxyingols. Among them these were saudiarabicain A (**115**) and saudiarabicain B (**116**) [28], rare pentacyclic acetoxyingols, and two 2,3-diepimers named saudiarabicain C (**133**) and saudiarabicain D (**134**) [28] which were reported for the first time as 19-acetoxyingols epimers. Fei et al. [53] isolated for the first time a novel diterpenoid lactone named euphorikanin A (**373**) which had a unique 5/6/7/3-fused tetracyclic ring skeletal, from the ethanol roots extracts of *E. kansui*. In the same way, rare pepluacetal (**376**), pepluanol A (**377**), and B (**378**) [72] isolated from *E*. *peplus* had a 5/4/7/3, 5/6/7/3, and 5/5/8/3 fused-ring carbon skeletal framework, respectively. 

Chemical analysis of the *E. micractina* roots extracts yielded a previously undescribed minor diterpenoid, named secoeuphoractin (**379**) [67], which had a new carbon skeleton framework [67]. From the same species, a new diterpenoid with a unique 6/5/7/3 fused-ring skeleton structure named, euphorbactin (**380**), was isolated and described. This skeletal structure had not been previously identified [104]. Phytochemical investigations of the aerial extracts of *E. kopetdaghi* yielded previously undescribed cyclomyrsinol diterpenoids, named kopetdaghinane A (**381**) and B (**382**) [25], which were found to possess an oxidation pattern of a new tetrahydrofuran pattern having a hemiacetal group. This was the first report of cyclomyrsinol diterpenes from this species. The above accounts show, the abundance and structural diversity of novel diterpenoids from *Euphorbia* species yet to be discovered that can provide a wide range of potential lead compounds that can be harnessed by pharmaceutical companies for drug discovery. 

**Table 2:** Diterpenoids derived from *Euphorbia* species and their reported biological activities.

No	Species Name	Compound Name	Plant Part, Extraction Solvent	Pharmacological Effect (Cell Type, Reported Value and Control)	Reference
**Abietane**					
**1**	*E. peplus*	11,12-didehydro-8*α*,14-dihydro-7-oxo-helioscopinolide A	Whole plant, CH_3_OH	Cytotoxic (HL-60, A-549, SMMC-7721, MCF-7, SW480). Inactive at 40 µM. Control (pactlitaxel and cisplatin)	[38]
**2**		7α-hydroxy-8α,14-dihydro jolkinolide E	Whole plant, CH_3_OH	Cytotoxic (HL-60, A-549, SMMC-7721, MCF-7, SW480). Inactive at 40 µM. Control (pactlitaxel and cisplatin).	[38]
**3**	*E. stracheyi*	*ent*-11*β*-hydroxyabieta-8(14),13(15)-dien-16,12-olide	Roots, MeOH	Cytotoxic (HGC-27, MV4-11, BaF3 SKvo3, IC_50_ > 50.00 µM) compared to IC_50_ of 0.015, 0.53 µM, respectively of taxol, the positive control	[32]
**4**	*E.* *neriifolia*	1*α*,9*β*-dihydroxy-ent-abieta-8(14),13(15)-dien-16,12-olide	Aerial, EtOH	Antiangiogenic activity (HUVECs migration); no activity (IC_50_ > 50.00 µg/mL)	[68]
**5**		1*α*-hydroxy-14-oxo-ent-abieta-8,13(15)-dien-16,12-olide	Aerial, EtOH	Antiangiogenic activity (HUVECs migration); no activity (IC_50_ > 50.00 µg/mL)	[68]
**Atisane**					
**6**	*E. kansuensis*	atisane-3-oxo-16*α*,17-diol	Roots, EtOH	Inhibition of NO (IC_50_ > 50 µM; quercetin (IC_50_ = 10.80 µM)	[35]
**Cembrane**					
**7**	*E. pekinensis*	euphopane C	Roots, EtOH	Cytotoxic (C4-24B; C4-2B/ENZR, MDA-MB-231, IC_50_ = 32.30, 29.30 and >50 µM respectively). Doxorubicin (0.53, 1.06 and 0.78 µM respectively)	[35]
**8**	*E. royleana*	euphoroylean A	Whole plant, EtOH	Chemoreversal, combination abilities on Hep-G2/DOX; IC_50_ > 50 µM, Controls: verapamil (Vrp) (10.65 µM), tariquidar (Tar) (2.31 µM)	[33]
**9**		euphoroylean B	Whole plant, EtOH	Chemoreversal, combination abilities on Hep-G2/DOX; IC_50_ > 50 µM, Controls: Vrp (10.65 µM), tar (2.31 µM)	[33]
***ent*-abietane**					
**10**	*E. peplus*	11-hydroxy-*ent*-abieta-8,11,13-trien-15-one	Whole plant, CH_3_OH	Cytotoxic (HL-60, A-549, SMMC-7721, MCF-7, SW480). Inactive at 40 µM, using pactlitaxel and cisplatin as control.	[38]
**11**	*E. wallichii*	11*β*-hydroxy-14-oxo-17-al-ent-abieta-8(9), 13(15)-dien-16,12*β*-olide	Roots, EtOH	Antibacterial (T25-17; MIC = 37.00 µg/L, C159-6; MIC = 45.00 µg/L, 8152; MIC = 56.00 µg/L) using gentamicin as positive control	[47]
**12**		11*β*, 17-dihydroxy-12-methoxy-ent-abieta-8(14), 13(15)-dien-16,12A-olide	Roots, EtOH	Antibacterial (T25-17; MIC = 41.00 µg/L, C159-6; MIC = 49.00 µg/L, 8152; MIC = 60.00 µg/L) using gentamicin as positive control	[47]
**13**		14*α*-hydroxy-17-al-entabieta-7(8), 1 1(12), 13(15)-trien-16, 12-olide	Roots, EtOH	Antibacterial (T25-17; MIC = 35.00 µg/L, C159-6; MIC = 51.00 µg/L, 8152; MIC = 59.00 µg/L) using gentamicin as positive control	[47]
**14**	*E. fischeriana*	euphoractone	Roots, EtOH	Cytotoxic (H23; IC_50_ = 21.07 mmol/L, H460; IC_50_ = 20.91 mmol/L) using cisplatin the positive control	[60]
**15**	*E. antiquorum*	euphonoid F	Aerial, EtOH	Melanin synthesis (B16 cells) No activity	[34]
**16**	*E. pekinensis*	euphopane B	Roots, EtOH	Cytotoxic (C4-24B; C4-2B/ENZR, MDA-MB-231, IC_50_ = 16.90, 36.80 and > 50 µM respectively). Doxorubicin (0.53, 1.06 and 0.78 µM respectively)	[35,44]
**17**	*E. neriifolia*	eupneria A	Stem barks, C_3_H_6_O: H_2_O (7:3)	Anti-infammatory (RAW 264.7) and anti-influenza (A/WSN/33/2009 (H1N1). Inactive, using oseltamivir positive control	[69]
**18**		eupneria B	Stem barks, C_3_H_6_O: H_2_O (7:3)	Anti-infammatory (RAW 264.7) and anti-influenza (A/WSN/33/2009 (H1N1). Inactive, using oseltamivir positive control	[69]
**19**		eupneria C	Stem barks, C_3_H_6_O: H_2_O (7:3)	Anti-infammatory (RAW 264.7) and anti-influenza (A/WSN/33/2009 (H1N1). Inactive, using oseltamivir positive control	[69]
**20**		eupneria D	Stem barks, C_3_H_6_O: H_2_O (7:3)	Anti-infammatory (RAW 264.7) and anti-influenza (A/WSN/33/2009 (H1N1). Inactive, using oseltamivir positive control	[69]
**21**		eupneria E	Stem barks, C_3_H_6_O: H_2_O (7:3)	Anti-infammatory (RAW 264.7) and anti-influenza (A/WSN/33/2009 (H1N1). Inactive, using oseltamivir positive control	[69]
**22**		eupneria F	Stem barks, C_3_H_6_O: H_2_O (7:3)	Anti-infammatory (RAW 264.7) and anti-influenza (A/WSN/33/2009 (H1N1). Inactive, using oseltamivir positive control	[69]
**23**		eupneria G	Stem barks, C_3_H_6_O: H_2_O (7:3)	Anti-HIV (inactive, EC_50_ > 25 µg/mL), Cytotoxic (Hep-G2; IC_50_ = 13.70 µM; adriamycin (IC_50_ = 7.03 µM)	[70]
**24**		eupneria H.	Stem barks, C_3_H_6_O: H_2_O (7:3)	Anti-HIV (inactive, EC_50_ > 25 µg/mL), Cytotoxic (Hep-G2; IC_50_ = 13.70 µM; adriamycin (IC_50_ = 7.03 µM)	[70]
**25**		eupneria I	Stem barks, C_3_H_6_O: H_2_O (7:3)	Anti-HIV (inactive, EC_50_ > 25 µg/mL), Cytotoxic (Hep-G2; IC_50_ = 13.70 µM; adriamycin (IC_50_ = 7.03 µM)	[70]
**26**	*E.* *fischeriana*	fischerianoids A	Roots, C_3_H_6_O	Cytotoxic (HL-60; no activity, MM-231; IC_50_ = 12.10 µM, A549; no activity, SMMC-7721; IC_50_ = 32.58 µM, Hep-3B; IC_50_ = 15.95 µM), cisplatin; 1.60, 3.82, 2.81, 2.78 and 2.97 µM respectively)	[61]
**27**		fischerianoids B	Roots, C_3_H_6_O	Cytotoxic (HL-60; IC_50_ = 28.78 µM, MM-231; IC_50_ = 9.12 µM, A549; no activity, SMMC-7721; no activity, Hep-3B; IC_50_ = 8.50 µM), cisplatin; 1.60, 3.82, 2.81, 2.78 and 2.97 µM respectively)	[61]
**28**		fischerianoids C	Roots, C_3_H_6_O	Cytotoxic (HL-60; no activity, MM-231; IC_50_ = 25.45 µM, A549; no activity, SMMC-7721; no activity, Hep-3B; IC_50_ = 27.34 µM), cisplatin; 1.60, 3.82, 2.81, 2.78 and 2.97 µM respectively)	[61]
***ent*-atisane**					
**29**	*E. stracheyi*	*ent*-atis-16-ene-3,14-dione	Roots, MeOH	Cytotoxic (HGC-27, MV4-11, BaF3 SKvo3, IC_50_ > 50.00) compared to IC_50_ of 0.015, 0.53 µM, respectively of taxol, the positive control	[32]
**30**	*E. royleana*	euphoroylean F	Whole plant, EtOH	Chemoreversal, combination abilities on Hep-G2/DOX; IC_50_ > 100 (10.65 µM), tar (2.31 µM)	[33]
**31**		euphoroylean G	Whole plant, EtOH	Chemoreversal, combination abilities on Hep-G2/DOX; IC_50_ > 100 (10.65 µM), tar (2.31 µM)	[33]
**32**	*E. antiquorum*	ent-3*α*-acetoxy-16*β*,17,18-trihydroxyatisane	Stems, MeOH	Inhibitory (*α*-glucosidase); IC_50_ = 119.90 µM. Cytotoxicity (K562; no activity). Acarbose (IC_50_ = 162.50 µM)	[54]
**33**	*E. antiquorum*	ent-3*α*,14,16b,17-tetrahydroxyatisane	Stems, MeOH	Inhibitory (*α*-glucosidase); IC_50_ > 200.00 µM. Cytotoxicity (K562; no activity). Acarbose (IC_50_ = 162.50 µM)	[54]
**34**		ent-14[*S*],16*α*,17-trihydroxyatisan-3-one	Stems, MeOH	Inhibitory (*α*-glucosidase); IC_50_ = 135.50 µM. Cytotoxicity (K562; no activity). Acarbose (IC_50_ = 162.50 µM)	[54]
**35**		gallochaol C	Stems, MeOH	Inhibitory (*α*-glucosidase); IC_50_ = 134.30 µM. Cytotoxicity (K562; no activity). Acarbose (IC_50_ = 162.50 µM)	[54]
**36**	*E. kansuensis*	ent-atisane-3*β*,16*α*,17-triol	Roots, EtOH	Inhibition of NO (IC_50_ > 50 µM; quercetin (IC_50_ = 10.80 µM)	[35]
**37**	*E.* *antiquorum*	euphorin A	Stems, MeOH	Inhibitory (NO production in BV-2; IC_50_ = 35.80 µM); 2-methyl-2-thiopseudourea, sulfate (SMT) (4.2 µM)	[56]
**38**		euphorin B	Stems, MeOH	Inhibitory (NO production in BV-2; IC_50_ = 41.40 SMT (4.2 µM); SMT (4.2 SMT (4.2 µM)	[56]
**39**	*E. royleana*	(4*R*,5*S*,8*S*,9*R*,10*S*,12*S*,16*S*)-ent-19-acetoyloxy-16*α*,17-dihydroxyatisan-3-one	Stems, MeOH	Inhibitory (NO production in BV-2; IC_50_ > 50 µM); SMT (3.7 µM)	[75]
**40**		(4*R*,5*R*,8*S*,9*R*, -10*R*,12*S*,16*S*)-ent-16*α*,17-dihydroxy-19-noratisan-3-one	Stems, MeOH	Inhibitory (NO production in BV-2; IC_50_ > 50 µM); SMT (3.7 µM)	[75]
**41**	*E. antiquorum*	*ent*-(3*α*,5*β*,8*α*,9*β*,10*α*,12*α*)-3-hydroxyatis-16-en-14-one	Stems, MeOH	Inhibitory (NO production in BV-2; IC_50_ = 71.0 SMT (4.2 µM); SMT (4.2 SMT (4.2 µM)	[56]
***ent*-isopimarane**					
**42**	*E. neriifolia*	eupneria J.	Stem barks, EtOH	Anti-HIV (HIV-1 NL4-3; 0.31 μg/mL), AZT; 0.0043 μg/mL	[73]
**43**		eupneria K.	Stem barks, EtOH	Anti-HIV (HIV-1 NL4-3), inactive (IC_50_ > 25.00 µg/mL), AZT; 0.0043 μg/mL	[73]
**44**		eupneria L	Stem barks, EtOH	Anti-HIV (HIV-1 NL4-3), inactive (IC_50_ > 25.00 µg/mL), AZT; 0.0043 μg/mL	[73]
**45**		eupneria M	Stem barks, EtOH	Anti-HIV (HIV-1 NL4-3), inactive (IC_50_ > 25.00 µg/mL), AZT; 0.0043 μg/mL	[73]
**46**		eupneria N	Stem barks, EtOH	Anti-HIV (HIV-1 NL4-3), inactive (IC_50_ > 25.00 µg/mL), AZT; 0.0043 μg/mL	[73]
**47**		eupneria O	Stem barks, EtOH	Anti-HIV (HIV-1 NL4-3), inactive (IC_50_ > 25.00 µg/mL), AZT; 0.0043 μg/mL	[73]
**48**		eupneria P	Stem barks, EtOH	Anti-HIV (HIV-1 NL4-3), inactive (IC_50_ > 25.00 µg/mL), AZT; 0.0043 μg/mL	[73]
**49**		eurifoloid I	Stem barks, EtOH	Anti-HIV (HIV-1 NL4-3), inactive (IC_50_ > 25.00 µg/mL), AZT; 0.0043 μg/mL	[73]
**50**		oryzalexin F	Stem barks, EtOH	Anti-HIV (HIV-1 NL4-3), inactive (IC_50_ > 25.00 µg/mL), AZT; 0.0043 μg/mL	[73]
**51**		eurifoloid H	Stem barks, EtOH	Anti-HIV (HIV-1 NL4-3; 6.70 μg/mL), MDCK, AZT; 0.0043 μg/mL	[73]
**52**		*ent*-isopimara-8(14),15-dien-3*β*,12*β*-diol	Stem barks, EtOH	Anti-HIV (HIV-1 NL4-3), MDCK; 3.86 μg/mL. AZT; 0.0043 μg/mL	[73]
**53**		3*α*,12*α*-dihydroxy-*ent*-8(14),15-isopimaradien-18-al	Stem barks, EtOH	Anti-HIV (HIV-1 NL4-3), inactive (IC_50_ > 25.00 µg/mL), AZT; 0.0043 μg/mL	[73]
**54**	*E. royleana*	(1*S*,5*R*,9*R*,10*R*,12*R*)-1*α*-acetoyloxy-ent-abieta-8(14),13-(15)-dien-12*α*,l6-	Stems, MeOH	Inhibitory (NO production in BV-2; IC_50_ = 12.0 µM); SMT (3.7 µM)	[75]
**55**		(1*S*,4*S*,5*R*,9*R*,10*S*,12*R*)-1*α*,18-dihydroxy-ent-abieta-8(14),13(15)-dien-12*α*,l6-olide	Stems, MeOH	Inhibitory (NO production in BV-2; IC_50_ > 50 µM); SMT (3.7 µM)	[75]
**56**	*E. hylonoma*	(2*R*,3*S*,12*S*)-2,3,12-trihydroxy-ent-isopimara-7,15-diene	Roots, EtOH	Inhibitory (NO in RAW264.7; IC_50_ = 45.48 µM; indomethacin (IC_50_ = 41.41 µM)	[45]
**57**		(2*R*,3*S*,11*R*,12*S*)-2,3-dihydroxy-11,12-epoxy-ent-isopimara-7,15-diene	Roots, EtOH	Inhibitory (NO in RAW264.7; not evaluated, indomethacin (IC_50_ = 41.41 µM)	[45]
**58**		(1*R*,2*S*,3*S*,12*S*)-1,2-epoxy-3,12-dihydroxy-ent-isopimara-7,15-diene	Roots, EtOH	Inhibitory (NO in RAW264.7; not evaluated, indomethacin (IC_50_ = 41.41 µM)	[45]
**59**		(1*R*,2*S*,3*R*,12*S*)-1,2-epoxy-3,12-dihydroxy-ent-isopimara-7,15-diene	Roots, EtOH	Inhibitory (NO in RAW264.7; IC_50_ = 57.51 µM; indomethacin (IC_50_ = 41.41 µM)	[45]
**60**		(1*R*,2*S*,3*S*,12*R*)-1,2,3,12-tetrahydroxy-ent-isopimara-7,15-diene	Roots, EtOH	Inhibitory (NO in RAW264.7; not active; indomethacin (IC_50_ = 41.41 µM)	[45]
**61**		(1*R*,2*S*,3*R*,12*R*)-1,2,3,12-tetrahydroxy-ent-isopimara-7,15-diene	Roots, EtOH	Inhibitory (NO in RAW264.7; not active µM; indomethacin (IC_50_ = 41.41 µM)	[45]
**62**		(2*S*,12*R*)-2,12-dihydroxy-ent-isopimara-7,15-dien-3-one	Roots, EtOH	Inhibitory (NO in RAW264.7; not active; indomethacin (IC_50_ = 41.41 µM)	[45]
**63**		3*α*,12*β*-dihydroxy-ent-isopimara-8,15-dien-11-one	Roots, EtOH	Inhibitory (NO in RAW264.7; IC_50_ > 100 µM; indomethacin (IC_50_ = 41.41 µM)	[45]
**64**		(12*R*,13*R*,15*R*)-2,15-dihydroxy-12,16-epoxy-12-methoxy-ent-isopimara-1,7-dien-3-one	Roots, EtOH	Inhibitory (NO in RAW264.7; not active; indomethacin (IC_50_ = 41.41 µM)	[45]
***ent*-kaurane**					
**65**	*E. royleana*	euphoroylean H	Whole plant, EtOH	Chemoreversal, combination abilities on Hep-G2/DOX; IC_50_ > 100 (10.65 µM), tar (2.31 µM)	[33]
**66**		(4*R*,5*S*,8*S*,9*R*,10*S*,13*R*,16*S*)-ent-16*α*,17-dihydroxy-19-(2*β*-methylbutanoyloxy)kauran-3-one	Stems, MeOH	Inhibitory (NO production in BV-2; IC_50_ = 32.60 µM); SMT (3.7 µM)	[75]
**67**		(4*R*,5*S*,8*S*,9*R*,10*S*,13*R*,16*S*)-ent-16*α*,17-dihydroxy-19-tigloyloxykauran-3-one	Stems, MeOH	Inhibitory (NO production in BV-2; IC_50_ = 19.30 µM); SMT (3.7 µM)	[75]
***ent*-labdane**					
**68**	*E. peplus*	helioscopinolide A	Whole plant, CH_3_OH	Cytotoxic (HL-60, A-549, SMMC-7721, MCF-7, SW480). Inactive at 40 µM, using paclitaxel and cisplatin as control.	[38]
**69**	*E. kansuensis*	helioscopinolide A	Roots, EtOH	Inhibition of NO (IC_50_ = 47.0 µM; quercetin (IC_50_ = 10.80 µM)	[35]
**70**		neriifolene	Roots, EtOH	Inhibition of NO (IC_50_ > 50 µM; quercetin (IC_50_ = 10.80 µM)	[35]
**71**	*E. yinshanica*	*ent-3α*,16-dihydroxylabda-8(17),12*(E)*,14-triene	Roots, EtOH	Cytotoxic (HL-60, SMMC-7721, A-549, MCF-7, SW-480); not active (IC_50_ > 40 µM) using cisplatin control	[51]
**72**		*ent*-14(S),15-dihydroxylabda-8(17)-12(*E*)-dien-18-oic acid	Roots, EtOH	Cytotoxic (HL-60, SMMC-7721, A-549, MCF-7, SW-480); not active (IC_50_ > 40 µM) using cisplatin control	[51]
***ent*-rosane**					
**73**	*E. neriifolia*	euphominoid E	Stems, MeOH	Not evaluated	[71]
**74**	*E. hylonoma*	ent-rosa-1(10),15-dien-2-one	Roots, EtOH	Inhibitory (NO in RAW264.7; IC_50_ = 48.40 µM; Indomethacin (IC_50_ = 41.41 µM)	[45]
**75**	*E. milii*	euphominoid A	Aerial, C_3_H_6_O	Inhibitory (anti-EBV lytic replication; EC_50_ = 13.20 µM) compared to (+)-rutamarin (EC_50_ = 5.40 µM)	[46]
**76**		euphominoid B	Aerial, C_3_H_6_O	Inhibitory (anti-EBV lytic replication; EC_50_ = 5.40 µM) compared to (+)-rutamarin (EC_50_ = 5.40 µM)	[46]
**77**		euphominoid C	Aerial, C_3_H_6_O	Inhibitory (anti-EBV lytic replication; EC_50_ = 24.40 µM) compared to (+)-rutamarin (EC_50_ = 5.40 µM)	[46]
**78**		euphominoid D	Aerial, C_3_H_6_O	Inhibitory (anti-EBV lytic replication; EC_50_ > 50 µM) compared to (+)-rutamarin (EC_50_ = 5.40 µM)	[46]
**79**		euphominoid E	Aerial, C_3_H_6_O	Inhibitory (anti-EBV lytic replication; EC_50_ > 50 µM) compared to (+)-rutamarin (EC_50_ = 5.40 µM)	[46]
**80**		euphominoid F	Aerial, C_3_H_6_O	Inhibitory (anti-EBV lytic replication; EC_50_ > 50 µM) compared to (+)-rutamarin (EC_50_ = 5.40 µM)	[46]
**81**		euphominoid G	Aerial, C_3_H_6_O	Inhibitory (anti-EBV lytic replication; EC_50_ > 50 µM) compared to (+)-rutamarin (EC_50_ = 5.40 µM)	[46]
**82**		euphominoid H	Aerial, C_3_H_6_O	Inhibitory (anti-EBV lytic replication; EC_50_ > 50 µM) compared to (+)-rutamarin (EC_50_ = 5.40 µM)	[46]
**83**		euphominoid I	Aerial, C_3_H_6_O	Inhibitory (anti-EBV lytic replication; EC_50_ > 50 µM) compared to (+)-rutamarin (EC_50_ = 5.40 µM)	[46]
**84**		euphominoid J	Aerial, C_3_H_6_O	Inhibitory (anti-EBV lytic replication; EC_50_ = 29.21 µM) compared to (+)-rutamarin (EC_50_ = 5.40 µM)	[46]
**85**		5-*epi*-euphominoid J	Aerial, C_3_H_6_O	Inhibitory (anti-EBV lytic replication; EC_50_ > 50 µM) compared to (+)-rutamarin (EC_50_ = 5.40 µM)	[46]
**86**		euphominoid K	Aerial, C_3_H_6_O	Inhibitory (anti-EBV lytic replication; EC_50_ > 50 µM) compared to (+)-rutamarin (EC_50_ = 5.40 µM)	[46]
**87**		euphominoid L	Aerial, C_3_H_6_O	Inhibitory (anti-EBV lytic replication; EC_50_ > 50 µM) compared to (+)-rutamarin (EC_50_ = 5.40 µM)	[46]
**Gaditanone**					
**88**	*E. gaditana*	gaditanone	Whole plant, MeOH	Not evaluated	[27]
**Ingenane**					
**89**	*E. stracheyi*	*3β*, 20-diacetoxy-5*β*-deca-2′′*E*, 4′′*E*, 6′′*E*-trien-4*β*-hydroxyl-1-one	Roots, MeOH	Cytotoxic (HGC-27; IC_50_ = 23.76 µM; taxol (0.015 µM), MV4-11; IC_50_ = 7.92 µM; taxol (0.055 µM), BaF3; IC_50_ > 20.00 µM compared to IC_50_ of 0.015, 0.53 µM, respectively of taxol	[32]
**90**		ingenane	Roots, MeOH	Cytotoxic (HGC-27; IC_50_ = 48.81; taxol (0.015 µM), MV4-11; 7.92; taxol (0.055 µM) BaF3; IC_50_ > 20.00) compared to IC_50_ of 0.015, 0.53 µM, respectively of taxol	[32]
**91**		20-*O*-acetyl-[3-*O*-(2′*E*, 4′*Z*)-decadienoyl]-ingenol	Roots, MeOH	Cytotoxic (HGC-27; IC_50_ = 41.51; taxol (0.015 µM) MV4-11; IC_50_ = 3.18; taxol (0.055 µM) BaF3, compared to IC_50_ of 0.015, 0.53 µM, respectively of taxol	[32]
**92**		3-*O*-(2′*E*, 4′*Z*)-decadienoylingenol	Roots, MeOH	Cytotoxic (HGC-27; IC_50_ = 48.51; taxol (0.015 µM); MV4-11; IC_50_ = 10.80; taxol (0.055 µM) compared to IC_50_ of 0.015, 0.53 µM, respectively of taxol	[32]
**93**	*E. kansui*	3-*O*-(2′*E*, *4′Z*-decadienoyl)-20-*O*-acetylingenol	Roots, EtOH	Antiproliferative (MCF-7; IC_50_ > 30 µM, Hep-G2; IC_50_ > 30 µM, DU145; IC_50_ = 24.49 µM)	[65]
**94**		5-*O*-(2′*E*, 4′*Z*-decadienoyl)-20-*O*-acetylingenol	Roots, EtOH	Antiproliferative (MCF-7; IC_50_ > 30 µM, Hep-G2; IC_50_ > 30 µM, DU145; IC_50_ > 30 µM)	[65]
**95**		3-*O*-(2′*E*, 4′*E*-decadienoyl)-20-*O*-acetylingenol	Roots, EtOH	Antiproliferative (MCF-7; IC_50_ > 30 µM, Hep-G2; IC_50_ = 24.07 µM, DU145; IC_50_ = 8.20 µM)	[65]
**96**		5-*O*-(2′*E*, 4′*E*-decadienoyl)-20-*O*-acetylingenol	Roots, EtOH	Antiproliferative (MCF-7; IC_50_ = 25.76 µM, Hep-G2; IC_50_ = 26.96 µM, DU145; IC_50_ = 16.24 µM)	[65]
**97**		20-*O*-(2′*E*, 4′*Z*-decadienoyl) ingenol	Roots, EtOH	Antiproliferative (MCF-7; IC_50_ = 30.48 µM, Hep-G2; IC_50_ = 12.79 µM, DU145; IC_50_ = 8.86 µM)	[65]
**98**		20-*O*-(2′*E*, 4′*E*-decadienoyl) ingenol	Roots, EtOH	Antiproliferative (MCF-7; IC_50_ > 30 µM, Hep-G2; IC_50_ > 30 µM, DU145; IC_50_ > 30 µM)	[65]
**99**		20-*O*-acetylingenol	Roots, EtOH	Antiproliferative (MCF-7; IC_50_ > 30 µM, Hep-G2; IC_50_ > 30 µM, DU145; IC_50_ > 30 µM)	[65]
**100**		5-*O*-benzoyl-20-deoxyingenol	Roots, EtOH	Antiproliferative (MCF-7; IC_50_ = 28.35 µM, Hep-G2; IC_50_ = 24.56 µM, DU145; IC_50_ = 15.55 µM)	[65]
**101**		3-*O*-benzoyl-20-deoxyingenol	Roots, EtOH	Antiproliferative (MCF-7; IC_50_ = 25.56 µM, Hep-G2; IC_50_ = 23.75 µM, DU145; IC_50_ = 9.91 µM)	[65]
**102**		kansuiphorin C	Roots, EtOH	Antiproliferative (MCF-7; IC_50_ = 12.58 µM, Hep-G2; IC_50_ = 25.00 µM, DU145; IC_50_ = 7.38 µM)	[65]
**103**		20-deoxyingenol	Roots, EtOH	Antiproliferative (MCF-7; IC_50_ > 30 µM, Hep-G2; IC_50_ > 30 µM, DU145; IC_50_ > 30 µM)	[65]
**104**		kansuinin D	Roots, EtOH	Antiproliferative (MCF-7; IC_50_ > 30 µM, Hep-G2; IC_50_ > 30 µM, DU145; IC_50_ > 30 µM)	[65]
**105**		kansuinins A	Roots, EtOH	Antiproliferative (MCF-7; IC_50_ > 30 µM, Hep-G2; IC_50_ > 30 µM, DU145; IC_50_ > 30 µM)	[65]
**106**		kansuinin E	Roots, EtOH	Antiproliferative (MCF-7; IC_50_ > 30 µM, Hep-G2; IC_50_ > 30 µM, DU145; IC_50_ > 30 µM)	[65]
**107**		kansuinin B	Roots, EtOH	Antiproliferative (MCF-7; IC_50_ > 30 µM, Hep-G2; IC_50_ > 30 µM, DU145; IC_50_ > 30 µM)	[59,65]
**108**		3,5,7,15-tetraacetoxy-9-nicotinoyloxy- 14-oxojatropha-6(17),11-diene	Roots, EtOH	Antiproliferative (MCF-7; IC_50_ > 30 µM, Hep-G2; IC_50_ > 30 µM, DU145; IC_50_ > 30 µM)	[65]
**109**	*E. royleana*	euphoroylean C	Whole plant, EtOH	Chemoreversal, combination abilities on Hep-G2/DOX; IC_50_ > 50 (10.65 µM), tar (2.31 µM)	[33]
**110**		euphoroylean D	Whole plant, EtOH	Chemoreversal, combination abilities on Hep-G2/DOX; IC_50_ > 50 (10.65 µM), tar (2.31 µM)	[33]
**111**		euphoroylean E	Whole plant, EtOH	Chemoreversal, combination abilities on Hep-G2/DOX; IC_50_ > 50 (10.65 µM), tar (2.31 µM)	[33]
**112**	*E. antiquorum*	20-deoxy-16-hydroxyingenol	Stems, MeOH	*α*-glucosidase inhibitory; IC_50_ > 200.00 µM, cytotoxicity (K562; inactive)	[54,55]
**113**	*E. lathyris*	ingenol 6,7-epoxy	Seeds, EtOH	Not evaluated	[31]
**114**	*E. kansuensis*	euphorkanlide A	Roots, EtOH	Cytotoxic (C4-24B; C4-2B/ENZR, MDA-MB-231, IC_50_ = 14.30, 28.20 and > 50 µM respectively). Doxorubicin (0.53, 1.06 and 0.78 µM)	[36]
**Ingenol**					
**115**	*E. saudiarabica*	saudiarabicain A	Aerial, EtOH	Inhibitory (*α*-glucosidase; IC_50_ > 150.00 µM, P-glycoprotein; IC_50_ = 0.80 µM	[28]
**116**		saudiarabicain B	Aerial, EtOH	Inhibitory (*α*-glucosidase; IC_50_ > 150.00 µM. P-glycoprotein control; IC_50_ = 1.40 µM	[28]
**117**	*E. antiquorum*	euphonoid A	Aerial, EtOH	Melanin synthesis (B16; 159.89% at 50.00 µM. 8-MOP; 124.38%)	[34]
**118**		3,8,12-*O*-triacetylingol-7-benzoate	Aerial, EtOH	Melanin synthesis (B16) at 50.00 µM, no activity	[34]
**119**		ingol-3,8,12-*O*-triacetate-7-tiglate	Aerial, EtOH	Melanin synthesis (B16) at 50.00 µM, no activity	[34]
**120**		3,12-*O*-diacetylingol-7-benzoate-8-methoxyl	Aerial, EtOH	Melanin synthesis (B16) at 50.00 µM, no activity	[34]
**121**		3,12-diacetyl-7-angeloyl-8-methoxyingol	Aerial, EtOH	Melanin synthesis (B16) at 50.00 µM, no activity	[34]
**122**		3,12-diacetyl-7-tigloyl-8-methoxyingol	Aerial, EtOH	Melanin synthesis (B16) at 50.00 µM, no activity	[34]
**123**		euphorantin I	Aerial, EtOH	Melanin synthesis (B16; 203.11% at 50.00 µM. 8-MOP; 124.38%)	[34]
**124**		12-acetyl-7-angeloyl-8-methoxyingol	Aerial, EtOH	Melanin synthesis (B16) at 50.00 µM, no activity	[34]
**125**		3,12-diacetyl-ingol-7-tigliate	Aerial, EtOH	Melanin synthesis (B16) at 50.00 µM, No activity	[34]
**126**		3,12-diacetyl-7-angolyl-8-hydroxyingol	Aerial, EtOH	Melanin synthesis (B16) at 50.00 µM, no activity	[34]
**127**		euphorantin J	Aerial, EtOH	Melanin synthesis (B16; 177.43% at 50.00 µM. 8-MOP; 124.38%)	[34]
**128**		tirucalicine	Aerial, EtOH	Melanin synthesis (B16) at 50.00 µM, no activity	[34]
**129**		eurifoloid A	Aerial, EtOH	Melanin synthesis (B16) at 50.00 µM, no activity	[34]
**130**		3-*O*-[(*Z*)-2-methyl-2-butenoyl]-20-*O*-acetylingenol	Aerial, EtOH	Melanin synthesis (B16) at 50.00 µM, no activity	[34]
**131**		eurifoloid L	Aerial, EtOH	Melanin synthesis (B16) at 50.00 µM, no activity	[34]
**132**	*E. antiquorum*	antiquorine A	Aerial, EtOH	Melanin synthesis (B16) at 50.00 µM, no activity	[34]
**Ingol**					
**133**	*E. saudiarabica*	saudiarabicain C	Aerial, EtOH	Inhibitory (*α*-glucosidase; IC_50_ = 9.10 µM. P-glycoprotein IC_50_ = 0.10 µM	[28]
**134**		saudiarabicain D	Aerial, EtOH	Inhibitory (*α*-glucosidase; IC_50_ = 8.00 µM. P-glycoprotein; IC_50_ = 0.10 µM	[28]
**135**		saudiarabicain E	Aerial, EtOH	Inhibitory (*α*-glucosidase; IC_50_ = 1.80 µM. P-glycoprotein; IC_50_ = 0.60 µM	[28]
**136**	*E. royleana*	ingol	Whole plant, EtOH	Chemoreversal, combination abilities on Hep-G2/DOX; IC_50_ > 100 (10.65 µM), tar (2.31 µM)	[33]
**137**		quorumolide C	Whole plant, EtOH	Chemoreversal, combination abilities on Hep-G2/DOX; IC_50_ > 100 (10.65 µM), tar (2.31 µM)	[33]
**138**		(3*S*,4*S*,5*R*,8*S*,10*S*,11*R*,13*R*,14*R*,15R)-3*β*-*O*-angeloyl-17-tigloyloxy-20- deoxyingenol	Whole plant, EtOH	Chemoreversal, combination abilities on Hep-G2/DOX; IC_50_ > 100 (10.65 µM), tar (2.31 µM)	[33,75]
**139**		20-acetyl-ingenol-3-angelate	Whole plant, EtOH	Chemoreversal, combination abilities on Hep-G2/DOX; IC_50_ > 100 (10.65 µM), tar (2.31 µM)	[33]
**140**		3-angelate- 20-hydroxyl-ingenol	whole plant, EtOH	Chemoreversal, combination abilities on Hep-G2/DOX; IC_50_ > 100 (10.65 µM), tar (2.31 µM)	[33]
**141**		(3*S*,4*S*,5*R*,8*S*,10*S*,11*R*,13*R*,14*R*,15*R*) -3*β*-*O*-angeloyl-17-benzoyloxy-20- deoxyingenol	Whole plant, EtOH	Chemoreversal, combination abilities on Hep-G2/DOX; IC_50_ > 100 (10.65 µM), tar (2.31 µM)	[33,75]
**142**	*E. marginata*	euphornan A	Seeds, EtOH	Multidrug reversal activity (Hep-G2/ADR; IC_50_ > 100 µM at 5 µM). Adriamycin control	[41]
**143**		euphornan B	Seeds, EtOH	Multidrug reversal activity (Hep-G2/ADR; IC_50_ > 100 µM at 5 µM). Adriamycin control	[41]
**144**		euphornan C	Seeds, EtOH	Multidrug reversal activity (Hep-G2/ADR; IC_50_ > 100 µM at 5 µM). Adriamycin control	[41]
**145**		euphornan D	Seeds, EtOH	Multidrug reversal activity (Hep-G2/ADR; IC_50_ > 50 µM at 5 µM). Adriamycin control	[41]
**146**		euphornan E	Seeds, EtOH	Multidrug reversal activity (Hep-G2/ADR; IC_50_ > 25 µM at 5 µM). Adriamycin control	[41]
**147**		euphornan F	Seeds, EtOH	Multidrug reversal activity (Hep-G2/ADR; IC_50_ > 25 µM at 5 µM). Adriamycin control	[41]
**148**		euphornan G	Seeds, EtOH	Multidrug reversal activity (Hep-G2/ADR; IC_50_ > 100 µM at 5 µM). Adriamycin control	[41]
**149**		euphornan H	Seeds, EtOH	Multidrug reversal activity (Hep-G2/ADR; IC_50_ > 100 µM at 5 µM). Adriamycin control	[41]
**150**		euphornan I	Seeds, EtOH	Multidrug reversal activity (Hep-G2/ADR; IC_50_ > 25 µM at 5 µM). Adriamycin control	[41]
**151**		euphornan J	Seeds, EtOH	Multidrug reversal activity (Hep-G2/ADR; IC_50_ > 25 µM at 5 µM). Adriamycin control	[41]
**152**		euphornan K	Seeds, EtOH	Multidrug reversal activity (Hep-G2/ADR; IC_50_ > 25 µM at 5 µM). Adriamycin control	[41]
**153**		euphornan L	Seeds, EtOH	Multidrug reversal activity (Hep-G2/ADR; IC_50_ > 25 µM at 5 µM). Adriamycin control	[41]
**154**		euphornan M	Seeds, EtOH	Multidrug reversal activity (Hep-G2/ADR; IC_50_ > 25 µM at 5 µM). Adriamycin control	[41]
**155**		euphornan N	Seeds, EtOH	Multidrug reversal activity (Hep-G2/ADR; IC_50_ > 25 µM at 5 µM). Adriamycin control	[41]
**156**		euphornan O	Seeds, EtOH	Multidrug reversal activity (Hep-G2/ADR; IC_50_ > 25 µM at 5 µM). Adriamycin control	[41]
**157**		euphornan P	Seeds, EtOH	Multidrug reversal activity (Hep-G2/ADR; IC_50_ > 100 µM at 5 µM). Adriamycin control	[41]
**158**		euphornan Q	Seeds, EtOH	Multidrug reversal activity (Hep-G2/ADR; IC_50_ > 25 µM at 5 µM). Adriamycin control	[41]
**159**		euphornan R	Seeds, EtOH	Multidrug reversal activity (Hep-G2/ADR; IC_50_ > 25 µM at 5 µM). Adriamycin control	[41]
**160**		euphornan S	Seeds, EtOH	Multidrug reversal activity (Hep-G2/ADR; IC_50_ > 100 µM at 5 µM). Adriamycin control	[41]
**161**		euphornan T	Seeds, EtOH	Multidrug reversal activity (Hep-G2/ADR; IC_50_ > 100 µM at 5 µM). Adriamycin control	[41]
**162**	*E. resinifera*	euphoresins A	Latex, MeOH	Cytotoxic (MCF-7; IC_50_ = 85.87 µM, C_6_; IC_50_ = 8.31 µM) compared to taxol; 5.48, 6.79 and 8.31 µM respectively	[43]
**163**		euphoresins B	Latex, MeOH	Cytotoxic (MCF-7; IC_50_ = 87.36 µM, C_6_; IC_50_ = 94.89 µM) compared to taxol; 5.48, 6.79 and 8.31 µM respectively	[43]
**164**		euphorantin S	Stem barks, C_3_H_6_O	Anti-HIV-1 (EC_50_ > 44 µM) compared to zidovudine (AZT); EC_50_ = 0.0019 µM	[42]
**165**	*E. neriifolia*	euphorantin T	Stem barks, C_3_H_6_O	Anti-HIV-1 (EC_50_ > 44 µM) compared to zidovudine (AZT); EC_50_ = 0.0019 µM	[42]
**166**		euphorneroid A	Stem barks, C_3_H_6_O	Anti-HIV-1 (EC_50_ > 44 µM) compared to zidovudine (AZT); EC_50_ = 0.0019 µM	[42]
**167**		euphorneroid B	Stem barks, C_3_H_6_O	Anti-HIV-1 (EC_50_ > 44 µM) compared to zidovudine (AZT); EC_50_ = 0.0019 µM	[42]
**168**		euphorneroid C	Stem barks, C_3_H_6_O	Anti-HIV-1 (EC_50_ > 44 µM) compared to zidovudine (AZT); EC_50_ = 0.0019 µM	[42]
**169**		euphorneroid D	Stem barks, C_3_H_6_O	Anti-HIV-1 (EC_50_ = 34 µM) compared to zidovudine (AZT); EC_50_ = 0.0019 µM	[42]
**Isopimarane**					
**170**	*E. pekinensis*	euphopane A	Roots, EtOH	Cytotoxic (C4-24B; C4-2B/ENZR, MDA-MB-231, IC_50_ = 32.30, 29.30 and > 50 µM respectively) compared to doxorubicin (0.53, 1.06 and 0.78 µM)	[44]
**171**		(12*β*)-2,12-dihydroxyisopimara-1,7,15-trien-3-one	Roots, EtOH	Cytotoxic (C4-24B; C4-2B/ENZR, MDA-MB-231, IC_50_ = 32.30, > 50 and > 50 µM respectively) compared to doxorubicin (0.53, 1.06 and 0.78 µM)	[44]
**172**		yuexiandajisu C	Roots, EtOH	Cytotoxic (C4-24B; C4-2B/ENZR, MDA-MB-231, IC_50_ = 23.10, 30.0 and > 50 µM respectively) compared to doxorubicin (0.53, 1.06 and 0.78 µM)	[44]
**173**		(3*β*,12*α*,13*α*)-3,12-dihydroxypimara-7,15-dien-2-one	Roots, EtOH	Cytotoxic (C4-24B; C4-2B/ENZR, MDA-MB-231, IC_50_ = 32.60, > 50 and > 50 µM respectively) compared to doxorubicin (0.53, 1.06 and 0.78 µM)	[44]
**Jatrophane**					
**174**	*E. kansui*	kansuingenol A	Roots, EtOH	Antiproliferative (MCF-7; IC_50_ = 20.86 µM, Hep-G2; IC_50_ = 14.20 µM, DU145; IC_50_ = 6.19 µM)	[65]
**175**		kansuingenol B	Roots, EtOH	Antiproliferative (MCF-7; IC_50_ = 15.82 µM, Hep-G2; IC_50_ = 29.16 µM, DU145; IC_50_ = 9.27 µM)	[65]
**176**		kansuingenol C	Roots, EtOH	Antiproliferative (MCF-7; IC_50_ = 10.26 µM, Hep-G2; IC_50_ = 23.09 µM, DU145; IC_50_ = 26.06 µM)	[65]
**177**		kansuijatrophanol A	Roots, EtOH	Antiproliferative (MCF-7; IC_50_ = 21.64 µM, Hep-G2; IC_50_ = 20.19 µM, DU145; IC_50_ = 7.21 µM)	[65]
**178**		kansuijatrophanol B	Roots, EtOH	Antiproliferative (MCF-7; IC_50_ = 15.25 µM, Hep-G2; IC_50_ = 13.24 µM, DU145; IC_50_ = 7.24 µM)	[65]
**179**		kansuijatrophanol C	Roots, EtOH	Antiproliferative (MCF-7; IC_50_ = 11.25 µM, Hep-G2; IC_50_ = 9.47 µM, DU145; IC_50_ = 8.29 µM)	[65]
**180**		kansuijatrophanol D	Roots, EtOH	Antiproliferative (MCF-7; IC_50_ = 6.29 µM, Hep-G2; IC_50_ = 10.07 µM, DU145; IC_50_ = 4.19 µM)	[65]
**181**	*E. helioscopia*	euphoheliphane A	Aerial, EtOH	Cytotoxic (OS-RC-2; IC_50_ = 47.00 µM, Ketr-3; IC_50_ = 45.00 µM, 769-P; IC_50_ = 43.00 µM, G401; IC_50_ = 38.00 µM, GRC-1; IC_50_ = 41.00 µM, ACHN; IC_50_ = 40.00 µM compared to doxorubicin (DOX); 5, 4, 3, 5, 4, and 3 µM respectively	[50]
**182**		euphoheliphane B	Aerial, EtOH	Cytotoxic (OS-RC-2; IC_50_ = 31.00 µM, Ketr-3; IC_50_ = 32.00 µM, 769-P; IC_50_ = 30.00 µM, G401; IC_50_ = 34.00 µM, GRC-1; IC_50_ = 33.00 µM, ACHN; IC_50_ = 35.00 µM compared to doxorubicin (DOX); 5, 4, 3, 5, 4, and 3 µM respectively	[50]
**183**		euphoheliphane C	Aerial, EtOH	Cytotoxic (OS-RC-2; IC_50_ = 35.00 µM, Ketr-3; IC_50_ = 41.00 µM, 769-P; IC_50_ = 39.00 µM, G401; IC_50_ = 32.00 µM, GRC-1; IC_50_ = 38.00 µM, ACHN; IC_50_ = 36.00 µM compared to doxorubicin (DOX); 5, 4, 3, 5, 4, and 3 µM respectively	[50]
**184**	*E. esula*	euphoesulatin A	Whole plant, EtOH	Ostiosteoporotic activity (BMM; IC_50_ = 1.20 µM) compared to RANKL control	[100]
**185**		euphoesulatin B	Whole plant, EtOH	Ostiosteoporotic activity (BMM; IC_50_ > 10 µM) compared to RANKL control	[100]
**186**		euphoesulatin C	Whole plant, EtOH	Ostiosteoporotic activity (BMM; IC_50_ > 10 µM) compared to RANKL control	[100]
**187**		euphoesulatin D	Whole plant, EtOH	Inhibitory (BMM; IC_50_ = 6.60 µM) compared to RANKL control	[100]
**188**		euphoesulatin E	Whole plant, EtOH	Ostiosteoporotic activity (BMM; IC_50_ = 5.90 µM) compared to RANKL control	[100]
**189**		euphoesulatin F	Whole plant, EtOH	Ostiosteoporotic activity (BMM; IC_50_ = 6.10 µM) compared to RANKL control	[100]
**190**		euphoesulatin G	Whole plant, EtOH	Ostiosteoporotic activity (BMM; IC_50_ = 10.00 µM) compared to RANKL control	[100]
**191**		euphoesulatin H	Whole plant, EtOH	Ostiosteoporotic activity (BMM; IC_50_ = 3.50 µM) compared to RANKL control	[100]
**192**		euphoesulatin I	Whole plant, EtOH	Ostiosteoporotic activity (BMM; IC_50_ > 10 µM) compared to RANKL control	[100]
**193**		euphoesulatin J	Whole plant, EtOH	Ostiosteoporotic activity (BMM; IC_50_ = 2.30 µM) compared to RANKL control	[100]
**194**		euphoesulatin K	Whole plant, EtOH	Ostiosteoporotic activity (BMM; IC_50_ > 10 µM) compared to RANKL control	[100]
**195**		euphoesulatin L	Whole plant, EtOH	Ostiosteoporotic activity (BMM; IC_50_ > 10 µM) compared to RANKL control	[100]
**196**		euphoesulatin M	Whole plant, EtOH	Ostiosteoporotic activity (BMM; IC_50_ = 7.60 µM) compared to RANKL control	[100]
**197**		euphoesulatin N	Whole plant, EtOH	Ostiosteoporotic activity (BMM; not active, compared to RANKL control	[100]
**198**		euphoesulatin O	Whole plant, EtOH	Ostiosteoporotic activity (BMM; IC_50_ = 5.90 µM), compared to RANKL control	[100]
**199**		euphoesulatin P	Whole plant, EtOH	Ostiosteoporotic activity (BMM; IC_50_ > 10 µM) compared to RANKL control	[100]
**200**		euphoesulatin Q	Whole plant, EtOH	Ostiosteoporotic activity (BMM; IC_50_ > 10 µM) compared to RANKL control	[100]
**201**		euphoesulatin R	Whole plant, EtOH	Ostiosteoporotic activity (BMM; IC_50_ > 10 µM) compared to RANKL control	[100]
**202**		esulone B	Whole plant, EtOH	Ostiosteoporotic activity (BMM; No activity) compared to RANKL control	[100]
**203**		kansuinine B	Whole plant, EtOH	Ostiosteoporotic activity (BMM; not active, compared to RANKL control	[59,100]
**204**		esulone A	Whole plant, EtOH	Ostiosteoporotic activity (BMM; not active, compared to RANKL control	[59,100]
**205**		euphoresulane A	Whole plant, EtOH	MDR-chemoreversal (Hep-G2; IC_50_ > 100 µM), adriamycin (ADR); IC_50_ = 284.50 µM	[59]
**206**		euphoresulane B	Whole plant, EtOH	MDR-chemoreversal (Hep-G2; IC_50_ > 25 µM), ADR; IC_50_ = 284.50 µM	[59]
**207**		euphoresulane C	Whole plant, EtOH	MDR-chemoreversal (Hep-G2; IC_50_ > 100 µM), ADR; IC_50_ = 284.50 µM	[59]
**208**		euphoresulane D	Whole plant, EtOH	MDR-chemoreversal (Hep-G2; IC_50_ > 100 µM), ADR; IC_50_ = 284.50 µM	[59]
**209**		euphoresulane E	Whole plant, EtOH	MDR-chemoreversal (Hep-G2; IC_50_ > 100 µM), ADR; IC_50_ = 284.50 µM	[59]
**210**		euphoresulane F	Whole plant, EtOH	MDR-chemoreversal (Hep-G2; IC_50_ > 50 µM), ADR; IC_50_ = 284.50 µM	[59]
**211**		euphoresulane G	Whole plant, EtOH	MDR-chemoreversal (Hep-G2; IC_50_ > 100 µM), ADR; IC_50_ = 284.50 µM	[59]
**212**		euphoresulane H	Whole plant, EtOH	MDR-chemoreversal (Hep-G2; IC_50_ = 165.30 µM, ADR; IC_50_ = 284.50 µM	[59]
**213**		euphoresulane I	Whole plant, EtOH	MDR-chemoreversal (Hep-G2; IC_50_ > 100 µM), ADR; IC_50_ = 284.50 µM	[59]
**214**		euphoresulane J	Whole plant, EtOH	MDR-chemoreversal (Hep-G2; IC_50_ > 100 µM), ADR; IC_50_ = 284.50 µM	[59]
**215**		euphoresulane K	Whole plant, EtOH	MDR-chemoreversal (Hep-G2; IC_50_ > 100 µM), ADR; IC_50_ = 284.50 µM	[59]
**216**		euphoresulane L	Whole plant, EtOH	MDR-chemoreversal (Hep-G2; IC_50_ > 100 µM), ADR; IC_50_ = 284.50 µM	[59]
**217**		euphoresulane M	Whole plant, EtOH	MDR-chemoreversal (Hep-G2; IC_50_ > 100 µM), ADR; IC_50_ = 284.50 µM	[59]
**218**		kanesulone A	Whole plant, EtOH	MDR-chemoreversal (Hep-G2; IC_50_ > 100 µM), ADR; IC_50_ = 284.50 µM	[59]
**219**		3*β*,7*β*,8*α*,15*β*-tetraacetoxy-5*α*-benzoyloxyjatropha-6(17), 11*E*-dien-9,14-dione	Whole plant, EtOH	MDR-chemoreversal (Hep-G2; IC_50_ > 100 µM), ADR; IC_50_ = 284.50 µM	[59]
**220**		kanesulone B	Whole plant, EtOH	MDR-chemoreversal (Hep-G2; IC_50_ > 100 µM), ADR; IC_50_ = 284.50 µM	[59]
**221**		(2*S*,3*S*,4*R*,5*R*,7*S*,8*R*,13*R*,15*R*)−3,5,7,8,15-pentaacetoxy-9,14-dioxojatropha-6(17),11*E*-diene	Whole plant, EtOH	MDR-chemoreversal (Hep-G2; IC_50_ > 100 µM), ADR; IC_50_ = 284.50 µM	[59]
**222**		kansuinin C	Whole plant, EtOH	MDR-chemoreversal (Hep-G2; IC_50_ > 100 µM), ADR; IC_50_ = 284.50 µM	[59]
**223**	*E. glomerulans*	euphoglomeruphane A	Whole plant, C_3_H_6_O	MDR-chemoreversal (MCF-7/ADR IC_50_ > 100 µM), verapamil; IC_50_ = 4.70 µM	[29]
**224**		euphoglomeruphane B	Whole plant, C_3_H_6_O	MDR-chemoreversal (MCF-7/ADR IC_50_ > 100 µM), verapamil; IC_50_ = 4.70 µM	[29]
**225**		euphoglomeruphane C	Whole plant, C_3_H_6_O	MDR-chemoreversal (MCF-7/ADR IC_50_ > 100 µM), verapamil; IC_50_ = 4.70 µM	[29]
**226**		euphoglomeruphane D	Whole plant, C_3_H_6_O	MDR-chemoreversal (MCF-7/ADR IC_50_ > 100 µM), verapamil; IC_50_ = 4.70 µM	[29]
**227**		euphoglomeruphane E	Whole plant, C_3_H_6_O	MDR-chemoreversal (MCF-7/ADR IC_50_ > 100 µM), verapamil; IC_50_ = 4.70 µM	[29]
**228**		euphoglomeruphane F	Whole plant, C_3_H_6_O	MDR-chemoreversal (MCF-7/ADR IC_50_ > 100 µM), verapamil; IC_50_ = 4.70 µM	[29]
**229**		euphoglomeruphane G	Whole plant, C_3_H_6_O	MDR-chemoreversal (MCF-7/ADR IC_50_ > 100 µM), verapamil; IC_50_ = 4.70 µM	[29]
**230**		euphoglomeruphane H	Whole plant, C_3_H_6_O	MDR-chemoreversal (MCF-7/ADR IC_50_ = 39.30 µM), verapamil; IC_50_ = 4.70 µM	[29]
**231**		euphoglomeruphane I	Whole plant, C_3_H_6_O	MDR-chemoreversal (MCF-7/ADR IC_50_ > 100 µM), verapamil; IC_50_ = 4.70 µM	[29]
**232**		euphoglomeruphane J	Whole plant, C_3_H_6_O	MDR-chemoreversal (MCF-7/ADR IC_50_ > 100 µM), verapamil; IC_50_ = 4.70 µM	[29]
**233**		euphoglomeruphane K	Whole plant, C_3_H_6_O	MDR-chemoreversal (MCF-7/ADR IC_50_ > 100 µM), verapamil; IC_50_ = 4.70 µM	[29]
**234**		euphoglomeruphane L	Whole plant, C_3_H_6_O	MDR-chemoreversal (MCF-7/ADR IC_50_ = 50.20 µM), verapamil; IC_50_ = 4.70 µM	[29]
**235**		euphoglomeruphane M	Whole plant, C_3_H_6_O	MDR-chemoreversal (MCF-7/ADR IC_50_ > 100 µM), verapamil; IC_50_ = 4.70 µM	[29]
**236**		euphoglomeruphane N	Whole plant, C_3_H_6_O	MDR-chemoreversal (MCF-7/ADR IC_50_ > 100 µM), verapamil; IC_50_ = 4.70 µM	[29]
**237**		euphoglomeruphane O	Whole plant, C_3_H_6_O	MDR-chemoreversal (MCF-7/ADR IC_50_ > 100 µM), verapamil; IC_50_ = 4.70 µM	[29]
**238**		euphoglomeruphane P	Whole plant, C_3_H_6_O	MDR-chemoreversal (MCF-7/ADR IC_50_ > 100 µM), verapamil; IC_50_ = 4.70 µM	[29]
**239**		euphoglomeruphane Q	Whole plant, C_3_H_6_O	MDR-chemoreversal (MCF-7/ADR IC_50_ > 100 µM), verapamil; IC_50_ = 4.70 µM	[29]
**240**	*E.* *helioscopia*	heliojatrone C	Aerial, EtOH	Inhibitory (nitric oxide (NO) in RAW 264.7; IC_50_ = 7.40 μM) compared to dexamethasone (Dex)	[64]
**241**		heliojatrone D	Aerial, EtOH	Inhibitory (nitric oxide (NO) in RAW 264.7; not active, compared to Dex	[64]
**242**		euphoscopoid E	Aerial, EtOH	Inhibitory (nitric oxide (NO) in RAW 264.7; not active, compared to Dex	[64]
**243**		euphoscopoid F	Aerial, EtOH	Inhibitory (nitric oxide (NO) in RAW 264.7; IC_50_ > 50 μM) compared to Dex	[64]
**244**		euphorhelipanes A	Whole plant, EtOH	Triglyceride lowering effect (HuH7) in range of 1–50 μM compared to rosiglitazone positive control	[99]
**245**		euphorhelipanes B	Whole plant, EtOH	Triglyceride lowering effect (HuH7) in range of 1–50 μM compared to rosiglitazone positive control	[99]
**Kaurane**					
**246**	*E. kansuensis*	abbeokutone	Roots, EtOH	Inhibition of NO (IC_50_ = 43.60 µM; quercetin (IC_50_ = 10.80 µM)	[35]
**Lathyrane**					
**247**	*E. lathyris*	euphorbia factor L_2_	Seeds, EtOH	Not evaluated	[66,105]
**248**		euphorbia factor L_3_	Seeds, EtOH	Not evaluated	[66,105]
**249**	*E. stracheyi*	euphstrachenol A	Roots, MeOH	Cytotoxic (HGC-27; IC_50_ > 50; taxol (0.015 µM) MV4-11; IC_50_ = 12.29; (0.055 µM) BaF3; IC_50_ > 20.00, compared to IC_50_ of 0.015, 0.53 µM, respectively for taxol	[32]
**250**		euphstrachenol B	Roots, MeOH	Cytotoxic (HGC-27; IC_50_ = 49.90; taxol (0.015 µM); MV4-11; IC_50_ = 14.80; (0.055 µM), BaF3; IC_50_ > 20.00, compared to IC_50_ of 0.015, 0.53 µM, respectively for taxol	[32]
**251**		euphstrachenol C	Roots, MeOH	Cytotoxic (HGC-27, MV4-11, BaF3 SKvo3, IC_50_ > 50.00) compared to IC_50_ of 0.015, 0.53 µM, respectively for taxol	[32]
**252**		(2*R*, 3*S*, 4*R*, 5*R*, 9*S*, 11*S*, 15*R*)-3, 5, 15-triacetoxy-14-oxolathyr- 6(17), 12*E*-diene	Roots, MeOH	Cytotoxic (HGC-27; IC_50_ > 50.00; taxol (0.015 µM) MV4-11; IC_50_ = 30.02; taxol (0.055 µM), BaF3; IC_50_ = 19.20,	[32]
**253**		jolkinol B	Roots, MeOH	Cytotoxic (HGC-27; IC_50_ = 39.00; taxol (0.015 µM) MV4-11; IC_50_ = 9.82; (0.055 µM), BaF3; IC_50_ = 11.20, compared to IC_50_ of 0.015, 0.53 µM, respectively for taxol	[32]
**254**		jolkinol A	Roots, MeOH	Cytotoxic (HGC-27, MV4-11, BaF3 SKvo3, IC_50_ > 50.00) compared to IC_50_ of 0.015, 0.53 µM, respectively for taxol	[32]
**255**		jolkinoate C	Roots, MeOH	Cytotoxic (HGC-27; IC_50_ = 32.54; taxol (0.015 µM) MV4-11; IC_50_ = 15.37; (0.055 µM), BaF3; 18.80, SKvo3) compared to IC_50_ of 0.015, 0.53 µM, respectively for taxol	[32]
**256**		jolkinol D	Roots, MeOH	Cytotoxic (HGC-27, MV4-11, BaF3 SKvo3, IC_50_ > 50.00) compared to IC_50_ of 0.015, 0.53 µM, respectively for taxol	[32]
**257**		jolkinoate	Roots, MeOH	Cytotoxic (HGC-27; IC_50_ > 50.00; taxol (0.015 µM), MV4-11; IC_50_ = 5.96; (0.055 µM), BaF3; IC_50_ = 13.40 compared to IC_50_ of 0.015, 0.53 µM, respectively for taxol	[32]
**258**		3*β*, 5*α*, 20-trihydroxy-15*β*-cinnamoyloxy-14-oxolathyra-6*Z*, 12*E-*diene	Roots, MeOH	Cytotoxic (HGC-27, MV4-11, BaF3 SKvo3, IC_50_ > 50.00), taxol (0.015 µM)	[32]
**259**		yuexiandajisu C	Roots, MeOH	Cytotoxic (HGC-27; IC_50_ > 50.00; taxol (0.015 µM), MV4-11; IC_50_ = 12.24; (0.055 µM), BaF3; IC_50_ = 13.40 µM compared to IC_50_ of 0.015, 0.53 µM, respectively for taxol	[32]
**260**		jolkinolide E	Roots, MeOH	Cytotoxic (HGC-27, MV4-11, BaF3 SKvo3, IC_50_ > 50.00), (0.015 µM compared to IC_50_ of 0.015, 0.53 µM, respectively for taxol	[32]
**261**		stracheyioid C	Roots, MeOH	Cytotoxic (HGC-27, MV4-11, BaF3 SKvo3, IC_50_ > 50.00), (0.015 µM compared to IC_50_ of 0.015, 0.53 µM, respectively for taxol	[32]
**262**	*E. royleana*	ingol-3,7,12-triacetate-8-benzoate	Whole plant, EtOH	MDR-chemoreversal (Hep-G2/DOX; IC_50_ = 4.76 µM, Dox; 499.88 µM	[33]
**263**		ingol-3,8,12-triacetate-7-tiglate	Whole plant, EtOH	MDR-chemoreversal (Hep-G2/DOX; IC_50_ = 27.29 µM, dox; 499.88 µM	[33]
**264**		3,7,12-*O*-triacetyl-8-*O*-(2-methylbutanoyl)-ingol	Whole plant, EtOH	MDR-chemoreversal (Hep-G2/DOX; IC_50_ = 18.98 µM, dox; 499.88 µM	[33]
**265**		euphorantin M	Whole plant, EtOH	MDR-chemoreversal (Hep-G2/DOX; IC_50_ = 20.81 µM, dox; 499.88 µM	[33]
**266**		3,12-di-*O*-acetyl-8-*O*-tigloyl-ingol	Whole plant, EtOH	Chemoreversal, combination abilities on Hep-G2/DOX; IC_50_ > 100 (10.65 µM), tar (2.31 µM)	[33]
**267**		8-*O*-methyl-ingol-3,12-diacetate-7-benzoate	Whole plant, EtOH	Chemoreversal, combination abilities on Hep-G2/DOX; IC_50_ > 100 (10.65 µM), tar (2.31 µM)	[33]
**268**		3,8,12-*O*-triacetylingol-7-benzoate	Whole plant, EtOH	MDR-chemoreversal (Hep-G2/DOX; IC_50_ = 11.18 µM, dox; 499.88 µM	[33]
**269**		8-*O*-methylingol-3,8,12-triacetate-7-angelate	Whole plant, EtOH	MDR-chemoreversal (Hep-G2/DOX; IC_50_ = 17.83 µM, dox; 499.88 µM	[33]
**270**		3,12-diacetyl-8-benzoylingol	Whole plant, EtOH	MDR-chemoreversal (Hep-G2/DOX; IC_50_ = 17.83 µM, dox; 499.88 µM	[33]
**271**		8-*O*-methylingol-12-acetate-7-angelate	Whole plant, EtOH	Chemoreversal, combination abilities on Hep-G2/DOX; IC_50_ > 100 (10.65 µM), tar (2.31 µM)	[33]
**272**		ent-atis-16-ene-3,14-dione	Whole plant, EtOH	Chemoreversal, combination abilities on Hep-G2/DOX; IC_50_ > 100 (10.65 µM), tar (2.31 µM)	[33]
**273**		eurifoloid L	Whole plant, EtOH	Chemoreversal, combination abilities on Hep-G2/DOX; IC_50_ > 100 (10.65 µM), tar (2.31 µM)	[33]
**274**		eurifoloid J	Whole plant, EtOH	Chemoreversal, combination abilities on Hep-G2/DOX; IC_50_ > 100 (10.65 µM), tar (2.31 µM)	[33]
**275**		eurifoloid G	Whole plant, EtOH	Chemoreversal, combination abilities on Hep-G2/DOX; IC_50_ > 100 (10.65 µM), tar (2.31 µM)	[33]
**276**		eurifoloid E	Whole plant, EtOH	Chemoreversal, combination abilities on Hep-G2/DOX; IC_50_ > 100 (10.65 µM), tar (2.31 µM)	[33]
**277**		antiquorine A	Whole plant, EtOH	Chemoreversal, combination abilities on Hep-G2/DOX; IC_50_ > 100 (10.65 µM), tar (2.31 µM)	[33]
**278**		5, 15-di-*O*-acetoxy-3-nicotinoyllathyol-6, 13(20)-diene-12-ol-14-one	Seeds, EtOH	MDR-chemoreversal (Hep-G2; IC_50_ > 100.00 µM, ADR; IC_50_ = 28.00 µM	[106]
**279**		5, 15,17-*O*-tri- acetyl-3-*O*-nicotinoyllathyol-6,12-diene -14-one	Seeds, EtOH	MDR-chemoreversal (Hep-G2; IC_50_ = 37.25 µM, ADR; IC_50_ = 14.81	[106]
**280**		15-*O*-acetoxy-3,7-di-*O*-benzoyllathyra-6(17),12-diene-5-ol-14-one	Seeds, EtOH	MDR-chemoreversal (Hep-G2; IC_50_ = 66.05 µM, ADR; IC_50_ = 27.09 µM	[106]
**281**		15-*O*-acetyl-3-*O*-phenlacetate-6, 17-epoxylathyra-5-ol-14-one	Seeds, EtOH	MDR-chemoreversal (Hep-G2; IC_50_ > 100.00 µM, ADR; IC_50_ > 100.00 µM	[106]
**282**		euphorbia factor L_9_	Seeds, EtOH	Ant-HIV-1; inactive compared to zidovudine positive control	[75]
**283**		euphorbia factor L_15_	Seeds, EtOH	Ant-HIV-1; inactive compared to zidovudine control	[75]
**284**		euphorbia factor L_8_	Seeds, EtOH	Ant-HIV-1; inactive compared to zidovudine control	[75]
**285**		15-*O*-acetyl-3-*O*-nicotinoyljolkinol-5*β*,6*β*-oxide	Seeds, EtOH	Ant-HIV-1; inactive compared to zidovudine control	[75]
**286**		15,17-di-*O*-acetyl-3-*O*-hexanoyl-17-hydroxyjolkinol	Seeds, EtOH	Ant-HIV-1; inactive compared to zidovudine control	[75]
**287**		15,17-di-*O*-acetyl-3-*O*-benzoyl-17-hydroxyjolkinol	Seeds, EtOH	Ant-HIV-1; inactive compared to zidovudine control	[75]
**288**		15,17-di-*O*-acetyl-3-*O*-cinnamoyl-17-hydroxyjolkinol	Seeds, EtOH	Ant-HIV-1; inactive compared to zidovudine control	[75]
**289**		15-*O*-acetyl-3-*O*-cinnamoyl-17-hydroxyjolkinol	Seeds, EtOH	Ant-HIV-1; inactive compared to zidovudine control	[75]
**290**		15-*O*-acetyl-3-*O*-phenylacetyl-17-hydroxyjolkinol	Seeds, EtOH	Ant-HIV-1; inactive compared to zidovudine control	[75]
**291**		15-acetoxy-3-*O*-hexanoyl-17-hydroxyjolkinol-12-en-17-ol-14-one	Seeds, EtOH	Ant-HIV-1; inactive compared to zidovudine control	[75]
**292**		5,15,17-tri-*O*-acetyl-3-*O*-benzoyl-17-hydroxyisolathyrol	Seeds, EtOH	Ant-HIV-1; inactive compared to zidovudine control	[75]
**293**		5,15-di-*O-*acetyl-3-*O*-benzoyl-17-hydroxyisolathyrol	Seeds, EtOH	Ant-HIV-1; inactive compared to zidovudine control	[75]
**294**		5,15-di-acetoxy-3-nicotinoyloxy-6,17-epoxylathyra-12-en-14-one	Seeds, EtOH	Ant-HIV-1; inactive compared to zidovudine control	[75]
**295**		ingenol-20-*O*-decanoyl	Seeds, EtOH	Ant-HIV-1; inactive compared to zidovudine control	[75]
**296**	*E. antiquorum*	euphonoid B	Aerial, EtOH	Melanin synthesis (B16) at 50.00 µM, inactive	[34]
**297**		euphonoid C	Aerial, EtOH	Melanin synthesis (B16) at 50.00 µM, inactive	[34]
**298**		euphonoid D	Aerial, EtOH	Melanin synthesis (B16) at 50.00 µM, inactive	[34]
**299**		euphonoid E	Aerial, EtOH	Melanin synthesis (B16) at 50.00 µM, inactive	[34]
**300**	*E. kansuensis*	euphanoid A	Roots, EtOH	Inhibitory (NO in RAW264.7; IC_50_ = 4.70 µM), quercetin (IC_50_ = 10.80 µM)	[35]
**301**		euphanoid B	Roots, EtOH	Inhibitory (NO in RAW264.7; IC_50_ = 9.50 µM), quercetin (IC_50_ = 10.80 µM)	[35]
**302**	*E. lathyris*	(2*S*,3*S*,4*S*,5*R*,9*S*,11*R*,15*R*)-15-acetoxy-3-cinnamoyloxy-5-hydroxy-14-oxolathyra-6(17),12*E*-diene	Seeds, EtOH	Inhibitory (NO in RAW264.7; IC_50_ = 3.00 µM compared to dexamethasone (7.9 µM)	[103]
**303**		(2*S*,3*S*,4*S*,5*R*,7*R*,9*S*,11*R*,15*R*)-7,15-diacetoxy-3-benzoyloxy-5-hydroxy-14-oxolathyra 6(17),12*E*-diene	Seeds, EtOH	Inhibitory (NO in RAW264.7; IC_50_ = 4.00 µM compared to dexamethasone (7.9 µM)	[103]
**304**		(2*S*,3*S*,4*R*,5*R*,7*R*,9*S*,11*R*,15*R*)-5,15-diacetoxy-3-benzoyloxy-7-hydroxy-14-oxolathyra-6(17),12*E*-diene	Seeds, EtOH	Inhibitory (NO in RAW264.7; IC_50_ = 5.00 µM compared to dexamethasone (7.9 µM)	[103]
**305**		(2*S*,3*S*,4*R*,9*S*,11*R*,15*R*)-15,17-diacetoxy-3-hydroxy-14-oxolathyra-5*E*,12*E*-diene	Seeds, EtOH	Inhibitory (NO in RAW264.7; IC_50_ > 50.0 µM compared to dexamethasone (7.9 µM)	[103]
**306**		(2*S*,3*S*,4*R*,5*R*,7*R*,9*S*,11*R*,15*R*)-5,15-diacetoxy-3-benzoyloxy-7-hydroxy-14-oxolathyra-6(17),12*E*-diene	Seeds, EtOH	Inhibitory (NO in RAW264.7; IC_50_ > 50.0 µM compared to dexamethasone (7.9 µM)	[103]
**307**		(2*S*,3*S*,4*R*,5*R*,9*S*,11*R*,15*R*)-3-benzoyloxy-5,17-diacetoxy-15-hydroxy-14-oxolathyra-6*E*,12*E*-diene	Seeds, EtOH	Inhibitory (NO in RAW264.7; IC_50_ > 100.0 µM compared to dexamethasone (7.9 µM)	[103]
**308**	*E.* *antiquorum*	euphorin C	Stems, MeOH	Inhibitory (NO production in BV-2; inactive	[56]
**309**		euphorin D	Stems, MeOH	Inhibitory (NO production in BV-2; IC_50_ = 32.00 µM); SMT (4.2 µM)	[56]
**310**		euphorin E	Stems, MeOH	Inhibitory (NO production in BV-2; IC_50_ = 40.70 µM), SMT (4.2 µM)	[56]
**Meroterpenoid**					
**311**	*E. fischeriana*	fischernolide A	Roots, EtOH: H_2_O (95:5)	Cytotoxic (Bel-7402; IC_50_ = 27.30, HT; IC_50_ = 49.61 µM, A549; IC_50_ = 20.53 µM, MCF-7; IC_50_ = 33.70 µM, HeLa; IC_50_ = 35.65 µM) compared to cisplatin; 11.9, 33.48, 12.02, 12.78, 8.65 µM respectively	[30]
**312**		fischernolide B	Roots, EtOH: H_2_O (95:5)	Cytotoxic (Bel-7402; IC_50_ = 5.04 µM, HT; IC_50_ = 7.59 µM, A549; IC_50_ = 8.69 µM, MCF-7; IC_50_ = 4.95 µM, HeLa; IC_50_ = 7.53 µM) compared to cisplatin; 11.9, 33.48, 12.02, 12.78, 8.65 µM respectively	[30]
**313**		fischernolide C	Roots, EtOH: H_2_O (95:5)	Cytotoxic (Bel-7402; IC_50_ = 3.30 µM, HT; IC_50_ = 4.21 µM, A549; IC_50_ = 3.27, µM MCF-7; IC_50_ = 2.04 µM, HeLa; IC_50_ = 4.22 µM) compared to cisplatin; 11.9, 33.48, 12.02, 12.78, 8.65 µM respectively	[30]
**314**		fischernolide D	Roots, EtOH: H_2_O (95:5)	Cytotoxic (Bel-7402; IC_50_ = 11.96 µM, HT; IC_50_ = 33.48 µM, A549; IC_50_ = 9.57 µM, MCF-7; IC_50_ = 14.98 µM, HeLa; IC_50_ = 10.22 µM) compared to cisplatin; 11.9, 33.48, 12.02, 12.78, 8.65 µM respectively	[30]
**315**		fischeriana A	Roots, EtOH	Not evaluated	[47]
**Mysrinane**					
**316**	*E. prolifera*	5*α*,10*β*,14*β*,15*β*-*O*-tetraacetyl-8*β*-*O*-benzoyl-3*β*-*O*-nicotinoylcyclomyrsinol	Roots, MeOH	Lipid-lowering activity in 3T3-L1 adipocytemodel using R17 control	[37]
**317**		5*α*,10*β*,14*β*,15*β*-*O*-tetraacetyl-8*β*-*O*-isobutyryl-3*β*-*O*-nicotinoylcyclomyrsinol	Roots, MeOH	Lipid-lowering activity in 3T3-L1 adipocytemodel using R17 control	[37]
**318**		5*α*,7*β*,10,14*β*,15*β*-*O*-pentaacetyl-3*β*-*O*-butyryl-14-desoxo-10,18-dihydromyrsinol	Roots, MeOH	Lipid-lowering activity in 3T3-L1 adipocytemodel using R17 control	[37]
**319**		5*α*,7*β*,10,14*β*,15*β*-*O*-pentaacetyl-14-desoxo-10,18-dihydro-3*β*-*O*-propionylmyrsinol	Roots, MeOH	Lipid-lowering activity in 3T3-L1 adipocytemodel using R17 control	[37]
**320**		5*α*,7*β*,10,15*β*-*O*-tetraacetyl-3*β*-*O*-benzoyl-14-desoxo-10,18-dihydro-14*α*-*O*-nicotinoylmyrsinol	Roots, MeOH	Lipid-lowering activity in 3T3-L1 adipocytemodel using R17 control	[37]
**321**		3*β*,5*α*,7*β*,10,15*β*-*O*-pentaacetyl-14*α*-*O*-benzoyl-14-desoxo-10,18-dihydro-2*α*-hydroxylmyrsinol	Roots, MeOH	Lipid-lowering activity in 3T3-L1 adipocytemodel using R17 control	[37]
**322**		7*β*,13*β*,17-*O*-triacetyl-5-*O*-benzoyl-3*β*-*O*-nicotinoylpremyrsinol	Roots, MeOH	Lipid-lowering activity in 3T3-L1 adipocytemodel using R17 control	[37]
**Paralianone**					
**323**	*E. peplus*	8*β*-acetyl- paralianone D	Whole plant, CH_3_OH	Cytotoxic (HL-60, A-549, SMMC-7721, MCF-7, SW480). Inactive at 40 µM, using paclitaxel and cisplatin as control. Enhanced the LysoTracker intensity of 132.6% at 3.20 µM using DMSO as the control	[38]
**324**		paralianone	Whole plant, CH_3_OH	Cytotoxic (HL-60, A-549, SMMC-7721, MCF-7, SW480). Inactive at 40 µM, using paclitaxel and cisplatin as control.	[38]
**325**		paralianone D	Whole plant, CH_3_OH	Cytotoxic (HL-60, A-549, SMMC-7721, MCF-7, SW480). Inactive at 40 µM, using paclitaxel and cisplatin as control.	[38]
**Pimarane**					
**326**	*E. stracheyi*	(3*β*, 12*α*, 13*α*)-3, 12-dihydrxypiar-7, 15-dien-2-one	Roots, MeOH	Not evaluated	[32]
**327**		(5*β*, 9*β*, 10*α*)-2-hydroxypimara-1, 7, 15-trien-3-one	Roots, MeOH	Not evaluated	[32]
**328**		(3*α*, 5*β*, 8*α*, 9*β*, 10*α*, 12*α*)-3-hydroxytis-16-en-14-one	Roots, MeOH	Not evaluated	[32]
**Premyrsinane**					
**329**	*E. sanctae-catharinae*	euphosantianane E	Aerial, CH_2_CI_2_: MeOH	Not evaluated	[26]
**330**		euphosantianane F	Aerial, CH_2_CI_2_: MeOH	Not evaluated	[26]
**331**		euphosantianane G	Aerial, CH_2_CI_2_: MeOH	Not evaluated	[26]
**Rosane**					
**332**	*E. ebracteolata*	ebraphenol A	Roots, EtOH (n-BuOH, EtOAc)	Lipase inhibitory (IC_50_ = 1.00 µM) compared to lovastatin positive control; IC_50_ = 0.24 µM	[48]
**333**		ebraphenol B	Roots, EtOH (n-BuOH, EtOAc)	Lipase inhibitory (IC_50_ = 0.24 µM) compared to lovastatin positive control; IC_50_ = 0.24 µM	[48]
**334**		ebraphenol C	Roots, EtOH (n-BuOH, EtOAc)	Lipase inhibitory (IC_50_ = 0.24 µM) compared to lovastatin positive control; IC_50_ = 0.24 µM	[48]
**335**		ebraphenol D	Roots, EtOH (n-BuOH, EtOAc)	Lipase inhibitory (IC_50_ = 0.24 µM) compared to lovastatin positive control; IC_50_ = 0.24 µM	[48]
**336**		ebralactone A	Roots, EtOH (n-BuOH, EtOAc)	Lipase inhibitory (IC_50_ = 0.24 µM) compared to lovastatin positive control; IC_50_ = 0.24 µM	[48]
**337**	*E. neriifolia*	euphnerin A	Stems, MeOH	NO inhibitory (BV-2, IC_50_ = 22.00 µM) compared to SMT positive control 2.00 µM	[71]
**338**		euphnerin B	Stems, MeOH	NO inhibitory (BV-2, IC_50_ = 30.00 µM) compared to SMT positive control 2.00 µM	[71]
**Tigliane**					
**339**	*E. fischeriana*	prostratin 20-*O*-(6′-acetate)-*β*-D-glucopyranoside	Roots, EtOH	Cytotoxic (AGS; IC_50_ = 40.56 µM, Hep-G2; IC_50_ = 27.97 µM) compared to oxaliplatin; IC_50_ of 17.06 and 24.26 µM respectively	[63]
**340**		fischeroside A	Roots, EtOH	Cytotoxic (AGS; IC_50_ = 27.97 µM, Hep-G2; IC_50_ = 17.59 µM) compared to oxaliplatin; IC_50_ of 17.06 and 24.26 µM respectively	[63]
**341**		12-deoxyphorbol-13-dimethylpentadecanoate	Roots, MeOH	Lysosomal biogenesis activity (183.21%) using blank control	[102]
**342**		17-hydroxy,11α, 8(14) epoxy-ent-abieta-13(15)-ene-11,12-dioxide	Roots, MeOH	Lysosomal biogenesis activity (181.95%) using blank control	[102]
**343**	*E. lathyris*	eupholathone	Seeds, EtOH	Not evaluated	[66]
**344**	*E. grandicornis*	16-angeloyloxy-13*α* -isobutanoyloxy-4*β*, 9*α*-dihydroxytiglia-1, 6- dien-3-one.	Aerial, MeOH	Protein kinase C activation and platelet stimulation abilities	[52]
**345**		20-acetoxy-13*α*-isobutanoyloxy-4*β*, 9*α*, 16-trihydroxytiglia-1, 6-dien-3-one.	Aerial, MeOH	Protein kinase C activation and platelet stimulation abilities	[52]
**346**	*E. dracunculoides*	4-deoxy-4(*β*)H-8-hydroperoxyphorbol-12-benzoate-13-isobutyrate	Whole plant, EtOH	Not evaluated	[57]
**Myrsinol**					
**347**	*E. prolifera*	euphorbialoid K	Roots, MeOH	Not evaluated	[74]
**348**		euphorbialoid L	Roots, MeOH	Not evaluated	[74]
**349**		euphorbialoid M	Roots, MeOH	Not evaluated	[74]
**350**		euphorbialoid N	Roots, MeOH	Not evaluated	[74]
**351**	*E. dracunculoides*	euphordracunculin A	Aerial, EtOH	Cytotoxic (HL-60, SMMMC-7721, A-549, MCF-7, SW-480); Inactive (IC_50_ > 40 µM)	[107]
**352**		euphordracunculin B	Aerial, EtOH	Cytotoxic (HL-60, SMMMC-7721, A-549, MCF-7, SW-480); Inactive (IC_50_ > 40 µM)	[107]
**Paraliane**					
**353**	*E. esula*	euphorbesulin D	Twigs, EtOH	Antimalarial (IC_50_ > 50 µM) compared to artemisinin (7.01 µM) as a positive control	[49]
**354**	*E. peplus*	pepluanol A	Whole plant, C_3_H_6_O	Inhibition of LPS-stimulated NO production in RAW264.7 cells (IC_50_ > 50 µM) compared to proteasome inhibitor (MG-132) with IC_50_ of 0.18 µM	[72]
**355**		pepluanol B	Whole plant, C_3_H_6_O	Inhibition of LPS-stimulated NO production in RAW264.7 cells (IC_50_ > 50 µM) compared to proteasome inhibitor (MG-132) with IC_50_ of 0.18 µM	[72]
**356**		pepluanol C	Whole plant, C_3_H_6_O	Inhibition of LPS-stimulated NO production in RAW264.7 cells (IC_50_ > 50 µM) compared to proteasome inhibitor (MG-132) with IC_50_ of 0.18 µM	[72]
**357**		pepluanol D	Whole plant, C_3_H_6_O	Inhibition of LPS-stimulated NO production in RAW264.7 cells (IC_50_ > 50 µM) compared to proteasome inhibitor (MG-132) with IC_50_ of 0.18 µM	[72]
**358**		pepluanol E	Whole plant, C_3_H_6_O	Inhibition of LPS-stimulated NO production in RAW264.7 cells (IC_50_ > 50 µM) compared to proteasome inhibitor (MG-132) with IC_50_ of 0.18 µM	[72]
**359**		pepluanol F	Whole plant, C_3_H_6_O	Inhibition of LPS-stimulated NO production in RAW264.7 cells (IC_50_ > 50 µM) compared to proteasome inhibitor (MG-132) with IC_50_ of 0.18 µM	[72]
**360**		pepluanol G	Whole plant, C_3_H_6_O	Inhibition of LPS-stimulated NO production in RAW264.7 cells (IC_50_ = 36.6 µM) compared to proteasome inhibitor (MG-132) with IC_50_ of 0.18 µM	[72]
**361**		pepluanol H	Whole plant, C_3_H_6_O	Inhibition of LPS-stimulated NO production in RAW264.7 cells (IC_50_ > 50 µM) compared to proteasome inhibitor (MG-132) with IC_50_ of 0.18 µM	[72]
**Pepluane**					
**362**	*E. peplus*	paralianone A	Whole plant, C_3_H_6_O	Inhibition of LPS-stimulated NO production in RAW264.7 cells (IC_50_ = 43.2 µM) compared to proteasome inhibitor (MG-132) with IC_50_ of 0.18 µM	[72]
**363**		paralianone B	Whole plant, C_3_H_6_O	Inhibition of LPS-stimulated NO production in RAW264.7 cells (IC_50_ > 50 µM) compared to proteasome inhibitor (MG-132) with IC_50_ of 0.18 µM	[72]
**364**		paralianone C	Whole plant, C_3_H_6_O	Inhibition of LPS-stimulated NO production in RAW264.7 cells (IC_50_ = 33.7 µM) compared to proteasome inhibitor (MG-132) with IC_50_ of 0.18 µM	[72]
**365**		paralianone D	Whole plant, C_3_H_6_O	Inhibition of LPS-stimulated NO production in RAW264.7 cells (IC_50_ = 38.3 µM) compared to proteasome inhibitor (MG-132) with IC_50_ of 0.18 µM	[72]
**Presegetane**					
**366**	*E. esula*	euphorbesulin A	Twigs, EtOH	Antimalarial (IC_50_ = 2.41 µM) compared to artemisinin (7.01 µM) as a positive control	[49]
**367**		euphorbesulin B	Twigs, EtOH	Antimalarial (IC_50_ > 5 µM) compared to artemisinin (7.01 µM) as a positive control	[49]
**368**		euphorbesulin C	Twigs, EtOH	Antimalarial (IC_50_ > 5 µM) compared to artemisinin (7.01 µM) as a positive control	[49]
**Others**					
**369**	*E. aellenii*	3-nicotinyl-5,10,14,15- tetraacetyl-8-(20,30-dimethyl butanoyl)-cyclomyrsinol	Aerial, C_3_H_6_O: CHCl_3_ (1:2)	Lymphocytes proliferative effects (*p* > 0.05, at 50 µg/mL) using stimulated and unstimulated T cells in absence of the compound as the control	[39]
**370**		3,5,10,14,15-pentaacetyl-8-isobutanoyl cyclomyrsinol	Aerial, C_3_H_6_O: CHCl_3_ (1:2)	Lymphocytes proliferative effects (*p* > 0.05, at 50 µg/mL) using stimulated and unstimulated T cells in absence of the compound as the control	[39]
**371**	*E. pilosa*	euphopiloside A	Whole plant, EtOH	Cytotoxic (HL-60, SMMMC-7721, A-549, MCF-7, SW-480); moderate activity compared to cisplatin with IC_50_ values of 3.29, 9.26, 9.98, 15.92 and 14.43 µM respectively	[40]
**372**		euphopiloside B	Whole plant, EtOH	Cytotoxic (HL-60, SMMMC-7721, A-549, MCF-7, SW-480); moderate activity compared to cisplatin with IC_50_ values of 3.29, 9.26, 9.98, 15.92 and 14.43 µM respectively	[40]
**373**	*E. kansui*	euphorikanin A	Roots, EtOH	Cytotoxic effect (HeLa; IC_50_ = 20.89 µM, NCI-446; 28.83 µM compared to etoposide (IC_50_ of 26.23 and 30.68 µM respectively)	[53]
**374**	*E. esula*	euphorbesulin E	Twigs, EtOH	Antimalarial (IC_50_ > 50 µM) compared to artemisinin (7.01 µM) as a positive control	[49]
**375**	*E. dracunculoides*	euphordracunculin C	Aerial, C_3_H_6_O: H_2_O (7:3)	Not evaluated	[107]
**376**	*E. peplus*	pepluacetal	Roots, MeOH	Inhibition of Kv1.3 channel with IC_50_ value of 24.9 µM	[72]
**377**		pepluanol A	Roots, MeOH	Inhibition of Kv1.3 channel with IC_50_ value of 46.0 µM	[72]
**378**		pepluanol B	Roots, MeOH	Inhibition of Kv1.3 channel with IC_50_ value of 9.50 µM	[72]
**379**	*E. micractina*	secoeuphoractin	Roots, EtOH	Anti-HIV-1 replication ability (IC_50_ = 1.76 µmol/L) compared to zidovudine (0.005 µmol/L) as positive control	[67]
**380**		euphorbactin	Roots, EtOH	Anti-HIV-1 replication ability (IC_50_ = 28.6 µM) compared to zidovudine (0.005 µM) as positive control	[104]
**381**	*E. kopetdaghi*	kopetdaghinane A	Aerial, CH_2_CI_2_: C_3_H_6_O (2:1)	Cytotoxic (MCF-7; IC_50_ = 38.10 µM, OCVAR-3; IC_50_ = 51.23 µM) compared to taxol (44.61 and 52.3 µM respectively)	[25]
**382**		kopetdaghinane B	Aerial, CH_2_CI_2_: C_3_H_6_O (2:1)	Cytotoxic (MCF-7; IC_50_ =38.10 µM, OCVAR-3; IC_50_ = 51.23 µM) compared to taxol (44.61 and 52.3 µM respectively)	[25]

## 6. Pharmacological Activities and Structure–Activity Relationship (SAR)

**Figure 16:**
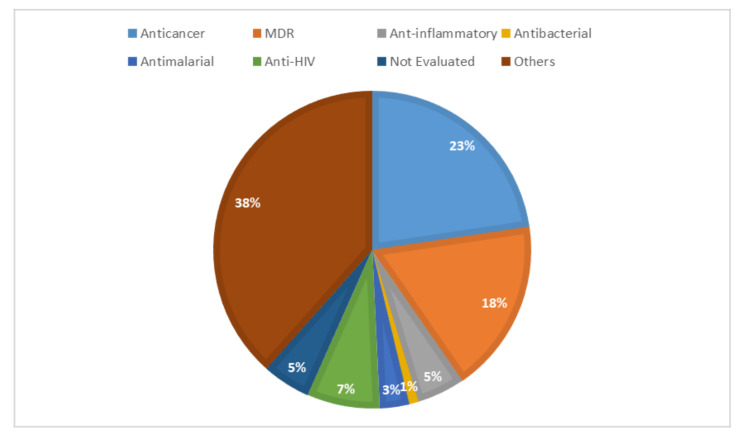
Distribution of publications describing different biological activities.

Due to the ethnomedicinal usage of *Euphorbia* species in the prevention and treatment of various ailments, and the structural diversity of isolated compounds, different publications reported various biological studies. Analysis of the reported biological studies revealed that most of the publications explored cytotoxic effects and anti-inflammatory activities [10,13,108]. This was followed closely by the chemoreversal studies. Most of the studied species reported new bioactive diterpenes, particularly as anticancer agents. Antibacterial and antimalarial biological activities were the least studied, while a significant number (5%) of isolated diterpenes were not evaluated (Figure 16). Many of the reported biological studies used appropriate controls while few studies lacked information about the controls used. The structure–activity relationship (SAR) of these diterpenes revealed that acetylation and esterification of hydroxyl groups, particularly at C-3 and C-8, have a positive effect on these activities. 

### 6.1. Anticancer Activities

Biological evaluation of seven diterpenes, isolated from whole-plant extracts of *E. peplus,* against five human cancer cell lines—human leukemia cells (HL-60), human lung cancer cells (A-549), liver cancer cells (SMMC-7721), breast cancer cells (MCF-7), and colon cancer cells (SW480)—showed no significant activities. The abietane diterpenes; 11,12-didehydro-8*α*,14-dihydro-7-oxo-helioscopinolide A (**1**) [38] and 8*β*-acetyl-paralianone D (**2**) [38] were further investigated for their ability to boost lysosomal biogenesis. The results showed that 8*β*-acetyl-paralianone D (**2**) [38] increased the LysoTracker staining intensity with a percentage value of 132.60% at 40 µM, using paclitaxel and cisplatin as control, while the other compounds showed no effect [38]. 

Evaluation of cytotoxic activities of *ent*-11*β*-hydroxyabieta-8(14),13(15)-dien-16,12-olide (**3**) isolated from *E. stracheyi* showed no significant activity against stomach cancer cell lines (HGC-27), leukemia cells (MV4-11), murine cell line lymphocyte (BaF3), and ovarian carcinoma (SKvo3) with IC_50_ value of >50.00 µM compared to taxol, the positive control [32]. Abietane diterpenoids; 1*α*,9*β*-dihydroxy-ent-abieta-8(14),13(15)-dien-16,12-olide (**4**) and 1*α*-hydroxy-14-oxo-ent-abieta-8,13(15)-dien-16,12-olide (**5**) isolated from *E. neriifolia* exhibited no antiangiogenic activity (HUVECs migration) with an IC_50_ value of >50.00 µg/mL [68]. Cembrane diterpene euphopane C, from the root extracts of *E. pekinensis,* displayed cytotoxic activities against C4-24B; C4-2B/ENZR, MDA-MB-231 with IC_50_ values of 32.30, 29.30 and >50 µM, respectively, compared to doxorubicin (0.53, 1.06 and 0.78 µM respectively) [44], while euphoroylean A (**8**) and euphoroylean B (**9**), from the whole plant extract of *E. royleana,* showed chemoreversal, combination abilities on Hep-G2/DOX; IC_50_ > 50 µM, compared to verapamil [33]. Euphoractone (**14**) from *E. fischeriana* exhibited cytotoxic on H23; IC_50_ = 21.07 mmol/L, H460; IC_50_ = 20.91 mmol/L) using cisplatin as a positive control [60]. In previous studies, evaluation of antitumor effects of 17-acetoxyjolkinolide B and six analogs from *E. fischeriana* revealed that these compounds irreversibly inhibited the NF-κB signaling pathway through direct interaction with inhibitory κB kinases (IKK-β). Additionally, 17-acetoxyjolkinolide B induced apoptosis of tumor cells and acted synergistically with anticancer drugs [109]. Among the tigliane-type diterpenes of *E. fischeriana*, only 12-deoxyphorbol 13-hexadecanoate showed cytotoxicity against MDA-MB-231 cells with an IC_50_ value of 6.694 μM. Fischerianoids A-C (**26**–**28**) from *E.* *fischeriana* showed varied cytotoxic against HL-60, MM-23, A549, SMMC-7721 and Hep-3B. All the three ent-abietane diterpenes (**26**–**28**) were active against MM-23 with IC_50_ values of 12.10, 9.12, and 25.45 µM respectively [61]. 

The antiproliferative activities of previously undescribed tigliane diterpenoids isolated from *E. fischeriana* were evaluated in vitro against human gastric cancer cell lines (AGS) and human liver cancer cells (Hep-G2) using cell counting kit-8 (CCK-8) assay. These diterpenes exhibited potent activities against AGS cells. Among the diterpenoids, prostratin 20-*O*-(6′-acetate)-*β*-D-glucopyranoside (**339**) [63], and fischeroside A (**340**) [63] exhibited significant activities with IC_50_ values of 40.56 µM and 22.49 µM against AGS and 27.97 µM and 17.59 µM against Hep-G2, respectively, compared to oxaliplatin with IC_50_ values of 17.06 and 24.26 µM, respectively. The findings suggest that *E. fischeriana* is rich in bioactive diterpenes. It was noted that the sugar substitution at C-20 reduced the antiproliferative activity, while the substitution of an ether group at C-9 and C-13 could increase the effect. This was evident with a stronger effect of the diterpenoids with ether group at C-13 against AGS cells compared with oxaliplatin used as a positive control. In addition, the presence of a gem-dimethyl group at C-1 and C-17 was found to increase the activity, as observed for stronger antiproliferative activities of the diterpenoids with this groups against Hep-G2 cells (IC_50_ values of 40.56 µM) and AGS cells (IC_50_ values of 22.49 µM) compared to oxaliplatin (24.26 µM) positive control [63]

Moreover, cytotoxic activities of fischernolides A–D (**311**–**314**) isolated from *E. fischeriana,* against five human cancer cell lines using the MTS method, showed that fischernolide B (**312**) [30] and fischernolide D (**314**) [30] exhibited weak cytotoxic activities with IC_50_ values of 27.30 µM (Bel-7402), 49.61 µM (HT), 20.53 µM (A549), 33.70 µM (MCF-7), 35.65 µM (HeLa) compared to cisplatin; IC_50_ = 11.9, 33.48, 12.02, 12.78, 8.65 µM respectively. The structure–activity relationship revealed that the *α*-pyrone ring had a positive effect on the activities of fischernolide D (**314**), compared with fischernolide A (**311**), B (**312**), and C (**313**) [103], with the *α*-furanone ring.

In related studies, previously isolated compounds; 12-deoxyphorbol esters, 12-deoxyphorbol-13-acetate (prostratin), 12-deoxyphorbol-13-hexadecanoate, and 12-deoxyphorbol-13-(9*Z*)-octadecanoate-20-acetate from *E. fischeriana* were evaluated for their cytotoxicity against Ramos B cells. The results showed that 12-deoxyphorbol 13-hexadecanoate, having a long acyl chain at C-13 and a free hydroxy at C-20, exhibited promising cytotoxic activity against Ramos B cells with an IC_50_ value of 0.0051 μg/mL. The findings suggested that the presence of saturated aliphatic acyl group at C-13, and a carbonyl at C-3 and free hydroxyl at C-20, were important to the cytotoxic activity against Ramos B cells of these compounds [58]. Furthermore, evaluation of the cytotoxic activities of 6*α*,7*α*-epoxy-5*β*-hydroxyphorbol ester isolated from *Excoecaria acerifolia* of *Euphorbiaceae* family against five cancer cell lines (HL-60, SMMC-7721, A-549, MCF-7, and SW480) showed significant IC_50_ values in the range of 7.62−10.87 μg/mL [58]. From the findings, it was inferred that the type of aliphatic long-chain acyl group at C-12 or C-13, a trans-fused A/B ring system, a 6,7-olefinic group, and free C-20 hydroxyl, was important to the anticancer activities of these diterpenes. Furthermore, it was evident that the main active groups present in tigliane type diterpenes with promising anticancer activities were generally like those in related diterpenes displaying tumor-promoting activities [110]. The type and nature of the long-chain acyl groups are important to their anticancer activities. In general, the high activities of these diterpenes toward the cancer cells were attributed to the 6,7-olefinic, 3-carbonyl and the acyl groups attached to the skeleton [58].

Chemical investigation of *E. stracheyi* root extracts resulted in the isolation of lathyrane and ingenane-type diterpenoids. All the isolated diterpenoids were assayed for in vitro anticancer activities against human stomach cancer cell lines (HGC-27), human leukemia cells (MV4-11), human lung carcinoma (H460), human ovarian carcinoma (Skvo3), and a human-murine cell line lymphocyte (BaF3). *3β*, 20-diacetoxy-5*β*-deca-2′′*E*, 4′′*E*, 6′′*E*-trien-4*β*-hydroxyl-1-one (euphstrachenol C) (**89**) [32] and 20-*O*-acetyl-[3-*O*-(2′*E*, 4′*Z*)-decadienoyl]-ingenol (**91**) [32], displayed significant cytotoxic activities against MV4-11cell line, with IC_50_ values of 5.92 μM and 3.48 μM, respectively, using taxol as control with an IC_50_ of 0.015 μM [32]. 

In addition, euphstrachenol A (**249**), euphstrachenol B (**250**), jolkinol B (**253**), jolkinol C (**255**), jolkinoate (**257**) [32], and 3-*O*-(2′*E*, 4′*Z*)-decadienoylingenol (**92**), exhibited modest activities against MV4-11 cell lines, with IC_50_ values ranging from 7.92 μM to 15.37 μM, compared to taxol (IC_50_ of 0.055 μM) as a control. The activities of these compounds were found to be stronger against MV4-11 compared to HGC-27 cells. Moreover, the isolated diterpenes (**249**–**261**) displayed significant anticancer activities against ovarian carcinoma (Skvo3) and lung carcinoma (H460) cell lines, with IC_50_ values less than 50.00 μM, compared to taxol [32]. Based on the findings it was concluded that the isolated compounds showing selective cytotoxicity could be promising lead compounds for the discovery of anticancer agents.

Meng et al. [65] examined the antiproliferative activities of ingenane and jatrophane diterpenoids, isolated from *E. kansui* against human hepatoma cancer cells (Hep-G2), human breast cancer cells (MCF-7), and human prostate cancer cells (DU145), employing the cell counting kit-8 (CCK-8) assay technique. The results showed that all the jatrophane and ingenane diterpenoids exhibited significant inhibitory activities on the cell proliferation of all the three cancer cells tested against. The IC_50_ values were 30.48 μM, 6.29 μM, 4.19 μM, and 26.05 μM for 20-*O*-(2′*E*, 4′*Z*-decadienoyl) ingenol (**97**) and kansuijatrophanol D (**180**) [65], against MCF-7 and DU145 cells respectively. As regards the Hep-G2 cells, the recorded IC_50_ values were 9.47 μM for kansuijatrophanol C (**176**), and 29.16 μM for kansuingenol B (**178**).

The structure–activity relationship suggested that jatrophane diterpenoids having 11,12-diol groups showed significant activities. For instance, in kansuijatrophanol A (**177**) (MCF-7; IC_50_ = 21.64 µM, Hep-G2; IC_50_ = 20.19 µM, DU145; IC_50_ = 7.21 µM) and kansuijatrophanol B (**178**) (MCF-7; IC_50_ = 15.25 µM, Hep-G2; IC_50_ = 13.24 µM, DU145; IC_50_ = 7.24 µM) [65] diterpenoids, the presence of bioactive functional group, 11, 12-diol group, had a positive effect. In addition, kansuijatrophanol C (**179**) (MCF-7; IC_50_ = 11.25 µM, Hep-G2; IC_50_ = 9.47 µM, DU145; IC_50_ = 8.29 µM) with 3,4-(methylenedioxy) cinnamyl group and kansuijatrophanol D (**180**) (MCF-7; IC_50_ = 6.29 µM, Hep-G2; IC_50_ = 10.07 µM, DU145; IC_50_ = 4.19 µM) with 3,4-(methylenedioxy) cinnamyl groups exhibited the highest activities. This further suggested that the 3,4-(methylenedioxy) cinnamyl group could be responsible for the bioactive activities [65]. These observations agreed with Kulig et al.’s [111] assertion that vicinal diol groups contribute significantly to the bioactivities of naturally occurring compounds possessing them. Other studies on the structure–activity relationship of jatrophane diterpenoids showed that substitution of a benzoate at C-8 and C-9 are favorable, while isobutanoyloxy group substitution at C-3 increased the observable effects on human lymphocytes deoxyribonucleic acid (DNA) [112]. 

Structural modification of these constituents by esterification of hydroxyl groups revealed that the 5-*O*-acetyl derivative presented triglyceride-lowering abilities with an EC_50_ value of 0.61 μM. Structure–activity relationships showed that the trans-fused 5/7/6 ring system occurring in an angular shape was relevant to these activities [37]. In addition, the presence of a cyclopropane ring, an isopropyl substituent, and cyclobutane ring on these diterpenes did not have an effect. A nicotinoyl group at C-3 was also found not to be favourable, as derivatives with this functionality recorded poor activities. Equally, the availability of a free hydroxyl group at C-8 was found to be beneficial to the activity of these compounds, while acylation of 8-OH resulted in decreased activities. Tigliane diterpenoid, 12-*O*-benzoyl-13-*O*-[2-methylpropanoyl]-4,20-dideoxy-5-hydroxyphorbol, an acetylated derivative of phorbol exhibited promising lipid-lowering activity, with an EC_50_ value of 0.32 μM and selectivity index of IC_50_/EC_50_ > 312. The SAR studies showed that phorbol derivatives, bearing a trans-fused 5/7 ring system, presented significant activities compared to those possessing a cis-fused system, indicating that the trans-fused system of 5/7 ring was beneficial or had a positive effect on the activities of tigliane diterpenes [37]. 

Phytochemical analysis of aerial extracts of *E. helioscopia* afforded three undescribed jatrophane diterpenoids named euphoheliphane A (**181**), euphoheliphane B (**182**), and euphoheliphane C (**187**) [50]. Euphoesulatin A–C (**184**–**186**), from *E. esula,* exhibited antiosteoporotic activity on BMM cells. Euphoesulatin A (**184**), euphoesulatin F–G (**189**–**190**) showed significant activities with IC_50_ values of 1.20, 6.10, and 10 µM respectively compared to receptor activator of nuclear factor kappa B ligand (RANKL) control [100]. Other euphoesulatins diterpenes displayed some antiosteoporotic activities, while some, like esulone B (**202**), showed no activity, as demonstrated in Table 2. The in vitro cytotoxicity studies of the compounds showed weak anticancer activities against cellossaurus cell lines (OS-RC-2), cellossaurus cell lines (Ketr-3), human kidney cell lines (769-P), cellossaurus cell lines (G401), human cell lines (GRC-1 and ACHN) with IC_50_ values less than 50.00 µM compared to the positive control, doxorubicin [50]. As well, euphoractone (**14**) [59] displayed potent inhibition activities against human cellasaurus cell lines (H23 and H460) with IC_50_ values of 21.07 mmol/L and 20.91 mmol/L, respectively, as compared to the positive control, cisplatin [59].

In a related study, Zolfaghari et al. [113] evaluated the potential cytotoxic activities of previously described cyclomyrsinanes and premyrsinane against EJ-138 bladder carcinoma and Jurkat T-leukaemia cell lines in vitro. Most of the tested compounds showed promising activities against EJ-138 (A) and Jurkat T cells (B), with IC_50_ values ranging from 33.31–15.3 µM against EJ-138 and 21.10–12.3 µM against Jurkat T cells, using doxorubicin as a positive control. The structure–activity relationship of the cyclomyrsinanes diterpenes revealed that their activities were modulated by the position of the substituents. In particular, substituents at C-8 had a positive influence and the activity increased with the length of the acyl chain (MeiPe > MeBu > iBu) increased [113].

### 6.2. Multidrug Resistance Activities

All diterpenoids isolated from *E. royleana* were evaluated for their chemoreversal activities against multidrug-resistant (MDR) liver cancer cells with doxorubicin (Hep-G2/DOX). All the compounds recorded weak cytotoxicity with IC_50_ of less than 50.00 μM against liver cancer cell lines (Hep-G2) and Hep-G2/DOX cell lines, using verapamil (Vrp; 10.65 μM) and tariquidar (Tar; 2.31 μM) as positive controls [33]. The cell viability of the compounds was evaluated, by adding 10.00 μM of the compound under test to 50.00 μM of doxorubicin (DOX) in Hep-G2/DOX with tariquidar (Tar) and verapamil (Vrp) as positive controls. From the results, all lathyranes type diterpenoids, ingol-3,7,12-triacetate-8-benzoate (**262**), ingol-3,8,12-triacetate-7-tiglate (**263**), 3,7,12-*O*-triacetyl-8-*O*-(2-methylbutanoyl)-ingol (**264**), euphorantins M (**265**), 8-*O*-methyl-ingol-3,12-diacetate-7-benzoate (**266**), 3,8,12-*O*-triacetylingol-7-benzoate (**268**) 8-*O*-methylingol-3,8,12-triacetate-7-angelate (**269**) and 3,12-diacetyl-8-benzoylingol (**270**), showed comparable chemoreversal activities as compared to positive control verapamil (Vrp; 10.65 μM) drug. Combinations of the diterpenoids with varying concentrations of doxorubicin (DOX) to obtain actual reversal abilities were further investigated. It was observed that among the lathyranes, ingol-3,7,12-triacetate-8-benzoate (**262**) (IC_50_ = 4.76 µM, dox; 499.88 µM) exhibited potent activities. This compound was suggested as a multidrug (MDR) modulator, as it improved the anticancer efficacy at 10.00 μM, as compared to verapamil (Vrp), with a reversal fold of 46.92 μM. With cognizance of the fact that expression of P-glycoprotein (P-gp) is the basis for multidrug mechanisms, exprimentatoin expressing P-gp in Hep-G2/DOX cells was further conducted. The results showed a significantly high expression of P-gp in the MDR cell line, compared to the parental cell line [33]. Hence, it was deduced that the multidrug (MDR) mechanisms of the lathyranes diterpenoids could be related to the modulation of the P-glycoproteins (P-gp) by down-regulation of protein expression or by blocking of their functions. It was also found that all the isolated diterpenoids inhibited the transport activities of P-glycoproteins (P-gp), rather than its expression, when tested for their effects on the expression of P-glycoproteins (P-gp) in cancer cells with doxorubicin (Hep-G2/DOX) [33]. 

Evaluation of multidrug resistance (MDR) reversal ability of isolated ingol diterpenoids from *E. marginata* against cancer cell line Hep-G2/ADR (Pgp-dependent) showed no significant cytotoxicity activities, with IC_50_ values of less than 50 μM, compared to anticancer drug adriamycin (ADR) as the positive control. Euphornans A–N (**142**–**155**) [41] showed greater reversal activities compared to verapamil, the positive control. Euphornans, K (**152**), N (**155**), and R (**159**) [41], recorded better activities than tariquidar (IC_50_ > 25 µM at 5 µM), using adriamycin as a control, and were further investigated for dose–effect relationships. The compounds (Euphornans; K (**152**), N (**155**), and R (**159**)), exhibited better dose-dependent activities and were found to reverse the sensitivity of adriamycin, the cancer drug, to 20-fold, at a concentration of 5.00 μM. In P-gp modulation-mechanism analysis, it was further established that the compounds reverse the sensitivities of multidrug (MDR) cancer cell lines by the inhibition of the P-glycoprotein (P-gp) [41].

Due to the various substitutions patterns in isolated ingol-type diterpenoids, structure–activity relationships were investigated. It was established that acetylation of the hydroxyl group at C-3 and C-8 improved the anticancer activities. In particular, the acylation of hydroxyl groups (OH-3 and OH-8) improved the activity, as shown in ingol-3,7,12-triacetate-8-benzoate (**262**) (IC_50_ = 4.76 µM), ingol-3,8,12-triacetate-7-tiglate (**263**) (IC_50_ = 27.29 µM), euphorantins M (**265**) (IC_50_ = 20.81 µM), 8-*O*-methyl-ingol-3,12-diacetate-7-benzoate (**267**) (IC_50_ > 100 µM), 3,8,12-*O*-triacetylingol-7-benzoate (**268**) (IC_50_ = 11.18 µM), 8-*O*-methylingol-3,8,12-triacetate-7-angelate (**269**) (IC_50_=17.83 µM), and 3,12-diacetyl-8-benzoylingol (**270**) (IC_50_ = 17.82 µM), compared to doxorubicin (IC_50_ = 499.88 µM) [33]. Furthermore, the diterpenoids bearing a benzoyl group at C-7 and C-8 recorded the highest activities compared to those with OMeBu, angeloyl and tigloyl groups. This was evident in compounds 8-*O*-methyl-ingol-3,12-diacetate-7-benzoate (**267**) compared to 8-*O*-methylingol-3,8,12-triacetate-7-angelate (**269**), 3,8,12-*O*-triacetylingol-7-benzoate (**268**) compared to ingol-3,8,12-triacetate-7-tiglate (**263**), ingol-3,7,12-triacetate-8-benzoate (**262**) compared to 3,7,12-O-triacetyl-8-*O*-(2-methylbutanoyl)-ingol (**264**) and in 3,12-diacetyl-8-benzoylingol (**270**) compared to 3,12-di-*O*-acetyl-8-*O*-tigloyl-ingol (**266**) [33]. 

Molecular mechanisms of these diterpenoids and P-glycoprotein (P-gp) were further explored by in silico analysis. All the compounds were found to dock well in the transmembrane domain (TMD) of P-gp. Formations of three hydrogen bonds between 8-OBz and Gln-990, and Tyr310 and between 3-OAc and Tyr953 were observed. It was also found that their core structures formed hydrophobic forces between the aromatic moieties and hydrophobic residues of the transmembrane domain (TMD) pocket that favoured the binding. It is this binding that was used to explain the structure–activity relationships (SARs) of the isolated lathyranes diterpenoids [33]. Equally, molecular docking experiments of the lathyrane diterpenoids presented lower binding energies, compared to the positive controls, adriamycin, and verapamil. The data further established that the isolated lathyrane diterpenes could act as a substrate of high-affinity, P-glycoprotein (P-gp) which is effluxed with its monomer to reverse multidrug resistance (MDR). Hence, the MDR-reversal activities of these diterpenes were postulated to occur via two strategies. The main strategy was by maintaining the chemotherapeutic drug concentrations as high as possible, by suppressing overexpression of P-glycoprotein (P-gp) in the MDR cells. The second strategy involved reducing the efflux of P-glycoprotein (P-gp)-regulated drugs or chemotherapeutics. In this model, compounds (diterpenes) were found to replace the chemotherapeutic drugs as the P-glycoprotein (P-gp) efflux substrate. The findings showed that lathyrane diterpenoids are good (P-gp) efflux substrates with high affinity. Hence, they were praised for their ability to suppress the overproduction of (P-gp) in multidrug cell strains and could be potential candidates for cancer agents [106]. Chemical modification of the diterpenoids presents promising multidrug resistance modulators. 

In summary, the bioactivities of jatrophane and lathyrane diterpenoids can be increased by acylation and esterification of the hydroxyls groups, which subsequently improves the hydrophobicity with the P-glycoprotein (P-gp) inhibitor. For instance, esterification of the hydroxyl group at C-3 and C-8 were vital for the activities of lathyranes diterpenoids, as the presence of hydroxyl groups was found to decrease activity due to the interference of hydrogen bonds. Likewise, diterpenoids having a benzoyl group at C-7 or C-8 displayed higher activities compared to those with tigloyl, angeloyl, and MeBu groups, as observed in some diterpenoids. This could be due to the interaction of the π electrons in the phenyl ring of the 8-OBz with the hydrophobic pockets favoring the binding [33]. Similar observations were made in ingol diterpenoids, isolated from *E. marginata*. Esterification of the C-OH by acylation was found to enhance the activities, as observed in euphornan B (**143**) and G (**148**), euphornans J (**151**), and O (**156**), euphornans K (**152**) and P (**157**) [41]. Acylation of the C-7 hydroxyl group was found to reduce activity, as observed in euphornan F (**147**) and B (**143**), as well in euphornan N (**155**) and J (**151**). In contrast, the substitution of OH-7 with benzoyl displayed better activity than when substituted with acetyl, as observed in euphornan K (**152**) and euphornan I (**150**) [41]. Yet, the replacement of nicotinoyl by benzoyl at the hydroxylated C-9 increased activity remarkably [41]. 

Evaluation of isolated compounds from *E. lathyris* for their reversing multidrug (MDR) activity against hepatocellular carcinoma (Hep-G2/ADR) cells showed that 5,15,17-*O*-tri-acetyl-3-*O*-nicotinoyllathyol-6,12-diene -14-one (**279**) displayed MDR reversal of Hep-G2/ADR at 20 μM with IC_50_ value of 37.25 μM, compared to verapamil (Vrp), with IC_50_ value of 51.95 μM. The mechanism of MDR reversal by lathyrane diterpenes was investigated. The results showed that the most potent lathyrane diterpenoid was able to facilitate the time-dependent build-up of intracellular adriamycin in hepatocellular carcinoma (Hep-G2/ADR) cells. It was found to activate the P-glycoprotein (P-gp) in a dose-dependent manner [106]. 

Likewise, evaluation of the multidrug resistance (MDR) activity of jatrophane diterpenoids, from *E. esula,* against cancer cell lines that are dependent on P-glycoprotein (Hep-G2/ADR), showed comparable activities to adriamycin (ADR), the positive control drug. Most compounds did not show obvious cytotoxicity in Hep-G2/ ADR cell lines, with IC_50_ values less than 50.00 μM. However, euphoresulane H (**212**) [59] was the best multidrug resistance (MDR), modulator with IC_50_ of 165.30 µM, compared to ADR (IC_50_ of 284.50 µM), and was established to further enhance the anticancer activities of adriamycin by 33-fold at 5.00 μM. Hence, euphoresulane H (**212**) was further studied for a dose-effect dependence and ireported good dose-dependent activities, as it enhanced the activities of adriamycin (ADR) by 33-fold at 5 μM [59]. The cytotoxic evaluation of jatrophane diterpenoids isolated from the acetone extracts of *E. glomerulans* on multidrug-resistant breast cancer cells (MCF-7/ADR) was found to overexpress the P-glycoprotein (P-gp) with varying chemoreversal abilities and with reduced cytotoxicity activity. Euphoglomeruphane K (**233**) and L (**234**) showed better MDR reversal activity, with IC_50_ values of 5.00 μM and 5.10 μM, respectively, compared to verapamil, the positive control, with IC_50_ value of 4.70 μM [29].

The different substitutions patterns of the isolated jatrophane diterpenoids formed the basis for further evaluation of their structure–activity relationships. It was established that the existence of a keto carbonyl at C-9 in euphoresulane J–M (**214-217**) (IC_50_ > 100 µM; ADR; IC_50_ = 284.50 µM) [59] adversely affected their activities. It was noted that the existence of the acetoxy group at C-15 resulted in better activities in compounds bearing the acetoxy group than those with free hydroxyl at this position. It was also established that the acylated group at C-9 enhanced activity. Nonetheless, compounds with 9-OBz showed better activities than those with 9-OAc, as observed in euphoresulane F (**210**) (IC_50_ > 50 µM; ADR; IC_50_ = 284.50 µM) and euphoresulane E (**209**) (IC_50_ > 100 µM; ADR; IC_50_ = 284.50 µM) and in euphoresulane H (**212**) (IC_50_ = 165.30 µM, ADR; IC_50_ = 284.50 µM) and euphoresulane D (**208**) (IC_50_ > 100 µM; ADR; IC_50_ = 284.50 µM) [59]. In addition, jatrophane, possessing hydrogen at C-2, showed significant activity over those bearing an acetoxy group at the same position, as observed in euphoresulane B (**206**) (IC_50_ > 25 µM, ADR; IC_50_ = 284.50 µM) and euphoresulane A (**205**) (IC_50_ > 100 µM, ADR; IC_50_ = 284.50 µM) and in euphoresulane F (**210**) and euphoresulane G (**211**). Taken together, it was deduced that the acyloxy substitution at C-9 in jatrophane is essential to its activity, while the existence of C-OH enhances activity [59]. 

**Figure 17:**
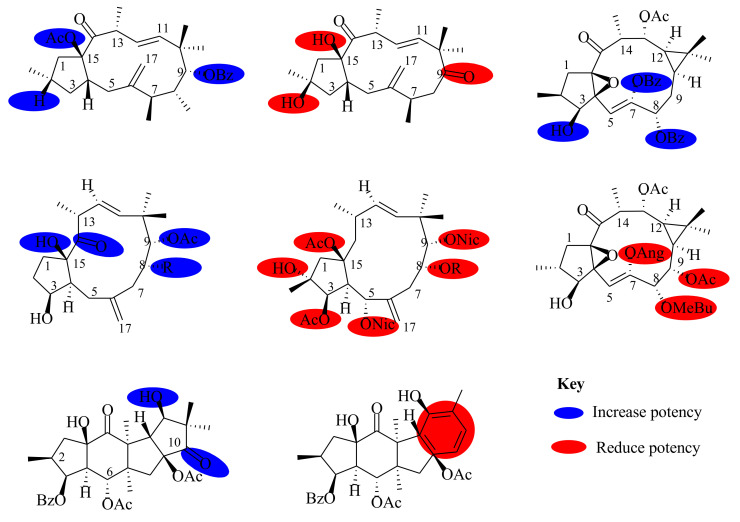
Key pharmacophoric elements of jatrophanes and modified jatrophanes.

The biological evaluation of these jatrophanes and modified jatrophanes showed the significance of substitutions at C-3, C-6, and C-15, in addition to the configuration of the hydroxyl group. For instance, substitution at C-6 was found to affect the inhibitory activities in a way that was dependent on the position of the free hydroxyl group, while substitution of benzoyl and propyl at C-9 and C-3 reported positive inhibitory activities. Furthermore, jatrophanes possessing acetyl at C-8 and nicotinyl at C-9 reported significantly higher activities. These observations showed that jatrophanes and the modified jatrophanes possess common pharmacophoric elements that affect their activities as in Figure 17 [114,115].

Previously undescribed diterpenoids isolated from *E. pekinensis* were evaluated for their cytotoxicity activities against human prostate cancer (C4-2B), enzalutamide-resistant C4-2B cell line (C4-2B/ENZR), and human breast cancer cells (MDA-MB-231). All the tested compounds exhibited significant cytotoxic activities against C4-2B/ENZR and C4-2B cell lines, with most of them recording IC_50_ values ranging between 14.10 µM to 34.70 µM, with low activity reported for the MDA-MB-231 cell line. Notably, euphopane A (**170**), euphopane B (**16**) and (12*β*)-2,12-dihydroxyisopimara-1,7,15-trien-3-one (**171**) displayed the most potent activity against C4-2B cell line, with IC_50_ values of 14.30 µM, 16.90 µM, and 15.30 µM, respectively, compared to doxorubicin, with IC_50_ values of 0.53, 1.06 and 0.78 µM [44]. 

### 6.3. Inhibition Activities

Examination of the inhibitory activities of euphanoids A–B (**300**–**301**) [35] atisane and *ent*-atisane diterpenoids, from *E. kansuensis,* on nitric oxide (NO) production in lipopolysaccharide (LPS)-induced RAW264.7 macrophages, showed weak activities, with IC_50_ values of less than 40 µM. Euphanoid A (**300**) and B (**301**) [35] showed significant inhibition of NO, registering IC_50_ values of 4.70 µM and 9.50 µM, respectively, compared to quercetin, a well-known NO inhibitor as the positive control with IC_50_ of 10.80 µM [35]. The evaluation of rosane diterpenoids from *E*. *ebracteolata*; ebraphenols A–D (**332**–**335**), and ebralactone A (**336**) [48] showed potent lipase inhibition activity, using lovastatin as a positive control drug, with an IC_50_ value of 0.24 µM. Ebraphenol A (**332**) exhibited the most significant effects of hydrolase lipase, with an IC_50_ value of 1.00 µM. The inhibition was found to be dose-dependent, with a calculated inhibition kinetic parameter (K*i*) of 1.80 µM [48]. Anti-inflammation activities of rosane-type diterpenes euphnerin A (**337**) and euphnerin B (**249**), isolated from stems extracts of *E. neriifolia,* showed NO inhibition in lipopolysaccharide-induced microglia cells (BV-cells), with IC_50_ values of 22.40 µM and 30.00 µM, respectively, using 2-methyl-2-thiopseudourea sulphate (SMT) as a positive control (IC_50_ value of 2.00 µM) [71]. Additionally, all the isolated *ent*-isopimarane (**42**–**53**) diterpenes from *E. neriifolia* showed no activity when tested for the inhibitory properties of nitric oxide (NO) production in lipopolysaccharide (LPS)-stimulated RAW 264.7 cells [73].

The *α*-glucosidase inhibitory activities of isolated 19-acetoxyingols from *E. saudiarabica* showed superior inhibition activity, with IC_50_ values of 7.10 μM, 8.00 μM, and 1.80 μM for saudiarabicain C (**133**), saudiarabicain D (**134**), and saudiarabicain E (**135**) [28] respectively, as compared to IC_50_ of 147.00 μM for the positive control. Saudiarabicain A (**115**) and saudiarabicain B (**116**) displayed weak inhibition activities at 150 μM, with inhibition rates of about 30.90% and 36.30%, respectively, using P-glycoprotein (IC_50_ = 0.80 µM) as a positive control. Furthermore, saudiarabicain C (**133**), saudiarabicain D (**134**), and saudiarabicain E (**135**) [28] exhibited comparable activities, as shown by the positive control valspodar. The IC_50_ values were 0.10 μM for saudiarabicain D (**134**) and 1.40 μM for saudiarabicain E (**135**), compared to 0.20 μM, for valspodar [28]. 

Evaluation of *ent*-atisane diterpenoids from *E. antiquorum* for their *α*-glucosidase inhibitory activities revealed that *ent*-3*α*-acetoxy-16*β*,17,18-trihydroxyatisane (**32**), *ent*-14[*S*],16*α*,17-trihydroxyatisan-3-one (**34**) and gallochaol C (**35**) had the highest inhibition activities, with IC_50_ values of 119.90 µM, 135.50 µM and 134.30 µM respectively, compared to acarbose, the positive control, with IC_50_ of 162.50 µM. Ent-3*α*,14,16β,17-tetrahydroxyatisane (**33**) did not show inhibitory effects (*α*-glucosidase) with IC_50_ > 200.00 µM. All compounds (**32**–**34**) displayed no cytotoxicity effect against K562, compared to acarbose (IC_50_ = 162.50 µM). It was also noted that the activities of these compounds were due to the presence of hydroxyl groups at C-16 and C-17, which are their bioactive functionalities [54]. Ent-atisane-3*β*,16*α*,17-triol (**36**), from the root extracts of *E. kansuensis,* showed no inhibition of NO (IC_50_ > 50 µM compared to quercetin (IC_50_ = 10.80 µM) [35].

Euphorin A (**37**) and euphorin B (**38**), from *E.* *antiquorum,* displayed inhibition of NO production in BV-2NO with IC_50_ value of 35.80 and 41.40 µM compared to 2-methyl-2-thiopseudourea, sulphate (SMT) (4.2 µM) [56], while diterpenes (**39**) and (**40**) from the stem extracts of *E. royleana* recorded IC_50_ > 50 µM [75]. Compounds (**56**), (**59**), from the root extracts of *E. royleana*, showed inhibition of NO in RAW264 with IC_50_ of 45.48 and 57.51 µM, respectively, compared to indomethacin (IC_50_ = 41.41 µM), while compounds (**57**), (**58**), (**60**), (**61**) and (**64**) displayed no activity 75]. Compounds (**66**) and (**67**) from *E. royleana* showed significant inhibitory activities, with IC_50_ of 32.60 and 19.30 µM, respectively, compared to 2-methyl-2-thiopseudourea, sulfate (3.7 µM) [75]. Previously, evaluation of the water fraction of *E. royleana* latex displayed dose-dependent anti-arthritic and anti-inflammatory activities in acute and chronic test models in mice and rats. Further studies showed that it reduced the migration of leukocytes and had poor inhibitory effects on the granuloma formation induced by cotton pellets. The ethyl acetate fraction on the other hand showed dose-related peripheral analgesic effects [116]. These findings support the use of *E. royleana* as an analgesic in traditional medicine. These effects could be due to the presence of *ent*-isopimaranes diterpenoids. Ebraphenol A-D (**332**–**335**) and ebralactone A (**336**), from the root extracts of *E. ebracteolate,* showed high lipase-inhibitory activity, with IC_50_ values of between 1.0 and 24 µM, compared to lovastatin positive control; IC_50_ = 0.24 µM [48]. 

Lathyrane diterpenoids from *E. lathyris* were investigated for the inhibition activities against induction of nitric oxide (NO) generation by lipopolysaccharide in murine macrophage cells (RAW264.7). Most of the diterpenoids showed significant inhibitory effects on NO production, with varying IC_50_ values of between 2.10 µM to 25.00 μM compared to dexamethasone, positive control (C_50_ = 7.9μM). (2*S*,3*S*,4*S*,5*R*,9*S*,11*R*,15*R*)-15-acetoxy-3-cinnamoyloxy-5-hydroxy-14-oxolathyra-6(17)-12*E*-diene (**302**) [103] was further evaluated for dose-dependent experiments. It was found to reduce the production of cytokines and decreasing the expression of proteins phosphorylated nuclear factor kappa light polypeptide gene enhancer in B-cells inhibitor, alpha (IκB*α*), inducible nitric oxide synthase (iNOS), and nuclear factor kappa-light-enhancer of activated B-cells (NF-κB). Based on these findings, it was concluded that this diterpenoid could be a potential anti-inflammatory agent for future studies [103]. 

In another structure–activity relationship study by Wang et al. [31], the previously isolated lathyrane diterpenoids with anti-inflammatories named euphorbia factors L_2_ (**247**), L_3_ (**248**) [31], were found to reduce the formation of inflammatory factors and decreasing the expression of nuclear factor kappa B (NF-κB). They further investigated the influence of substituted benzoic acid, cinnamic acid, and other heterocyclic acids through esterification reactions on the anti-inflammatory efficacy of the analogs. The results showed that, when the hydroxyl group on C-7 of the euphorbia factors L_3_ (**248**) was esterified, many of the yielded intermediates exhibited weaker inhibitory activities compared to the parent compound. This was an indication that the hydroxyl group on C-7 is essential in retaining the anti-inflammatory activities of euphorbia factors. However, when the hydroxyl was esterified using fatty acids like nicotinic acid and glycine, the yielded derivatives displayed better inhibition activities. While isonicotinic acid derivatives showed poor inhibition activities. This suggested that the anti-inflammatory activities of lathyranes diterpenoids could be increased by esterification [31].

This was also evident when the hydroxyl on C-5 was esterified. When the hydroxyl groups on C-3 and C-5 were esterified simultaneously, the observed activities were found to be higher. It was also established that compounds with aromatic groups exhibited high efficacy than those with aliphatic substituents [31]. Interestingly, when the substituents of the benzene were changed or when the ring was converted into a heterocyclic ring, the inhibition activities of these compounds were weakened. Also, the presence of an electron-donating group on the benzene ring was found to weaken anti-inflammatory activity more than when an electron-withdrawing moiety was attached [31]. It was further shown that lathyrane diterpenoids with an exocyclic Δ ^6(17)^ double bond presented higher inhibitory activities than those with a 5*α*, 6*β*-epoxy or Δ ^5(6)^ double bond. In addition, a substituted aromatic moiety at C-3 and nitrogen-containing aromatic substituent at C-7 were essential for retaining the inhibition of NO production [103]. Hence, it was concluded that *Euphorbia* lathyrane diterpenoids present good scaffolds for structure modification concerning drug discovery. 

All isolated euphoesulatins A–R (**184**–**201**) [100] from *E. esula* were evaluated for their inhibition abilities of receptor activators of the nuclear factor kappa B ligand (RANKL)-induced osteoclastogenesis of the macrophage cells derived in bone marrow. Strong activities were reported for euphoesulatin A (**184**), euphoesulatin D–H (**187**–**191**), euphoesulatin J (**193**), M (**196**), and O (**198**) against RANKL-induced osteoclastogenesis (BMM) cells. Specifically, euphoesulatin A (**184**), showed the best antiosteoporotic activities, with an IC_50_ value of 1.20 μM compared to the RANKL control group [100].

The structure–activity relationships (SAR), revealed that most of the euphoesulatins A–L (**184**–**193**) [100] possessing a double bond exhibited stronger activities with IC_50_ values of less than 10 μM, while some showed weaker activities with IC_50_ values of >10 μM. Substitution of hydroxyl at C-15 with an acetoxy group was found to increase the activities in euphoesulatin A (**184**) registering IC_50_ of 1.20 μM, compared to euphoesulatin B (**185**) with IC_50_ of less than 10 μM, and for euphoesulatin H (**191**), with IC_50_ value of 3.50 μM compared to euphoesulatin I (**191**), with IC_50_ value of more than 10 μM [100]. It was observed that the presence of a hydroxyl group at C-5 destroyed these activities. 

The antiosteoporotic activities of compounds having a double bond and a hydroxyl group at C-2, with identical structures other than the substituents at C-11 and C-12, showed increased activity. For instance, euphoesulatin E (**188**) compared to euphoesulatin N (**197**) ([34], and euphoesulatin H (**191**) compared to euphoesulatin M (**196**) [34]. Replacement of the Δ ^11(12)^ double bond with an epoxide resulted in increased activity. In contrast, euphoesulatins having an epoxy group and a 2-OH substituent recorded decreased or no activitys. For euphoesulatins, having a Δ^11, 12^ double bond in addition to a 2-OH functionality, either an 8-OH as in esulone B (**202**) or a 15-OH as in esulone A (**204**), resulted in no activity. This is an indication that higher numbers of hydroxyl groups does not translate to enhanced bioactivities. The SAR of the jatrophane diterpenoids supported the fact that a Δ ^11(12)^ double bond retains their activities and that the higher number of hydroxyl groups does not enhance antiosteoclastogenesis [100]. 

### 6.4. Anti-HIV Activities

Twelve *ent*-isopimarane diterpenes isolated from stem barks of *E. neriifolia* were evaluated in vitro for the anti-HIV properties in HIV-1 NL4-3 infected MT4 cells, with zidovudine (AZT) as the positive control. All the tested compounds showed significant anti-HIV activities. Eupneria J (**42**) and eurifoloid H (**24**) [69] reported potent activities, with IC_50_ values of 0.31 μg/mL and 6.70 μg/mL, respectively, while others showed insignificant activities with an IC_50_ value of fewer than 25.00 μg/mL. Further investigation of the structure–activity relationship (SAR) of the eupneria J (**42**), eupneria K (**43**), eupneria M (**45**), eupneria P (**48**), and oryzalexin F (**50**) [73] presumed from the observations that *β*-oriented hydroxyl group at C-4 could be linked to their activity. The comparative analysis of the SAR of eupneria O (**47**), eurifoloid I (**49**), and eurifoloid H (**24)** revealed that the acetoxy group at C-18 contributes to the anti-HIV activities, rather than at C-3 [69,70]. 

In another study, the phytochemical analysis of *E. lathyris* ethanol crude extracts resulted in the isolation of ingenane and lathyrane type diterpenoids. All the isolated compounds (**282**–**295**) [31] were evaluated for their anti-HIV activities against HIV-1 and MT4 cells. None of the tested compounds showed anti-HIV activities compared to zidovudine positive control, nonetheless, the ethanol crude extracts showed significant activities with an EC_50_ value of 0.33 µg/mL against the HIV-1 [31] This showed that the compounds were potent due to synergy. Analysis of isolated diterpenoids from *E. neriifolia* for ant-HIV activities revealed that *ent*-16*α*,17-dihydroxyatisan-3-one, and eurifoloid R showed potent anti-HIV-1 activities with EC_50_ values of 6.32 μg/mL and 6.45 μg/mL respectively [69,70]. In related studies, two *ent*-atisanes, including ebractenone A and bractenone B possessing a rare 2-oxopropyl moiety, displayed good antiviral activities against human rhinovirus 3, with an IC_50_ value of 25.27 μM [117]. 

Evaluation of tigliane diterpenes 16-angeloyloxy-13*α* -isobutanoyloxy-4*β*, 9*α*-dihydroxytiglia-1, 6- dien-3-one (**344**) and 20-acetoxy-13*α*-isobutanoyloxy-4*β*, 9*α*, 16-trihydroxytiglia-1, 6-dien-3-one (**345**) from *E. grandicornis* for their protein kinase C activation and platelet stimulation abilities revealed that these compounds enhance the platelet stimulation [52]. As well, phorbol esters prostratin 20-*O*-(6′-acetate)-*β*-D-glucopyranoside (**339**) and fischeroside A (**340**) from *E. fischeriana* showed promising cytotoxic activities against AGS (IC_50_ = 27.97 µM), and Hep-G2 (IC_50_ = 17.59 µM) using oxaliplatin as the positive control with IC_50_ value of 17.06 and 24.26 µM for AGS and Hep-G2 respectively [63]. Even though the anti-HIV activities of these diterpenes were not evaluated, previous studies on related phorbol esters from *E. fischeriana* like deoxyphorbol-13,20-diacetate showed promising anti-HIV-1 activity with an EC_50_ value of 0.003 μM, whereas prostratin displayed the strongest anti-HIV-1 activity, with an EC_50_ value of 0.00006 μM compared to zidovudine, used as a positive control. Furthermore, the introduction of an *O*-acetyl or glucopyranosyl moiety at C-20 of the prostratin reduced the anti-HIV-1 activity significantly. The compound did not exhibit tumour-promoting effects and caused growth inhibition in all of the cell lines tested. The results, therefore, suggested that a long chain was necessary for 12-deoxyphorbol to show anti-HIV activity, and that the presence of a long chain was relevant for these compounds to show anticancer effects [58]. Anti-HIV activities of secoeuphoractin (**379**) and euphorbactin (**380**) from *E. micractina* using zidovudine as positive control showed promising activities [67]. Compound (**379**) had an IC_50_ value of 1.76 µmol/L compared to zidovudine (0.005 µmol/L, positive control), while (**380**) had an IC_50_ value of 28.6 µM compared to zidovudine (0.005 µM, positive control) [67]. 

### 6.5. Anti-Influenza

All the *ent*-isopimarane diterpenes isolated from stem barks of *E. neriifolia* were evaluated for their anti-influenza virus activity on Madin–Darby canine kidney (MDCK) cells, with nucleozin as a positive control. From the findings, *ent*-isopimara-8(14),15-dien-3*β*,12*β*-diol (**52**) exhibited the highest activity with IC_50_ at a concentration of 3.86 μg/mL compared to the nucleozin with IC_50_ at 0.37 μg/mL [73]. 

### 6.6. Melanin Synthesis

Biological studies on six new lathyrane, *ent*-abietane and known ingenols diterpenoids, from *E. antiquorum*, revealed that ingenol diterpenoids had better activities on melanin synthesis. Among them, euphonoid A (**117**), euphorantin I (**123**), and euphorantin J (**127**) [34] displayed better inhibition abilities of 124.38%, 203.11%, and 177.43% as compared to the positive control (8-MOP; 124.38%) at 50 µM. The ingenol diterpenoids were found to be almost twice better than the positive control, with euphorantin I (**123**), showing the highest value at 203.10% against B16 cells. It was therefore deduced that this compound could be a promising agent for the treatment of vitiligo diseases [34]. 

### 6.7. Antibacterial and Antimalarial Activities

The findings from the published records showed that only two publications reported the antibacterial and antimalarial activities of isolated diterpenes. In comparison to the cytotoxic and anti-inflammatory studies, the antimalarial and antibacterial activities were not significant. *Ent*-abietane diterpenoids isolated from roots extracts of *E. wallichii* were evaluated for antimicrobial activity against six Gram-positive bacteria, including *Eorynebacterium* (T25-17), *Enterococcus* species (8152), *Enterococcus faecalis* (C159-6), and Gram-negative bacteria including *Citrobacter freundii* (11041), *Acinetobacter baumanii* (9010) and *A. baumanii* (9011) using gentamicin as a positive control. 11*β*-hydroxy-14-oxo-17-al-ent-abieta-8(9), 13(15)-dien-16,12*β*-olide (**11**), 11*β*, 17-dihydroxy-12-methoxy-ent-abieta-8(14), 13(15)-dien-16,12A-olide (**12**) and 14A-hydroxy-17-al-entabieta-7(8), 1 1(12), 13(15)-trien-16, 12-olide (**13**) were found to exhibit significant antimicrobial activities against the three Gram-positive bacteria, with MIC value of 60.00 µg/mL, but they displayed no antimicrobial activity against the Gram-negative bacteria, as compared to gentamicin as the control [47]. Paraliane and presegetane diterpenes euphorbesulin D (353), euphorbesulin A–C (**366**–**368**), and euphorbesulin E (**374**) from *E. esula* displayed antimalarial activity (IC_50_ > 50 µM) compared to artemisinin (7.01 µM) as a positive control [49]. 

## 7. Conclusions and Prospects

In recent years, there has been growing interest in *Euphorbia* species to discover new diterpenes with promising biological activities and which possess an intriguing structural framework. Due to the emergence of new structurally diverse *Euphorbia* diterpenes with a wide range of pharmacological activities, it was remarkable to review the latest information on their isolation, structures, biological activities, and the structure–activity relationship. In the course of our survey, it was established that over 350 new diterpenes were isolated for the first time in roots, stems, seeds, stem barks, and whole plant of *Euphorbia* species, each bearing different skeletal structures. Particularly, jatrophanes, lathyranes, and ingenanes possessing structurally unique polyoxygenated derivatives were predominant in most species. These diterpenes are promising compounds for multidrug resistance reversal abilities and showed the ability to act as anti-inflammatory agents both in vivo and in vitro. These properties might open new insights and perspectives in designing and developing new anti-inflammatory drugs. It is also noteworthy that, some diterpenoids with unusual skeletal frameworks like meroterpenoids, were reported for the first time in *Euphorbia* species with promising cytotoxic, antibacterial, anti-HIV, anti-influenza, multidrug resistance reversal abilities and anti-inflammatory activities. Specifically, jatrophanes and lathyranes diterpenoids were found to inhibit the P-glycoprotein thus inducing multidrug resistance-reversal abilities. The anticancer activities of these diterpenes were largely investigated. Conversely, SAR studies on the isolated diterpenes and their analogs revealed the significance of hydroxyl functionality within the structures. Esterification of this functionality was shown to enhance the activities in some analogs and lowered or showed no effect in others. For instance, jatrophanes diterpenoids having 11,12-diol groups showed significant activity, while mysrinanes possessing trans-fused 5/7/6 ring system occurring in an angular shape was relevant to their activity; as well, the free hydroxyl group at C-8 was found to be beneficial to the activity of these compounds. It was established that acetylation of the hydroxyl group at C-3 and C-8 in ingol and lathyranes type diterpenoids improved activity. The SAR studies of these diterpenes are essential as they can help to synthesize and discover lead compounds with low toxicity, good solubility, and high potency. It is significant to note that diterpenoids possessing unusual skeletal structures showed significant cytotoxic activities. It is observed that, despite the wide isolation of these diterpenoids, there is little publication on their total or semi-synthesis, that isolation from medicinal plants remains the only source of obtaining them notwithstanding, the unique skeletal structures and frameworks exhibited by *Euphorbia* diterpenes that can be precursors in synthetic endeavors to construct new derivatives with improved activities. Furthermore, few studies on these diterpenoids have reached clinical trials and for the few in vitro studies conducted, emphasis was focused on only limited pharmacological studies. It is also surprising to note that, despite tigliane (phorbol esters) reporting better activity, they have been isolated only in few species of the genus recently. This could be due to their complex nature that hinders their isolation and identification. In addition, little has been investigated to evaluate the toxicities of these diterpenes and their mechanisms of action. Therefore, to obtain more comprehensive information about the isolated diterpenes, there is a need for further studies to determine their mode and mechanisms of action. Also, more attention should be directed to their latex and water-soluble components, as limited study on these extracts is reported. It is also fascinating to note that over 380 new diterpenes were isolated in slightly over 30 *Euphorbia* species of more than 2000 species in the genus. This shows the structural diversity of *Euphorbia* diterpenes yet to be isolated. These diterpenes will give insights and understanding of the taxonomic relationship of *Euphorbia* species, and their chemotaxonomic significance. Hence, the current review shows the potential of the genus *Euphorbia* as a promising source of new bioactive compounds that will provide possible lead compounds for pharmaceutical applications, such as anticancer and anti-inflammatory agents.

## Figures and Tables

**Table 1:** Occurrence of *Euphorbia* diterpenes and their reported biological studies.

Species Name	Class (*n* = Number of Isolated Compounds)	Biological Study	Reference
*E. aellenii*	jatrophane (*n* = 2)	Antiproliferative	[39]
*E. antiquorum*	*ent*-abietane *n* = 1), *ent*-atisane (*n* = 7), ingenol (*n* = 1), ingol (*n* = 16), ingol (*n* = 4), lathyrane (*n* = 3)	Melanin synthesis activity, inhibitory (*α*-glucosidase), inhibitory (NO production)	[34,54,55,56]
*E. dracunculoides*	tigliane (*n* = 1), myrsinol (*n* = 2)	Cytotoxic	[57,58]
*E. ebracteolata*	rosane (*n* = 5)	Lipase inhibitory	[48]
*E. esula*	jatrophane (*n* = 41)	Antimalarial	[34,59]
*E. fischeriana*	*ent*-abietane (*n* = 4), meroterpenoid (*n* = 5), tigliane (*n* = 5)	Cytotoxic	[30,60,61,62,63]
*E. gaditana*	gaditanone (*n* = 1)	Not evaluated	[27]
*E. glomerulans*	jatrophane (*n* = 17)	MDR-chemoreversal	[29]
*E. grandicornis*	tigliane (*n* = 2)	Protein kinase C activation and platelet stimulation abilities	[52]
*E. helioscopia*	jatrophane (*n* = 10)	Cytotoxic, inhibitory (nitric oxide (NO)	[50,64]
*E. hylonoma*	*ent*-isopimarane (*n* = 9), *ent*-rosane (*n* = 1)	Inhibitory (NO)	[45]
*E. kansuensis*	atisane (*n* = 1), *ent*-atisane (*n* = 1), *ent*-labdane (*n* = 2), ingenane (*n* = 1), kaurane (*n* = 1)	Cytotoxic, inhibition of NO	[35,36]
*E. kansui*	ingenane (*n* = 15), jatrophane (*n* = 8)	Cytotoxic effect, antiproliferative	[53,65]
*E. kopetdaghi*	Other	Cytotoxic	[25]
*E. lathyris*	ingenane (*n* = 1), lathyrane (*n* = 8), tigliane (*n* = 1)	Inhibitory of NO	[31,66]
*E. marginata*	ingol (*n* = 20),	Multidrug reversal activity	[41]
*E. micractina*	jatrophane (*n* = 2)	Anti-HIV-1 replication ability	[67]
*E. milii*	*ent*-rosane (*n* = 16)	Inhibitory (anti-EBV lytic replication	[46]
*E.* *neriifolia*	abietane *n* = 2), *ent*-abietane (*n* = 9), *ent*-isopimarane (*n* = 12), *ent*-rosane (*n* = 1), ingol (*n* = 5), rosane (*n* = 2)	Anti-HIV-1, antiangiogenic activity, anti-influenza, NO inhibitory effects	[42,68,69,70,71,72,73]
*E. pekinensis*	cembrane (*n* = 1), *ent*-abietane (*n* = 1), isopimarane (*n* = 4)	Cytotoxic	[44]
*E. peplus*	abietane (*n* = 2), *ent*-abietane (*n* = 1), *ent*-labdane (*n* = 1), paralianone (*n* = 3), paraliane (*n* = 8), pepluane (*n* = 7)	Cytotoxic, inhibition of LPS-stimulated NO production	[38,72]
*E. pilosa*	jatrophane (*n* = 2)	Cytotoxic	[40]
*E. prolifera*	mysrinane (*n* = 7), myrsinol (*n* = 4)	Lipid-lowering activity	[37,74]
*E. resinifera*	Ingol (*n* = 3),	Cytotoxic	[43]
*E. royleana*	cembrane (*n* = 2), *ent*-atisane (*n* = 4), *ent*-isopimarane (*n* = 2), *ent*-kaurane (*n* = 3), ingenane (*n* = 3), ingol (*n* = 5), lathyrane (*n* = 35)	MDR-chemoreversal, chemoreversal combination abilities, inhibitory (NO production)	[33,43,75]
*E. sanctae-catharinae*	premyrsinane (*n* = 3)	Not evaluated	[26]
*E. saudiarabica*	ingol (*n* = 2), ingenol (*n* = 2)	Inhibitory (*α*-glucosidase)	[28]
*E. stracheyi*	abietane (*n* = 2), *ent*-atisane (*n* = 2), ingenane (*n* = 4), lathyrane (*n* = 12), pimarane (*n* = 3),	Cytotoxic	[32]
*E. wallichii*	*ent*-abietane (*n* = 3)	Antibacterial	[47]

## Data Availability

Not applicable.

## Abbreviations

AA	Arachidonic acid
A-549	Human lung cancer cells
AGS	Human gastric cancer cell lines
BaF3	Human murine cell line lymphocyte
BMM	Osteoclastogenesis cells
C4-2B	Human prostate cancer
C4-2B/ENZR	Enzalutamide-resistant C4-2B cell line
DU145	Human prostate cancer cells
GGPP	Geranyl geranyl pyrophosphate
H460	Human lung carcinoma
Hep-G2	Human liver cancer cells
Hep-G2/ADR	Hepatocellular carcinoma
HGC-27	Human stomach cancer cell lines
HL-60	Human leukemia cells
MDA-MB-231	Human breast cancer cells
MDCK	Madin-Darby canine kidney cells
MV4-11	Human leukemia cells
RANKL	Receptor activator of nuclear factor kappa B ligand
RAW264.7	Macrophages cells
Skvo3	Human ovarian carcinoma
SMMC-7721	Liver cancer cells
SW480	Colon cancer cells

## References

[1] De Montellano B.O. (1975). Empirical Aztec medicine. Science.

[2] Hooper M. (2002). Major Herbs of Ayurveda.

[3] Hargreaves B.J. (1991). The spurges of Botswana. Botsw. Notes Rec..

[4] Lai X.Z., Yang Y.B., Shan X.U.L. (2004). The investigation of Euphorbiaceus medicinal plants in Southern China. Econ. Bot..

[5] Ernst M., Grace O.M., Saslis-Lagoudakis C.H., Haris S.L., Niclas N., Henrik T., Nina R. (2015). Global medicinal uses of *Euphorbia* L. (*Euphorbiaceae*). J. Ethnopharmacol..

[6] Mwine J., Van Damme P. (2011). Why do *Euphorbiaceae* tick as medicinal plants? A review of the *Euphorbiaceae* family and its medicinal features. J. Med. Plant. Res..

[7] Zeghad F., Djilani S.E., Djilani A., Dicko A. (2016). Antimicrobial and antioxidant activities of three *Euphorbia* species. Turk. J. Pharm. Sci..

[8] Vasas A., Rédei D., Csupor D., Molnár J., Hohmann J. (2012). Diterpenes from European *Euphorbia* species serving as prototypes for natural-product-based drug discovery. Eur. J. Org. Chem..

[9] Vasas A., Hohmann J. (2014). *Euphorbia* diterpenes: Isolation, structure, biological activity, and synthesis (2008−2012). Chem. Rev..

[10] Islam M.T., Mata O., Aguiar R.S., Paz M.C.J., Alencar M.B., Melo-cavalcante A.C. (2016). Therapeutic potential of essential oils focusing on diterpenes. Phytother. Res..

[11] Scholz A., Engler A. (1964). Euphorbiaceae. Syllabus der Planzenfamilien.

[12] Webster G.L. (1994). Classification of the *Euphorbiaceae*. Ann. Mol. Bot. Gar..

[13] Shi Q.W., Su X.H., Kiyota H. (2008). Chemical and pharmacological research of the plants in genus Euphorbia. Chem. Rev..

[14] Jin Y.X., Shi L.L., Zhang D.P., Wei H.Y., Si Y., Ma G.X., Zhang J. (2019). A review on daphnane-type diterpenoids and their bioactive studies. Molecules.

[15] Jury L., Reynolds T., Cutler F., Evans F. (1987). The Euphorbiales: Chemistry, Taxonomy, and Economic Botany.

[16] Gras J. (2013). Ingenol mebutate: A new option for actinic keratosis treatment. Drugs Today.

[17] Hanson J.R., Charlwood B.V., Banthorpe D.V. (1991). Diterpenoids: Methods in Plant Biochemistry.

[18] Cheng A., Lou Y., Mao Y., Lu S., Wang L., Chen X. (2007). Plant terpenoids: Biosynthesis and ecological functions. J. Integr. Plant. Biol..

[19] Dewick M. (2006). Medicinal Natural Products: A Biosynthetic Approach.

[20] Goel G., Makkar S., Francis G., Becker K. (2007). Phorbol esters: Structure, biological activity, and toxicity in animals. Int. J. Toxicol..

[21] Wang H.B., Wang X.Y., Liu L.P., Qin G.W., Kang T.G. (2015). Tigliane Diterpenoids from the Euphorbiaceae and Thymelaeaceae Families. Chem. Rev..

[22] Wongrakpanich W., Purin C. (2018). Induction of apoptosis in cancer cells by plants in the genus Euphorbia. Thai Bull. Pharm. Sci..

[23]  Salehi B., Iriti M., Vitalini S., Antolak H., Pawlikowska E., Kręgiel D.,  Sharifi-Rad J.,  Oyeleye I.,  Ademiluyi O.,  Czopek K. (2019). *Euphorbia*-derived natural products with potential for use in health maintenance. Biomolecules.

[24] Kemboi D., Peter X., Langat M., Tembu J. (2020). A review of the ethnomedicinal uses, biological activities, and Triterpenoids of *Euphorbia* Species. Molecules.

[25] Riahi F., Dashti N., Ghanadian M., Aghaei M., Faez F., Jafari S.M., Zargar N. (2020). Kopetdaghinanes, pro-apoptotic hemiacetialic cyclomyrsinanes from *Euphorbia kopetdaghi*. Fitoterapia.

[26] Elshamy A.I., Mohamed T.A., Al-Rowaily S.L., Abd-ElGawad A.M., Dar B.A., Shahat A.A., Hegazy M.E.F. (2019). Euphosantianane E-G: Three new premyrsinane type diterpenoids from *Euphorbia sanctae-catharinae* with the contribution to chemotaxonomy. Molecules.

[27] Flores-Giubi M.E., Durán-Pena M.J., Botubol-Ares J.M., Escobar-Montano F., Zorrilla D., Macías-Sánchez A.J., Hernández-Galán R. (2017). Gaditanone, a diterpenoid based on an unprecedented carbon skeleton isolated from *Euphorbia gaditana*. J. Nat. Prod..

[28] Bin Muhsinah A., Eko Nugroho A., Li H., Lazzaro S., DaSilva N.A., Li D., Ma H., Alsayari A., Morita H., Liu Y. (2020). Saudiarabicains A-E, bioactive 19-acetoxyingol diterpenoids from *Euphorbia saudiarabica*. Tetrahedron Lett..

[29] Hasan A., Liu G.Y., Hu R., Aisa H.A. (2019). Jatrophane Diterpenoids from *Euphorbia glomerulans*. J. Nat. Prod..

[30] Zhang J., He J., Cheng Y.C., Zhang P.C., Yan Y., Zhang H.J., Zhang W.K., Xu J.K. (2019). Fischernolides A-D, four novel diterpene-based meroterpenoid scaffolds with antitumor activities from: *Euphorbia fischeriana*. Org. Chem. Front..

[31] Wang S., Li H., Liu D., Zhao Q., Yang T., Li R., Chen X. (2020). Diterpenoids from the Seeds of *Euphorbia lathyris* and their in Vitro Anti-HIV Activity. Chem. Nat. Compd..

[32] Ye Y., Liu G.H., Dawa D., Ding L.S., Cao Z.X., Zhou Y. (2020). Cytotoxic diterpenoids from the roots of *Euphorbia stracheyi*. Phytochem. Lett..

[33] Shaker S., Sang J., Yan X.L., Fan R.Z., Tang G.H., Xu Y.K., Yin S. (2020). Diterpenoids from *Euphorbia royleana* reverse P-glycoprotein-mediated multidrug resistance in cancer cells. Phytochemistry.

[34] Yuan W.J., Gao W.F., Zhao J.Y., Zhang Y., Chen D.Z., Li S.L., Di Y.T., Hao X.J. (2020). Diterpenes with the potential treatment of vitiligo from the aerials parts of *Euphorbia antiquorum* L.. Fitoterapia.

[35] Yan X.L., Fan R.Z., Sang J., Xie X.L., Tang G.H., Yin S. (2020). Euphanoids A, and B, two new lathyrane diterpenoids with nitric oxide (NO) inhibitory activity from *Euphorbia kansuensis*. Nat. Prod. Res..

[36] Yan X.L., Sang J., Chen S.X., Li W., Tang G.H., Gan L.S., Yin S. (2019). Euphorkanlide A, a highly modified ingenane diterpenoid with a C24 appendage from *Euphorbia kansuensis*. Org. Lett..

[37] Song Q.Q., Rao Y., Tang G.H., Sun Z.H., Zhang J.S., Huang Z.S., Yin S. (2019). Tigliane diterpenoids as a new type of antiadipogenic agents inhibit GRα-Dexras1 axis in adipocytes. J. Med. Chem..

[38] Chen Y.N., Lu Q.Y., Li D.M., Li Y.Y., Pu X.X., Li B.T., Tang X.H., Tang H.Y., Liu S., Yang L. (2020). Three new diterpenoids from *Euphorbia peplus*. Nat. Prod. Res..

[39] Ghanadian M., Choudhary M.I., Ayatollahi A.M., Mesaik M.A., Abdalla O.M., Afsharypour S. (2013). New cyclomyrsinol diterpenes from *Euphorbia aellenii* with their immunomodulatory effects. J. Asian Nat. Prod. Res..

[40] Zhang X.D., Ni W., Yan H., Li G.T., Zhong H.M., Li Y., Liu H.Y. (2014). Daphnane-type diterpenoid glucosides and further constituents of *Euphorbia pilosa*. Chem. Biodivers..

[41] Zhang Y., Fan R.Z., Sang J., Tian Y.J., Chen J.Q., Tang G.H., Yin S. (2020). Ingol diterpenoids as P-glycoprotein-dependent multidrug resistance (MDR) reversal agents from *Euphorbia marginata*. Bioorg. Chem..

[42] Yan S.L., Li Y.H., Chen X.Q., Liu D., Chen C.H., Li R.T. (2018). Diterpenes from the stem bark of *Euphorbia neriifolia* and their in vitro anti-HIV activity. Phytochemistry.

[43] Wang S.Y., Li G.Y., Zhang K., Wang H.Y., Liang H.G., Huang C., Huang J., Wang J.H., Yang B.F. (2019). New ingol-type diterpenes from the latex of *Euphorbia resinifera*. J. Asian Nat. Prod. Res..

[44] Yan X.L., Huang J.L., Tang Y.Q., Tang G.H., Yin S. (2020). Euphopanes A–C, three new diterpenoids from *Euphorbia pekinensis*. Nat. Prod. Res..

[45] Wei W.J., Qi W., Gao X.M., Feng K.N., Ma K.L., Li H.Y., Li Y., Gao K. (2019). Anti-inflammatory evaluation and structure-activity relationships of diterpenoids isolated from *Euphorbia hylonoma*. Bioorg. Chem..

[46] Liu S.N., Hu J., Tan S.H., Wang Q., Xu J., Wang Y., Yuan Y., Gu Q. (2017). Ent -rosane diterpenoids from *Euphorbia milii* showing an Epstein-Barr virus lytic replication assay. RSC Adv..

[47] Li H., Yang P., Zhang E.H., Kong L.M., Meng C.Y. (2020). Antimicrobial ent-abietane-type diterpenoids from the roots of *Euphorbia wallichii*. J. Asian Nat. Prod. Res..

[48] Li L., Li D., Wang C., Feng L., Yu Z., Ning J., Zhang B., Zhang H., Wang C., Ma X. (2020). Aromatic rosane diterpenoids from the roots of *Euphorbia ebracteolata* and their inhibitory effects against lipase. Bioorg. Chem..

[49] Zhou B., Wu Y., Dalal S., Cassera M.B., Yue J.M. (2016). Euphorbesulins A-P, Structurally diverse diterpenoids from *Euphorbia esula*. J. Nat. Prod..

[50] Zhou M., Ma Q., He L., Chen Y.H., Zhu B.Y., Wang J.H., Yang Q., Liu S., Ma L.M. (2020). Cytotoxic jatrophane diterpenoids from the aerial parts of *Euphorbia helioscopia*. J. Asian Nat. Prod. Res..

[51] Zhang B.Y., Yin H.X., Zhang D.J. (2017). Two New ent-labdane diterpenes from the roots of *Euphorbia yinshanica*. Chem. Nat. Compd..

[52] Tsai J.Y., Rédei D., Forgo P., Li Y., Vasas A., Hohmann J., Wu C.C. (2016). Isolation of phorbol esters from *Euphorbia grandicornis* and evaluation of protein kinase C- and human platelet-activating effects of *Euphorbiaceae diterpenes*. J. Nat. Prod..

[53] Fei D.Q., Le Dong L., Qi F.M., Fan G.X., Li H.H., Li Z.Y., Zhang Z.X. (2016). Euphorikanin A, a diterpenoid lactone with a fused 5/6/7/3 ring system from *Euphorbia kansui*. Org. Lett..

[54] Tran T.N., Sichaem J., Nguyen V.K., Nguyen H.H., Cao T.T., Nguyen T.P., Vo V.G., Niamnont N., Nguyen N.H., Duong T.H. (2020). New diterpenoids from the stems of *Euphorbia antiquorum* growing in Vietnam. Nat. Prod. Res..

[55] Tran T.N., Sichaem J., Nguyen V.K., Chavasiri W., Niamnont N., Jongaramruong J., Duong T.H. (2021). A new ent-atisane diterpenoid from the aerial parts of *Euphorbia antiquorum* L.. Nat. Prod. Res..

[56] An L., Liang Y., Yang X., Wang H., Zhang J., Tuerhong M., Li D., Wang C., Lee D., Xu J. (2019). NO inhibitory diterpenoids as potential anti-inflammatory agents from *Euphorbia antiquoru*. Bioorg. Chem..

[57] Dai L.F., Liang Q., Liu T., He M.Y., Zhao P., Xu W.H. (2016). A new tigliane-type diterpene from *Euphorbia dracunculoides* Lam. Nat. Prod. Res..

[58] Wang L., Zang Z., Wang Y.F., Huang S.X., Cao P., Zhao Y. (2015). Two new myrinsol diterpenoids from *Euphorbia dracunculoides* Lam. Chin. Chem. Lett..

[59] Xie X.L., Fan R.Z., Hu R., Luo S.Y., Tang G.H., Yin S. (2020). Euphoresulanes A–M, structurally diverse jatrophane diterpenoids from *Euphorbia esula*. Bioorg. Chem..

[60] Xie R., Li L., Fan X., Zi J. (2020). Euphoractone, a cytotoxic meroterpenoid with an unusual ent-abietane-phloroglucinol skeleton, from *Euphorbia fischeriana* Steud. Chin. Chem. Lett..

[61] Li M., He F., Zhou Y., Wang M., Tao P., Tu Q., Lv G., Chen X. (2019). Three new ent-abietane diterpenoids from the roots of *Euphorbia fischeriana* and their cytotoxicity in human tumor cell lines. Arch. Pharm. Res..

[62] He J., Xu J.K., Zhang J., Bai H.J., Ma B.Z., Cheng Y.C., Zhang W.K. (2019). Fischeriana A, a meroterpenoid with an unusual 6/6/5/5/5/6/6 heptacyclic carbon skeleton from the roots of *Euphorbia fischeriana*. Org. Biomol. Chem..

[63] Du K., Yang X., Li J., Meng D. (2020). Antiproliferative diterpenoids and acetophenone glycoside from the roots of *Euphorbia fischeriana*. Phytochemistry.

[64] Su J.C., Cheng W., Song J.G., Zhong Y.L., Huang X.J., Jiang R.W., Li Y.L., Li M.M., Ye W.C., Wang Y. (2019). Macrocyclic diterpenoids from *Euphorbia helioscopia* and their potential anti-inflammatory activity. J. Nat. Prod..

[65] Meng X.H., Wang K., Chai T., Guo Z.Y., Zhao M., Yang J.L. (2020). Ingenane and jatrophane diterpenoids from *Euphorbia kansui* and their antiproliferative effects. Phytochemistry.

[66] Ma L.F., Zhang Y., Zhang X., Chen M.J., Zhan Z.J., Shan W.G. (2020). A new tetracyclic diterpenoid from the seeds of *Euphorbia lathyris*. J. Chem. Res..

[67] Xu W.D., Tian Y., Guo Q.L., Yang Y.C., Shi J.G. (2014). Secoeuphoractin, a minor diterpenoid with a new skeleton from *Euphorbia micractina*. Chin. Chem. Lett..

[68] Qi W.Y., Gao X.M., Ma Z.Y., Xia C.L., Xu H.M. (2020). Antiangiogenic activity of terpenoids from *Euphorbia neriifolia* Linn. Bioorg. Chem..

[69] Li J.C., Feng X.Y., Liu D., Zhang Z.J., Chen X.Q., Li R.T., Li H.M. (2019). Diterpenoids from *Euphorbia neriifolia* and their related anti-HIV and cytotoxic activity. Chem. Biodivers..

[70] Li J.C., Zhang Z.J., Yang T., Jiang M.Y., Liu D., Li H.M., Li R.T. (2019). Six new ent-abietane-type diterpenoids from the stem bark of *Euphorbia neriifolia*. Phytochem. Lett..

[71] Du M., An L., Xu J., Guo Y. (2020). Euphnerins A, and B, diterpenoids with a 5/6/6 rearranged spirocyclic carbon skeleton from the stems of *Euphorbia neriifolia*. J. Nat. Prod..

[72] Wan L.S., Chu R., Peng X.R., Zhu G.L., Yu M.Y., Li L., Zhou L., Lu S.Y., Dong J.R., Zhang Z.R. (2016). Pepluane and paraliane diterpenoids from *Euphorbia peplus* with potential anti-inflammatory activity. J. Nat. Prod..

[73] Li J.-C., Dai W.-F., Liu D., Jiang M.-Y., Zhang Z.-J., Chen X.-Q., Chen C.-H., Li R.-T., Li H.-M. (2020). Bioactive ent-isopimarane diterpenoids from *Euphorbia neriifolia*. Phytochemistry.

[74] Xu J., Yang B., Fang L., Wang S., Guo Y., Yamakuni T., Ohizumi Y. (2013). Four new myrsinol diterpenes from *Euphorbia prolifera*. J. Nat. Med..

[75] Wang P., Xie C., An L., Yang X., Xi Y., Yuan S., Zhang C., Tuerhong M., Jin D.Q., Lee D. (2019). Bioactive diterpenoids from the stems of *Euphorbia royleana*. J. Nat. Prod..

[76] Demetzos C., Dimas K.S. (2001). Labdane-type diterpenes: Chemistry and biological activity. Stud. Nat. Prod. Chem..

[77] Liu M., Wang W.G., Sun H.D., Pu J.X. (2017). Diterpenoids from *Isodon* species: An update. Nat. Prod. Rep..

[78] Wu Q.C., Tang Y.P., Ding A.W., You F.Q., Zhang L., Duan J.A. (2009). ^13^C-NMR data of three important diterpenes isolated from *Euphorbia* species. Molecules.

[79] Satti N.K., Suri O.P., Dhar K.L., Kawasaki T., Miyahara K., Noda N. (1987). Diterpenes from *Euphorbia fidjiana*. J. Nat. Prod..

[80] Lal A.R., Cambie R.C., Rutledge P.S., Woodgate P.D. (1990). Ent-pimarane and ent-abietane diterpenes from *Euphorbia fidjiana*. Phytochemistry.

[81] Haba H., Lavaud C., Magid A.A., Benkhaled M. (2009). Diterpenoids, and triterpenoids from *Euphorbia retusa*. J. Nat. Prod..

[82] Haba H., Lavaud C., Marcourt L., Long C., Harkat H., Benkhaled M. (2009). Ent-abietane diterpenoids from *Euphorbia guyoniana* Boiss. & Reut. Biochem. Syst. Ecol..

[83] Yu C.C., Hsieh C.R., Hsiao G., Chen P.Y., Chang M.L., Yin H.W., Lee T.H., Lee C.K. (2012). Regulated expressions of MMP-2, -9 by diterpenoids from *Euphorbia formosana* hayata. Molecules.

[84] Geris R., Simpson T.J. (2009). Meroterpenoids produced by fungi. Nat. Prod. Rep..

[85] Baloch I.B., Baloch M.K., Baloch A.K. (2010). Schistosomiasis suppressing deoxyphorbol esters from *Euphorbia cauducifolia* L. Latex. Planta Med..

[86] Liu J.H., Latif A., Ali M., Zhang G.P., Xiang W.J., Ma L., Arfan M., Hu L.H. (2012). Diterpenoids from *Euphorbia neriifolia*. Phytochemistry.

[87] Satti N.K., Suri O.P., Thaper R.K., Kachroo P.L. (1988). *Ent*-atisane-3β, 16α, 17-triol, a diterpene from E. acaulis. Phytochemistry.

[88] Mbwambo Z.H., Lee S.K., Mshiu E.N., Pezzuto J.M., Kinghorn A.D. (1996). Constituents from the stem wood of *Euphorbia quinquecostata* with phorbol dibutyrate receptor-binding inhibitory activity. J. Nat. Prod..

[89] Drummond G.J., Grant P.S., Brimble M.A. (2020). ent-atisane diterpenoids: Isolation, structure, and bioactivity. Nat. Prod. Rep..

[90] Wang H., Zhang X., Zhou Y., Peng S., Zhou D., Ding L. (2008). Two new diterpenes from *Euphorbia kansuensis*. Fitoterapia.

[91] Li X.L., Li Y., Wang S.F., Zhao Y.L., Liu K.C., Wang X.M., Yang Y.P. (2009). Ingol and ingenol diterpenes from the aerial parts of *Euphorbia royleana* and their antiangiogenic activities. J. Nat. Prod..

[92] Liang Q.L., Dai C.C., Jiang J.H., Tang Y.P., Duan J.A. (2009). A new cytotoxic casbane diterpene from *Euphorbia pekinensis*. Fitoterapia.

[93] Su B.N., Park E.J., Mbwambo Z.H., Santarsiero B.D., Mesecar A.D., Fong H.H.S., Pezzuto J.M., Kinghorn A.D. (2002). New chemical constituents of *Euphorbia quinquecostata* and absolute configuration assignment by a convenient Mosher ester procedure carried out in NMR tubes. J. Nat. Prod..

[94] Appendino G., Belloro E., Tron G.S.C., Jakupovic J., Ballero M. (2002). Pentacyclic diterpenoids from *Euphorbia characias*. Fitoterapia.

[95] Tran Q.T.N., Wong W.S.F., Chai C.L.L. (2017). Labdane diterpenoids as potential anti-inflammatory agents. Pharmacol. Res..

[96] Kubo I., Xu Y., Shimizu K. (2004). Antibacterial Activity of ent-kaurene diterpenoids from Rabdosia rosthornii. Phyther. Res..

[97] Kusumoto N., Ashitani T., Murayama T., Ogiyama K., Takahashi K. (2010). Antifungal abietane-type diterpenes from the cones of *Taxodium distichum* Rich. J. Chem. Ecol..

[98] Schmid T.R.J. (1987). The biosynthesis of tigliane and related diterpenoids; an intriguing problem. J. Linn. Soc. Bot..

[99] Li W., Tang Y.Q., Chen S.X., Tang G.H., Gan L.S., Li C., Rao Y., Huang Z.S., Yin S. (2019). Euphorhelipanes A, and B, triglyceride-lowering euphorbia diterpenoids with a bicycle [4.3.0] nonane core from *Euphorbia helioscopia*. J. Nat. Prod..

[100] Yuan S., Hua P., Zhao C., Zhou H., Xu J., Xu J., Gu Q. (2020). Jatrophane diterpenoids from *Euphorbia esula* as inhibitors of RANKL-induced osteoclastogenesis. J. Nat. Prod..

[101] Devappa R.K., Makkar H.P.S., Becker K. (2011). Jatropha diterpenes: A review, JAOCS, J. Am. Oil Chem. Soc..

[102] Adelakun T.A., Ding X., Ombati R.M., Zhao N.D., Obodozie-Ofoegbu O.O., Di Y.T., Zhang Y., Hao X.J. (2020). A new highly oxygenated abietane diterpenoid and a new lysosome generating phorbol ester from the roots of *Euphorbia fischeriana* Steud. Nat. Prod. Res..

[103] Zhang C.Y., Wu Y.L., Zhang P., Chen Z.Z., Li H., Chen L.X. (2019). Anti-inflammatory lathyrane diterpenoids from *Euphorbia lathyris*. J. Nat. Prod..

[104] Tian Y., Guo Q., Xu W., Zhu C., Yang Y., Shi J. (2014). A minor diterpenoid with a new 6/5/7/3 fused-ring skeleton from *Euphorbia micractina*. Org. Lett..

[105] Wang W., Wu Y., Li C., Yang Y., Li X., Li H., Chen L. (2020). Synthesis of new lathyrane diterpenoid derivatives from *Euphorbia lathyris* and evaluation of their anti-inflammatory activities. Chem. Biodivers..

[106] Yang T., Wang S., Li H., Zhao Q., Yan S., Dong M., Liu D., Chen X., Li R. (2020). Lathyrane diterpenes from *Euphorbia lathyris* and the potential mechanism to reverse the multi-drug resistance in HepG2/ADR cells. Biomed. Pharmacother..

[107] Wang L., Ma Y.T., Sun Q.Y., Zang Z., Yang F.M., Liu J.P., Jiang J.H., Huang S.X., Zhao Y. (2016). A new lathyrane diterpenoid ester from *Euphorbia dracunculoides*. Chem. Nat. Compd..

[108] Aleksandrov M., Maksimova V., Koleva Gudeva L. (2019). Review of the anticancer and cytotoxic activity of some species from genus *Euphorbia*. Agric. Conspec. Sci..

[109] Yan S.S., Li Y., Wang Y., Shen S.S., Gu Y., Wang H.B., Qin G.W., Yu Q. (2008). 17-acetoxyjolkinolide B irreversibly inhibits IkappaB kinase and induces apoptosis of tumor cells. Mol. Cancer Ther..

[110] Baloch I.B., Baloch M.K., Saqib Q.N. (2005). Tumor-promoting diterpene esters from the latex of *Euphorbia cauducifolia* L.. Helv. Chim. Acta.

[111] Kulig J., Sehl T., Mackfeld U., Wiechert W., Pohl M., Rother D. (2019). An enzymatic 2-step cofactor and co-product recycling cascade towards a chiral 1,2-diol. Part I: Cascade design. Adv. Synth. Catal..

[112] Krstić G., Jadranin M., Stanković M., Aljančić I., Vujisić L., Mandić B., Tešević V. (2019). Jatrophane diterpenoids with a protective effect on human lymphocytes DNA. Nat. Prod. Commun..

[113] Zolfaghari B., Yazdiniapour Z., Ghanadian M., Lanzotti V. (2016). Cyclomyrsinane and premyrsinane diterpenes from *Euphorbia sogdiana* Popov. Tetrahedron.

[114] Corea G., Di Pietro A., Dumontet C., Fattorusso E., Lanzotti V. (2009). Jatrophane diterpenes from *Euphorbia* spp. as modulators of multidrug resistance in cancer therapy. Phytochem. Rev..

[115] Barile E., Gabriella C., Virginia L. (2008). Diterpenes from *Euphorbia* as potential leads for drug design. Nat. Prod. Commun..

[116] Bani S., Kaul A., Jaggi B.S., Suri K.A., Suri O.P., Sharma O.P. (2000). Anti-inflammatory activity of the hydrosoluble fraction of *Euphorbia royleana* latex. Fitoterapia.

[117] Wang B., Wei Y., Zhao X., Tian X., Ning J., Zhang B., Deng S., Li D., Ma X., Wang C. (2018). Unusual ent-atisane type diterpenoids with 2-oxopropyl skeleton from the roots of *Euphorbia ebracteolata* and their antiviral activity against human rhinovirus 3 and enterovirus. Bioorg. Chem..

